# Estimating an individual’s oxygen uptake during cycling exercise with a recurrent neural network trained from easy-to-obtain inputs: A pilot study

**DOI:** 10.1371/journal.pone.0229466

**Published:** 2020-03-12

**Authors:** Andrea Zignoli, Alessandro Fornasiero, Matteo Ragni, Barbara Pellegrini, Federico Schena, Francesco Biral, Paul B. Laursen

**Affiliations:** 1 CeRiSM Research Centre, University of Verona, Rovereto (TN), Italy; 2 Department of Neuroscience, Biomedicine and Movement, University of Verona, Verona, Italy; 3 Department of Industrial Engineering, University of Trento, Trento, Italy; 4 Sports Performance Research Institute NZ, Auckland University of Technology, Auckland, New Zealand; University of Calgary, CANADA

## Abstract

Measurement of oxygen uptake during exercise ($\dot{V}O_{2}$) is currently non-accessible to most individuals without expensive and invasive equipment. The goal of this pilot study was to estimate cycling $\dot{V}O_{2}$ from easy-to-obtain inputs, such as heart rate, mechanical power output, cadence and respiratory frequency. To this end, a recurrent neural network was trained from laboratory cycling data to predict $\dot{V}O_{2}$ values. Data were collected on 7 amateur cyclists during a graded exercise test, two arbitrary protocols (Prot-1 and -2) and an “all-out” Wingate test. In Trial-1, a neural network was trained with data from a graded exercise test, Prot-1 and Wingate, before being tested against Prot-2. In Trial-2, a neural network was trained using data from the graded exercise test, Prot-1 and 2, before being tested against the Wingate test. Two analytical models (Models 1 and 2) were used to compare the predictive performance of the neural network. Predictive performance of the neural network was high during both Trial-1 (MAE = 229(35) mlO_2_min^-1^, r = 0.94) and Trial-2 (MAE = 304(150) mlO_2_min^-1^, r = 0.89). As expected, the predictive ability of Models 1 and 2 deteriorated from Trial-1 to Trial-2. Results suggest that recurrent neural networks have the potential to predict the individual $\dot{V}O_{2}$ response from easy-to-obtain inputs across a wide range of cycling intensities.

## 1 Introduction

Aerobic metabolism, measured universally via oxygen uptake ($\dot{V}O_{2}$) [1], is the principal mechanism by which humans generate energy from ingested foodstuffs for life. Physical activity demands additional O_2_ to working muscles, which is matched by O_2_ delivery from the cardiopulmonary system to limit reliance on the less efficient anaerobic pathways. The $\dot{V}O_{2}$ kinetics, the maximal $\dot{V}O_{2}$ attainable (i.e. $\dot{V}O_{2MAX}$) and the $\dot{V}O_{2}$ required for sub-maximal activities, are highly related to health, fitness and exercise performance [2,3]. Direct measurement of $\dot{V}O_{2}$ requires expensive, invasive and fragile instrumentation, such as metabolimeters [4]. As a consequence, the study of exercising $\dot{V}O_{2}$ is mostly confined to laboratories and clinics. During outdoor activities, wearing and carrying a metabolimeter can put the athletes and the instrumentation in danger. Therefore, estimating $\dot{V}O_{2}$ without reliance on a metabolimeter would be highly useful for a number of performance assessment applications.

Typically, when a metabolimeter is not available, the steady value of $\dot{V}O_{2}$ (i.e. $\dot{V}O_{2ss}$) is estimated from heart rate (HR). However, this methodology has limitations [5]. For example, for very low and very high intensity exercises, the $\mathrm{HR}/\dot{V}O_{2}$ relationship is not linear. Furthermore, heart rate is affected by a high day-to-day variability [6]. However, another method we might use to directly estimate $\dot{V}O_{2}$ is through its relationship with mechanical power output (P). Indeed, cycling exercise is a repetitive and easily testable activity in which the mechanical power output can be measured directly and reliably using a power meter [7] and even estimated using simple energetic relationships [8].

**Fig 1:**
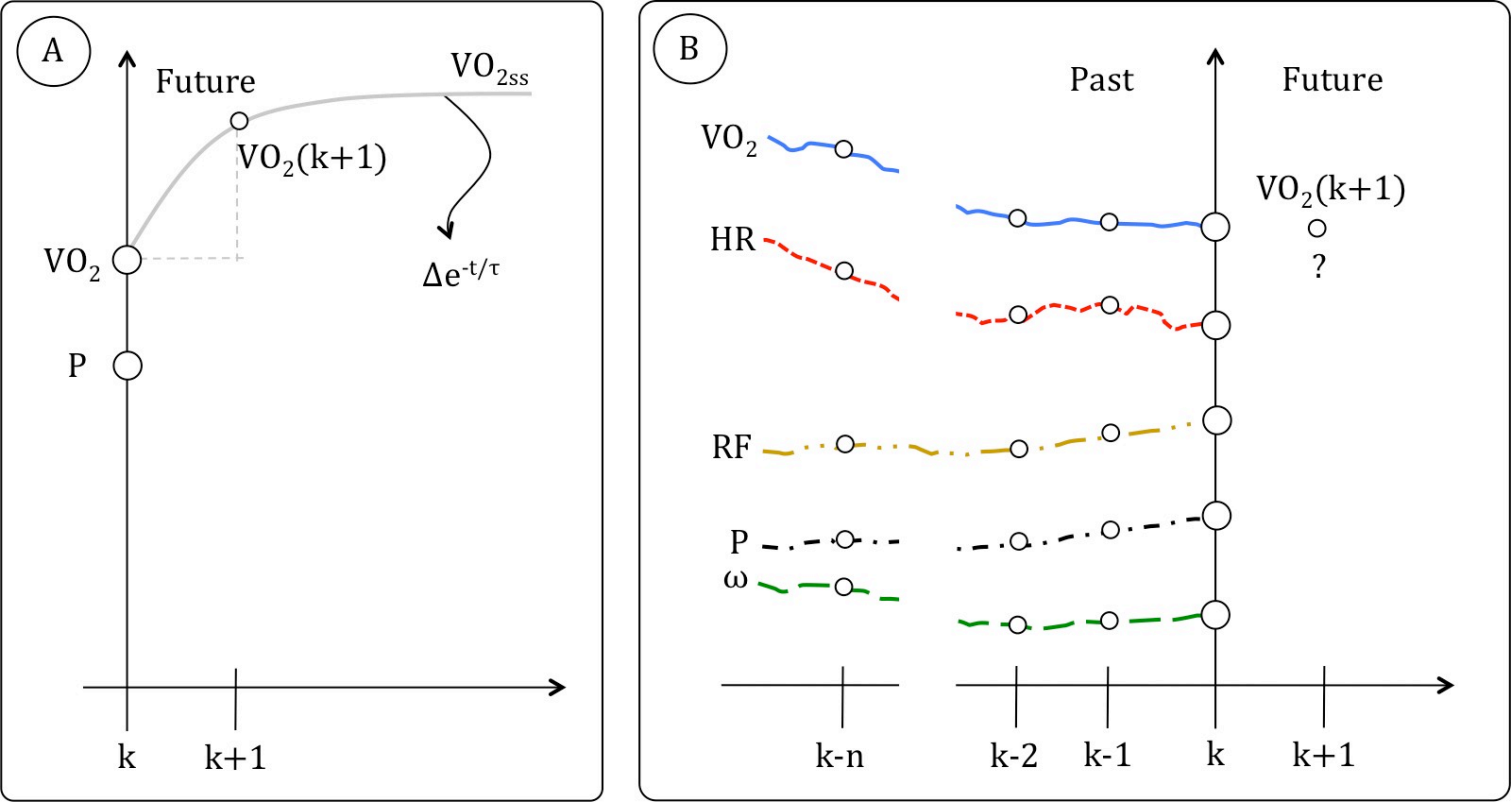
A schematization of the analytical equations approach (A) and artificial intelligence (AI) approach (B) is given. A: the current value of the power (P) and oxygen uptake ($\dot{V}O_{2}$) (i.e. P(k) and $\dot{V}O_{2}$ (k) respectively) are used in a formula (i.e. Δe^-t/τ^, with Δ calculated as the difference between the $\dot{V}O_{2}$ steady state $\dot{V}O_{2ss}$ and the current $\dot{V}O_{2}$) to forecast future values of $\dot{V}O_{2}$ (i.e. $\dot{V}O_{2}(k+1)$). B: In an AI approach to the time-series problem, current (k) and past values (k-1, k-2, … k-n) of heart rate (HR), P and cadence (ω) are used to forecast future values $\dot{V}O_{2}(k+1)$.

However, like heart rate, $\dot{V}O_{2}$ does not respond promptly to mechanical power output variations and $\dot{V}O_{2}$ dynamics must be taken into account [9]. The three distinct phases involved with $\dot{V}O_{2}$ dynamics include: 1) a cardio-dynamic phase-I, 2) a fundamental phase-II and 3) a slow phase-III component. Whilst phase-I and II are always present in responses to step-changes in the workload, the phase-III only becomes discernible at heavy and severe exercise intensities [10]. If the $\dot{V}O_{2}$ dynamic is considered to be linear (this assumption has been questioned multiple times [11–13]), a first-order model can be used to roughly approximate the $\dot{V}O_{2}$ at the next instant k+1 (i.e. $\dot{V}O_{2}$(k+1)) from $\dot{V}O_{2}$ and mechanical power output (in Watt) at the current instant k (i.e. $\dot{V}O_{2}(k)$ and P(k)) (Fig 1A):

$$\dot{V}O_{2}(k+1)=\frac{\left(P(k)∙G+{\dot{V}O}_{2R}-{\dot{V}O}_{2}(k))\Delta t(k\right)}{\tau }+{\dot{V}O}_{2}(k)$$

where G is the $\dot{V}O_{2}$ “gain” [14], $\dot{V}O_{2R}$ is the resting $\dot{V}O_{2}$ [15] and Δt(k) is the time that separates the two instants k and k+1. This formulation has some shortcomings, e.g.: 1) changes of G and τ across exercise intensity domains [16] (or with transitions from greater baseline intensities [17]) and cadences (ω) [18], 2) prolonged exercise affects the relationship between G and P [19] and 3) $\dot{V}O_{2}$ response to exercise is affected by recent exercise history [20]. Such a description can be improved including those features known to be relevant or related to $\dot{V}O_{2}$, e.g.: current and past values of mechanical power output, pedalling cadence, heart rate and respiratory frequency (RF).

The problem of forecasting $\dot{V}O_{2}$ data starting from observations of other variables taken sequentially in time can be considered as a time series prediction problem. Analytical equations of the dynamics have limited capacity to accurately model such complex data without requiring very large and complex formulations. An alternative approach may be found among the artificial intelligence technologies [21]. Particularly, machine learning algorithms can be used to learn from data and individuate patterns of variation between variables (Fig 1B). A machine learning algorithm that considers time sequences can be implemented by means of the so-called artificial neural networks, a biologically-inspired computational system that mathematically formalizes the connections among and within layers of artificial neurons [22]. Artificial neurons receive one or more inputs and sums them to provide an output. Inside the neuron, each input is weighted, and the sum is passed through a non-linear activation function. Given a sufficient number of layers and neurons, a neural network can always be trained (i.e. the weights of the neural network are calibrated) to approximate a real relation between inputs and outputs [23].

Examples of the application of artificial intelligence to time series problems include financial time series forecasting [24], as well as arrhythmia detection from ECG signals [25]. Recurrent neural network and, in particular long-short term memory [26], are suited for time series forecasting problems and sequences [27]. Unlike feed-forward neural network, recurrent neural networks make use of an internal memory to process sequence of inputs. This is a very important property when the prediction of the neural network depends on the historical context of inputs.

With respect to the field of cycling performance, for example, an artificial intelligence approach has been proposed for training data processing [28]. In the field of exercise physiology, a neural network has been developed [29] to model the heart rate versus mechanical power output relationship. With this neural network, it was possible to find the heart rate associated with the anaerobic threshold non-invasively in soccer players. In $\dot{V}O_{2}$ data estimation, Laitinen & Rasanen [30] used a neural network to estimate $\dot{V}O_{2}$ in children with congenital heart disease from inputs like heart rate and blood pressure. However, the accuracy attained suggested that the predictive power of their neural network was “insufficient” at that time. In 2017, Gonzalez et al., [31] presented an accurate mathematical description for $\dot{V}O_{2}$ dynamics during high-intensity variable cycling exercise, and the same authors suggested that a neural network could “perform even better” than their analytical model [32]. More recently, machine-learning has been used [33] to predict $\dot{V}O_{2}$ accurately during walking and with different daily activities [34], including cycling [35].

In light of the promises of the artificial intelligence technologies [21], the purpose of this pilot study was to predict the individual response of $\dot{V}O_{2}$ during high-intensity cycling exercise starting from easy-to-obtain inputs. We hypothesised that a recurrent neural network could provide accurate individualised predictions across a variety of exercise conditions. To highlight the potential of this methodology, we compared the predictive accuracy of the neural network with a first-order $\dot{V}O_{2}$ kinetics equation and a previously published higher-order model.

## 2 Methods

### 2.1 Experimental data

Seven recreational cyclists (6 males, Table 1) participated in the study and visited the laboratory on three separate occasions. The ethics committee of the Department of Neurological and Movement Sciences of the University of Verona approved the study.

**Table 1:** Participants’ characteristics: Mean (SD) of the weight, the maximal oxygen uptake ($\dot{V}O_{2MAX}$), the peak power output (PPO) and the three intensity-levels adopted in the second and third tests (P_1_, P_2_ and P_3_).

	Weight	$\dot{V}O_{2MAX}$	PPO	P_1_	P_2_	P_3_
Mean	76.0 (6.6) kg	4443 (720) mlO_2_min^-1^	335 (44) W	109 (21) W	246 (42) W	304 (43) W

The participants gave informed consent and the research was conducted in accordance with the declaration of Helsinki. All tests were performed on an electromagnetically-braked bicycle ergometer (Excalibur Sport, Lode). Measurements of mechanical power output and pedalling cadence were collected continuously. Respiratory measurements, such as $\dot{V}O_{2}$ and respiratory frequency were collected using breath-by-breath methods from an automated open-circuit gas analyser (Quark CPET, Cosmed). Immediately before every test session, the gas analyser and the flow meter were calibrated. Invalid breaths (i.e. those lying outside the ranges of respiratory frequency b/min 2–90 (min-max); ventilation (L) 0.100–10000, FeO_2_% (%) 5–20; FeCO_2_ (%) 1–10) were automatically removed in real-time by the CPET software. Heart rate was recorded continuously (beat-by-beat) during the test with a heart rate monitor incorporated into the gas analyser. Heart rate was interpolated and provided at the breath-by-breath time sequence by the metabolimeter.

During the first visit, participants underwent a graded exercise test (GXT) for aerobic assessment. $\dot{V}O_{2}$ and respiratory frequency data were averaged over 4 min at rest, to give a resting metabolic rate ($\dot{V}O_{2R}$) and resting respiratory frequency (RF_R_) respectively. Participants warmed up for 10 minutes at 85 W and freely chosen pedalling frequency. The graded exercise test started at a workload of 100 W for 4 min and, subsequently, the workload increased by 40 W every 4 min until exhaustion. The pedalling cadence during the test was kept constant at 90 rpm, using a monitor that provided participants with visual feedback. The peak power output (PPO) of the test was determined using the power of the last completed stage and the time of the last uncompleted stage [36]. The $\dot{V}O_{2MAX}$ and the maximal respiratory frequency RF_MAX_ were defined as the highest value of $\dot{V}O_{2}$ and respiratory frequency registered during the test over a 20-s rolling average [37]. The first ventilatory threshold (VT1) was determined from visual inspection of: 1) the first disproportionate increase in minute ventilation (VE); 2) an increase in $\mathrm{VE}/\dot{V}O_{2}$ with no increase in $\mathrm{VE}/\dot{V}CO_{2}$ (where $\dot{V}CO_{2}$ is the exhaled volume of CO_2_); 3) an increase in end-tidal O_2_ with no consequent fall in end-tidal CO_2_ tensions. The second ventilatory threshold (VT2) was determined from: 1) the second disproportionate increase in minute ventilation; 2) the first systematic increase in $\mathrm{VE}/\dot{V}CO_{2}$; 3) the first systematic decrease in end-tidal CO_2_ tension[38,39]. To account for the differences that exists in the $\dot{V}O_{2}$ versus power output relationships from graded versus constant exercise [9], the power values expected to elicit the $\dot{V}O_{2}$ associated with the first and second ventilatory thresholds were estimated using the equations established by Kuipers et al. [40]. We are aware that graded exercise testing protocols can influence the $\dot{V}O_{2}$ versus power output relationship [41], hence reducing the validity of the specific power values associated with first and the second ventilatory threshold, i.e.: P_VT1_ and P_VT2_, respectively. P_VT1_ and P_VT2_ were obtained considering the power output of the previously completed stage and the time of the completed stage when the ventilatory threshold occurred (e.g. 18 min = 240 W; 220 W (16 min) + 2 min/4min x 40 W).

Three different mechanical power output levels were defined for every participant as follows: moderate intensity P_1_ = 0.5*P_VT1_, heavy intensity P_2_ = 0.5*(P_VT2_-P_VT1_)+ P_VT1_ and severe intensity P_3_ = 0.5*(PPO-P_VT2_) + P_VT2_ (**Table 1**). After a recovery period of 1 hour, participants performed a 30” Wingate test (WG) on a mechanically braked cycle ergometer (Ergomedic 894-Ea, Monark). During this test, the highest mechanical power P_MAX_ and the maximal cadence ω_MAX_ were determined.

During the second and third visit to the laboratory, athletes performed a warm-up for 10 min at a constant power of 85 W and rested for 4 minutes, before performing two different protocols on separate days. The first protocol (Test 1) consisted of a constant mechanical power output of 100 W for 4 minutes, followed by three repetitions of three constant bouts of 4 minutes at P_2_, P_3_ and P_1_ (please see the additional material for a graphical representation of the protocol). The second protocol (Test 2) started with a linear increase in the mechanical power output (i.e. a ramp) from P_1_ to P_3_ in 4 min. The initial ramp was followed by: a 1-min bout at P_3_, a 4-min bout at P_1_, a 1-min ramp from P_1_ to P_2_, a constant bout of 3 min at P_2_, a 4-min bout at P_1_, a 2-min ramp from P_1_ to P_2_, a constant bout of 2 min at P_2_ and a 4-min bout at P_1_ (please see the additional material for a graphical representation of the protocol). These two arbitrary protocols were designed to elicit different $\dot{V}O_{2}$ dynamics behaviours and facilitate the convergence of the parameter estimation.

### 2.2 Dataset preparation

Metabolic and power data were synchronized in time in a post-processing phase. Particularly, mechanical power output and pedalling cadence signals were resampled to meet the same breath-by-breath frequency of the cardiopulmonary data. Data were normalized between 0 and 1, to facilitate convergence during parameter optimization [42]. $\dot{V}O_{2}$ data was set to 1 if it matched $\dot{V}O_{2MAX}$ and 0 if it matched $\dot{V}O_{2R}$. Respiratory frequency data was set to 1 if it matched RF_MAX_ and to 0 if it matched RF_R_. Mechanical power output data was set to 1 if it matched PPO and 0 if it matched zero, while pedalling cadence data was set to 1 if it matched ω_MAX_ and 0 if it matched zero.

Past input values were included and used for predicting the output values. As a result, the input **x** and the output **y** for our machine learning algorithms became:

$$x=[P_{k-n},\omega_{k-n},{HR}_{k-n},{RF}_{k-n},\ldots ,P_{k-1},\omega_{k-1},{HR}_{k-1},{RF}_{k-1},P_{k},\omega_{k},{HR}_{k},{RF}_{k}]y=[{\dot{V}O}_{2k+1}]$$

Therefore, the shape of the input was nx4, while the shape of the output was 1x1. This means that every single exercise provided several samples equal to the total number of breath N minus the number of past inputs n (i.e. N-n). While N was determined by the duration of the exercises, a value of n = 70 breaths was proposed as a good estimate of the time-dependence decay between the output and past values of inputs. This implied that the machine learning algorithms could hypothetically learn about relationships between inputs and outputs lasting across 70 breaths. This number was chosen because it provided the best combination between computational time and prediction accuracy.

The entire dataset was split into 2 sub-datasets: the training set and the test set. The training set included 3 of the 4 tests performed by every cyclist and was used to adjust the weights of the neural network. The test set included the remaining test and was used to confirm the actual predictive power of the neural network. In a first Trial (Trial 1), the training set included the graded exercise test, Wingate test and Test 2, while the test set included Test 1. In a second Trial (Trial 2), the training set included the graded exercise test, Test 1 and Test 2, while the test set included the Wingate test.

### 2.3 Neural network design

An artificial intelligence regressor was developed and used to predict values of $\dot{V}O_{2}$. The neural network was coded and implemented using Python (ver. 3.6, Python Software Foundation), a high-level programming language for general-purpose programming. In particular, the open-source library called Keras was adopted to specifically design and test the neural network. The neural network was created using a Tensorflow *backend* with *cuda* support (2xNVidia GT750M i74xxx). A summary of the model is given in **Table 2**.

**Table 2:** A total of 21717 parameters have been included in the LSTM NN designed in this study. Three LSTM layers of 32 neurons were used with 1 hidden layer of 10 feed-forward neurons and 1 output layer of 1 neuron. Input shape for LSTM layers were determined form the batch size (10), the number of past inputs considered in the time series (70) and the number of neurons of the layer.

Layer (type)	Output shape	N parameters
LSTM 1	10x70x32	4736
LSTM 2	10x70x32	8320
LSTM 3	10x32	8320
Dense 1	10x10	330
Dense 2	10x1	11

The neural network was composed with long-short term memory neurons [26], best suited for time-series analyses and sequence detection [27]. A total of 3 long-short term memory layers of 32 neurons each were formed, plus 1 hidden layer of 10 neurons and 1 output layer of 1 neuron. The neural network was trained with a stochastic gradient method (*adagrad*) that optimises a categorical cross entropy loss. The training dataset entries were shuffled and the whole dataset was crossed in 20 epochs. The weights were initialized as random values, while biases were initialised as random positive values. The batch size (that defines the number of samples propagated in the neural network) was set to 10.

There are no specific and scientifically proven steps to be followed in the design of the neural network. However, we know that the choice of the number of layers, the number of neurons, the number of epochs and the batch size affect the accuracy of the output and the computational time. Therefore, to select these parameters, we proceeded by trial and error, until the best combination of accuracy and computational time was found. The final architecture with 3 long-short term memory layers, has been shown to work well in other time-series classification problems using physiological data [43].

### 2.4 The analytical models

Two models for $\dot{V}O_{2}$ data prediction were used to test the relative predictive power of the neural network. The two candidate models were chosen as they have been already tested during the prediction of $\dot{V}O_{2}$ data from mechanical power output data in cycling [32].

Parameters of the models were calculated using a particle swarm optimization algorithm [44], with the goal of finding those model parameters that could lead to the best match with experimental data (in the least square sense). The number of iterations was fixed at 250, a number that was found to provide stable solution in a reasonable amount of time. The particle swarm optimization algorithm was implemented and run in the Matlab (ver. 2017a, Mathworks) numerical environment as follows:

1. Model 1: the $\dot{V}O_{2}$ dynamics were approximated using the equation offered in the Introduction.
2. Model 2: the complete description of this model can be found in the original article [31]. Dynamics equations of the model are reported in the Appendix using a formulation best suited for spreadsheets.

### 2.5 Statistics

To assess the prediction ability of the different models, a residual analysis was conducted. Residuals were calculated as the difference between the experimental $\dot{V}O_{2}$ values and the output $\dot{V}O_{2}$ values predicted by the models. Mean absolute error (MAE) and root mean squared error (RMSE) of the residuals were calculated. A regression analysis of the residuals provided a Pearson’s correlation coefficient *r* and variance explained *R*^*2*^ statistic from the fit of each output. A Bland-Altman analysis [45] was used to assess the level of agreement between measured and predicted data. The mean bias and the limits of agreement at 95% of probability LA_95%_ were calculated. The bias was significant if the equality line fell outside the confidence intervals CI_95%_ of the mean bias for the sample. The confidence limits of the mean bias were calculated with the significance level set to 0.05. Additionally, best practice suggested we define *a priori* a significant and meaningful level of maximal acceptable limits. This limit was set to 200 mlO_2_min^-1^, which, in our experience, is comparable with the magnitude of the typical noise underlying $\dot{V}O_{2}$ measurements at high exercise intensities.

An autocorrelation analysis calculated the strength of the relationship between a residual and residuals at prior time steps. An autocorrelation consistently falling outside the confidence bands meant that the model failed to incorporate important relationships between the current output and past values of the inputs. Confidence bands were calculated with a significance level set to 0.05 [46].

## 3 Results

Training the neural network required approximately 30 min (PC equipped with 2xTitan i75900), while Model 1 and 2 calibration (particle swarm optimization) required 10 min and 20 min respectively (MacBook Pro, 2.8 GHz Intel Core i7). Testing the models required few seconds for every simulation.

**Fig 2:**
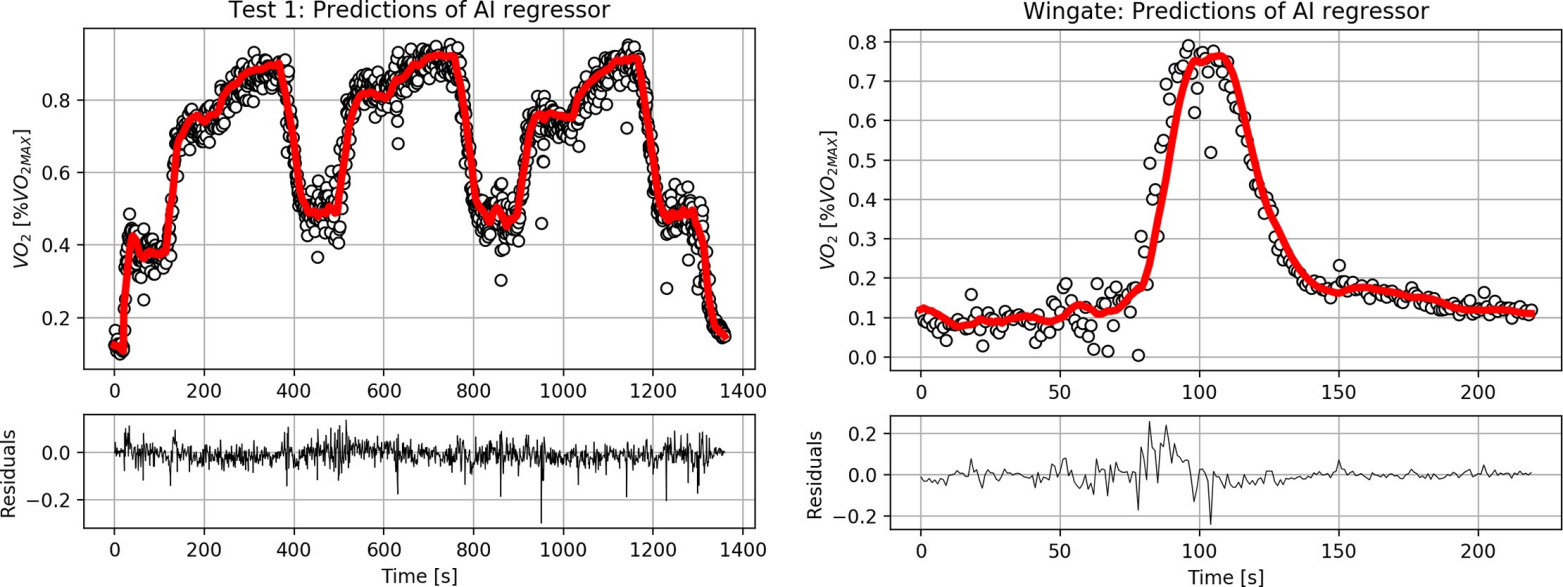
A) The performance of the regressor is shown for a single representative athlete. Experimental data (circles) of oxygen uptake ($\dot{V}O_{2}$) are reported. Predicted values of $\dot{V}O_{2}$ (solid line) are superimposed on experimental data. In this example, MAE was 0.028 $\dot{V}O_{2MAX}$ (i.e. 164 mlO_2_min^-1^), with a RMSE of 0.04 $\dot{V}O_{2MAX}$ (i.e. 229 mlO_2_min^-1^). **B)** The performance of the regressor is shown for a single representative athlete during a WG test. Experimental data (circles) of oxygen uptake ($\dot{V}O_{2}$) are reported. Predicted values of $\dot{V}O_{2}$ (solid line) are superimposed on experimental data. In this example MAE was 0.03 $\dot{V}O_{2MAX}$ (i.e. 176 mlO_2_min^-1^), with a RMSE of 0.05 $\dot{V}O_{2MAX}$ (i.e. 294 mlO_2_min^-1^). Please see the S1 Material for the other individuals’ responses.

In trial 1, after the particle swarm optimization, mean values of the parameters of Model 1 were: G = 10.07 (0.85) mlO_2_min^-1^W^-1^ and τ = 45 (3) s. Values for Model 2 are reported in the Appendix. For the neural network and Models 1 and 2, results of the residual and Bland-Altman analyses are presented collectively in **Table 3** for both the experimental Trials. The performances of the neural network in Trial 1 and 2 are shown in **Fig 2A** and **2B** for a representative participant.

**Table 3:** Results are reported for the residual and the Bland-Altman analyses for the three different models (AI reg. (i.e. our AI regressor), model 1 (i.e. the first-order model) and 2 (i.e. the Gonzalez’s model)). In Trial 1 we compared predicted and experimental data during a variable high-intensity exercise. In Trial 2 we compared predicted and experimental data during a brief 30” “all-out” Wingate test.

Trial	Model	MAE	RMSE	r	R^2^	Bias	LA_95%_	CI_95%_	Abs. range
1	AI reg.	5.3 (1.1)	7.3 (1.5)	0.94 (0.02)	0.89 (0.04)	1.7 (2)	13.4 (3.3)	1.7	66 (73)
	Model 1	7.9 (1.4)	10 (1.6)	0.83 (0.06)	0.7 (0.1)	-5 (2)	15 (2.4)	1.4	-264 (79) **
	Model 2	6.2 (1.3)	8.3 (1)	0.9 (0.04)	0.81 (0.07)	-2.7 (2.1)	15 (2)	1.6	-114 (62)*
2	AI reg.	7.2 (4.6)	11 (6.4)	0.89 (0.09)	0.8 (0.15)	-3.6 (5)	20 (9.5)	3.8	-124 (139)
	Model 1	9 (2.4)	12.7 (3.2)	0.75 (0.09)	0.58 (0.13)	-6.2 (1)	21.4 (7)	1	-277 (50)**
	Model 2	10.8 (3.7)	15 (4.3)	0.48 (0.25)	0.28 (0.21)	-8.7 (2.9)	23 (7.7)	2.1	-377 (75)**

Mean (SD) values are reported. MAE is the mean average error in $\%\dot{V}O_{2MAX}$, RMSE is the root mean square error given in $\%\dot{V}O_{2MAX}$, *r* is the correlation coefficient, *R*^*2*^ is the corresponding variance explained, the bias is given in $\%\dot{V}O_{2MAX}$, the limits of agreement LA_95%_ are given in $\%\dot{V}O_{2MAX}$, the confidence intervals CI_95%_ are given in $\%\dot{V}O_{2MAX}$, the absolute range is calculated from the individual characteristics and provided in mlO_2_min^-1^. The predicted $\dot{V}O_{2}$ values were significantly biased if the equality line fell outside the confidence intervals of the mean bias of the sample (*) or outside the limits of 200 mlO_2_min^-1^ (**).

The residual analysis in Trial 1 shows that the predictive power of the neural network was significantly superior to that of the other models, as seen by the smaller mean absolute error and root mean square error and higher correlation coefficient and variance explained (**Table 3**). For both our neural network and Models 1 and 2, the Bland-Altman analysis for the measured versus predicted $\dot{V}O_{2}$ showed no proportional error rate, with differences unrelated to the magnitude of the measurement error. In the case of the neural network the bias was not significant. For model 1, the equality line fell outside the confidence intervals of the mean bias of the sample and outside the limits of 200 mlO_2_min^-1^. Model 2 performed slightly better than Model 1: the equality line fell outside the confidence intervals of the mean bias of the sample but inside the limits of 200 mlO_2_min^-1^. For the neural network, the autocorrelation analysis suggested that there was no significant autocorrelation between observations and lagged observations. In fact, autocorrelation consistently stayed within the confidence bands. In the case of Model 1 and 2, the autocorrelation fell outside the confidence bands for the initial portion of the signal.

During Trial 2, residual analysis highlighted that the neural network could accurately predict the actual $\dot{V}O_{2}$ data both during the ascending and descending phases of the $\dot{V}O_{2}$ evolution. On the contrary, both Models 1 and 2 did not predict the additional $\dot{V}O_{2}$ required after high-intensity exercises. This is confirmed by the high values of correlation coefficient and variance explained for the predictions of the neural network (**Table 3**). Bland-Altman analysis suggested that, in the case of the regressor, the bias was not significant. On the other hand, for Model 1, the equality line fell outside the confidence intervals of the mean bias of the sample and outside the limits of 200 mlO_2_min^-1^. Model 2 performed slightly worse: the equality line fell outside the confidence intervals of the mean bias of the sample and outside the limits of 200 mlO_2_min^-1^. Bland-Altman analysis suggested that, in the case of Models 1 and 2, the biases were significant. The autocorrelation analysis for the predicted values of the neural network showed that there was no significant autocorrelation between observations and lagged observations. When predictions were made with Models 1 and 2, autocorrelation analysis highlighted that the other models failed to incorporate important relationships between current $\dot{V}O_{2}$ and past input values. In fact, a consistent portion of the autocorrelation lied outside the confidence bands.

## 4 Discussion

We hypothesized that a recurrent neural network approach could be successfully used to accurately predict individual cycling $\dot{V}O_{2}$ data from easy-to-collect inputs [21]. In fact, the mechanical power output and the pedalling cadence are both easily collectable by portable power-meters [7]). Indeed, heart rate and respiratory frequency are both measurable with chest belts [47,48] and have already been successfully used by Beltrame et. al. [49] for the prediction of $\dot{V}O_{2}$ from wearable sensors.

The ability of neural networks to model complex data was already known and other more basic learners could have been used (e.g. k-nearest-neighbour or support vector machine). While simpler learners like Hammerstein-Wiener models have been already tested [32,50], we are not aware of any existing example of the application of k-nearest-neighbour or support vector machine methods in the prediction of $\dot{V}O_{2}$ during high-intensity cycling.

However, the major innovation of our neural network lies in the long histories of values of the inputs (reflected in the number of input neurons). Latinen & Rasanen [30] used a neural network with 14 input neurons, one hidden layer of 4 neurons and one output neuron for $\dot{V}O_{2}$. Beltrame et al. [33] used a neural network with 7 input neurons, one hidden layer of 11 neurons and one output neuron. Both studies only used current inputs and not past values. Beltrame et al. [49] used only 1 sec of lag to include “dynamic changes” of heart rate. Very recently, Borror et. al. [35] presented a neural network that can predict cycling $\dot{V}O_{2}$ in cycling, with a workflow similar to ours. They included body mass, mechanical power output, pedalling cadence and heart rate as inputs. However, in their work, no past input values are passed to the neural network, and the heart rate dynamics is only considered by means of its “time derivative”. In our neural network, there were 3 hidden layers of 32 long-short term memory neurons each, one hidden layer of 10 neurons and one output neuron. The neurons adopted were long-short term memory neurons, particularly suited for time-series analysis [26]. To the best of our knowledge, we are the first to apply recurrent neural networks to the prediction of cycling $\dot{V}O_{2}$.

The predictive power of the neural network was very high during Trial 1, as measured and predicted $\dot{V}O_{2}$ showed a nearly perfect agreement (MAE = 229 (35) mlO_2_min^-1^, *r* = 0.94). The performances of models 1 and 2, although inferior, were still good in Trial 1 (MAE = 355 (86) mlO_2_min^-1^, *r* = 0.83 and 273 (49) mlO_2_min^-1^, *r* = 0.9 for model 1 and 2 respectively). This means that the performances of our neural network and Models 1 and 2 were very close. However, a robust model should predict $\dot{V}O_{2}$ data across a wide range of scenarios. To this end, we tested our models using a short “all-out” sprint effort (Wingate test). Importantly, Models 1 and 2 were not designed specifically for the Wingate test and have a limited number of parameters that can be tuned. However, the neural network, due to the considerable number of parameters used, can work well across a wider range of exercising scenarios. In fact, the number of parameters used may limit the number of physiological mechanisms that can be mathematically described.

In Model 1, a single phase is included and characterised by the parameter τ. The time-constant τ and the oxygen gain G, in Model 1, are constant across all the exercising intensities. Therefore, it becomes difficult to predict experimental values of $\dot{V}O_{2}$ during brief “all-out” exercises [51]. In Model 2, the parameter T_I_ (see Appendix) has been included to account for the delayed $\dot{V}O_{2}$ component that sum to the principal element. However, if the time duration of the exercise is shorter than T_I_ (e.g. the Wingate test lasts 30”), then this additional component is not activated. In Model 2, the high number of parameters affected the confidence of parameter estimation and this is confirmed by the large variability of the parameter estimates. Mean and standard deviation of the variables found with our experimental data, were compatible to those reported in the original article [31] (Appendix).

On one hand, the high predictive power of the neural network, although reduced, was remarkably conserved during Wingate test (Trial 2: MAE = 304(150) mlO_2_min^-1^, *r* = 0.89). This means that the dataset that we used to train the neural network (in terms of duration of the exercises) for every participant, was large enough to provide good predictive power. Further studies are needed to establish the minimal amount of data that should be used to train a neural network and retain a high predictive ability. On the other hand, the performances of Models 1 and 2 deteriorated during Wingate test (Trial 2: MAE = 391(71) mlO_2_min^-1^, *r* = 0.75 and MAE = 463(112) mlO_2_min^-1^, *r* = 0.48 for the Model 1 and 2 respectively). It can be noticed (**Fig 2B**) that a small lag is present between the $\dot{V}O_{2}$ measurements and predictions. This might be because two of the inputs used by the neural network (i.e. respiratory frequency and heart rate) did not promptly react to abrupt changes in power output. However, an autocorrelation analysis showed that our neural network could incorporate relevant relationships between current $\dot{V}O_{2}$ and past input values, whereas Models 1 and 2 could not. Due to the reduced number of parameters of Model 1 and 2, the predictive power does not heavily depend on the amount of data used to calibrate the parameters. We suggest that the performance of the analytical models, although inferior, is guaranteed even if smaller datasets are used for their calibration. We did not investigate the influence of the dimension of the training set on the performance of the neural network, but we believe that the performance would deteriorate with smaller and smaller training datasets. This is a first limitation of a neural network approach: we rely on large datasets.

The second main limitation of the neural network method lies in its “black box” approach. In fact, it is unlikely we can understand how the non-linearities of the $\dot{V}O_{2}$ dynamics are represented inside the neural network. Additionally, our exercises were carried out in the laboratory environment and they were limited in time (max duration ~1400 s). In practical settings (e.g. training and races), a cardiovascular drift could mislead our estimations. The use of long-short term memory neurons makes it difficult to understand the variables that contribute the most to the total estimation. In our study, the pedalling cadence was kept constant, so it is likely that the contribution of this variable may be limited in our study. As well, respiratory frequency indicating the ventilatory response to exercise has an important link with $\dot{V}O_{2}$, while heart rate has additional known associations with exercising $\dot{V}O_{2}$ [5,52].

Even though we investigated a few different exercising conditions (i.e. moderate, heavy and severe intensity and “all-out” efforts), we should the results of this pilot study with caution. In fact, more work is needed before this algorithm could be embedded in a portable system able to estimate cycling $\dot{V}O_{2}$ in real-time: the verification of the predictive ability of the neural network on a larger sample (7 cyclists is a small sample) and on different environmental conditions (e.g. outdoor). Also, including input parameters like body mass, gender and fitness level, may provide in the future even better predictive outcomes for the estimation of the aerobic performance. Importantly, the ability of the neural network in predicting the $\dot{V}O_{2}$ values for an individual who was not included in the training dataset, has yet to be assessed.

## 5 Conclusions

In the context of forecasting $\dot{V}O_{2}$ values, the results of our pilot study suggested that a recurrent neural network can use great quantities of information from other mechanical (such as mechanical power output and pedalling cadence) and physiological markers (such as heart rate and respiratory frequency), as well as past input values, to attain accurate predictions of cycling $\dot{V}O_{2}$. Results suggest that this algorithm has the potential to be, in the next future, embedded in a portable system and to provide real-time assessment of individual cycling $\dot{V}O_{2}$ during training or racing.

## Appendix

Dynamics equations of Model 2 (see [31,53]) are reported with a formulation that can be readily implemented in a spreadsheet. The main difference between this model and Model 1 is that phase-II (“fast” phase) and III (“slow” phase) of $\dot{V}O_{2}$ dynamics are considered in this model. Gonzalez et al. included these two additional phases with two delayed components that become active only after a given period. The principal governing equation is:

$${\dot{V}O}_{2}(k+1)={\dot{V}O}_{2R}+{\dot{V}O}_{2\;II}(k+1)+{\dot{V}O}_{2\;III}(k+1)$$

Where $\dot{V}O_{2II}$ is the principal $\dot{V}O_{2}$ component that is active after T_II_ and is characterized by a time-constant τ_II_.

$${\dot{V}O}_{2\;II}(k+1)=\frac{{{(A}_{II}(k)-\dot{V}O}_{2\;II}(k))\Delta t}{\tau_{II}}+{\dot{V}O}_{2\;II}(k)$$

Where A_II_(k) can be computed as:

$$A_{II}(k)=min(s∙P(k),{\dot{V}O}_{2MAX}-{\dot{V}O}_{2R})$$

Where s is the gain for the fast phase. $\dot{V}O_{2III}$ is the slow component of $\dot{V}O_{2}$ that activates after T_III_ and is characterized by a time constant τ_III_.

$${\dot{V}O}_{2\;III}(k+1)=\frac{{(A}_{III}(k)-{\dot{V}O}_{2\;III}(k))\Delta t}{\tau_{III}}+{\dot{V}O}_{2\;III}(k)$$

Where A_III_(k) can be computed as:

$$A_{III}(k)=\left\{ \begin{array}{r} {{\dot{V}}_{\Delta }∙e}^{\left(-(Pc-P(k))/\Delta \right)},\;P(k)\leq Pc\\ {{\dot{V}O}_{2MAX}-\dot{V}O}_{2R}-A_{II}(k),\;P(k)>Pc \end{array}\right.$$

Where Pc is a “critical power” threshold.

In trial 1, after PSO, the values of the parameters were (mean(SD)): $\dot{V}\Delta$ = 397(398) mlO_2_min^-1^, Pc = 359(39) W, Δ = 79(72) W, s = 8.67(0.49) mlO_2_min^-1^W^-1^, τ_I_ = 43(1.38) s, τ_II_ = 199(52) s, T_I_ = 10(6.72) s, T_II_ = 113(27) s. In trial 2, after PSO, the values of the parameters were: $\dot{V}\Delta$ = 779(445) mlO_2_min^-1^, Pc = 383(15) W, Δ = 64(34) W, s = 9.03(0.9) mlO_2_min^-1^W^-1^, τ_I_ = 42(1.7) s, τ_II_ = 183(30) s, T_I_ = 11(4.5) s, T_II_ = 103(34) s.

## Supporting information

S1 Material (PDF)
