# Supplementary material for: Estimating an individual’s oxygen uptake during cycling exercise with a recurrent neural network trained from easy-to-obtain inputs: A pilot study
Source: PLoS One. 2020 Mar 12;15(3):e0229466. doi: 10.1371/journal.pone.0229466 (PMC7069417; doi:10.1371/journal.pone.0229466)
Supplement: S1 Material — (PDF) [file pone.0229466.s001.pdf]

Estimating an individual's oxygen uptake during cycling exercise with a recurrent neural network trained from easy-to-obtain inputs: a pilot study

Additional material

A. Zignoli[*], A. Fornasiero, M. Ragni, B. Pellegrini, F. Schena, F. Biral, P.B. Laursen

February 6, 2020

Introduction

We aimed at developing a recurrent neural network with the ability to predict dynamic oxygen uptake ($\dot{V}O_2$) responses. The $\dot{V}O_2$ prediction model included inputs of heart rate (HR), power output (P), respiratory frequency (RF) and pedalling cadence (ω). The neural network was trained with inputs collected on 7 participants during a long-graded incremental-to-exhaustion test, a Wingate test and two protocols comprising step-changes in exercise intensity domain (Protocol 1 and 2). We used a recurrent neural network as a regressor. Results were tested by comparing the measured $\dot{V}O_2$ response from: 1) Test 1 with a model informed by the long-graded incremental-to-exhaustion test, Wingate and Test 2 data (Trial 1) and; 2) the Wingate test with a model informed by the long-graded incremental-to-exhaustion test and Tests 1 and 2 data (Trial 2).

Info: This Additional Material document comes together with the main manuscript. Please refer to the main manuscript for the details about the methods. To provide additional transparency, results of the fitting procedures are also provided as *.txt* files.

Notice: This additional document comes together with a project repository, where researchers can clone or download scripts and data. The link will point to a branch that will not be modified after the submission. However the main code will be updated in the master branch. At the bottom of this document we briefly introduce how to use this framework to train and test custom-made neural networks. The code can be downloaded, modified and replicated in blocks or entirely.

[*]Mail to: andrea.zignoli@unitn.it

1 Content

Relevant output figures are reported here from the application of the neural network model and of the analytical model (model 1 and 2). Personal, anthropometric and physiological data of the participants are also provided.

1 Neural network model

(a) Residual plot comparing the predicted and actual $\dot{V}O_2$ response for every subject ($\%\dot{V}O_{2MAX}$).

(b) Correlation plot between predicted and actual $\dot{V}O_2$ data ($\%\dot{V}O_{2MAX}$).

(c) Bland-Altman plot with mean bias VS residuals ($\%\dot{V}O_{2MAX}$).

(d) Autocorrelation plot and confidence intervals.

(e) Training set for the specific subject.

(f) Test set for the specific subject.

2 Analytical models (1 and 2)

(a) Residual plot comparing the predicted and actual $\dot{V}O_2$ response for every subject (absolute $\dot{V}O_2$) for Trial 1 and 2, where Protocol I and Wingate tests were used to validate the models, respectively.

N	Gender	age	weight	height	$\dot{V}O_{2MAX}$	PPO	P1	P2	P3
	M/F	yy	kg	cm	mlO_2min^{-1}	W	W	W	W
1	M	22.1	67.9	185	4650	360	134	286	333
2	M	25.6	79.2	182.4	5892	420	144	322	388
3	M	42.8	81.9	173.8	4320	318	104	234	287
4	F	21.6	66.2	165.9	3693	300	96	219	273
5	M	46.6	81.1	181	4120	300	96	216	270
6	M	28.8	74.4	171	4520	347	108	243	308
7	M	41.2	81.1	180.5	3912	300	85	206	271
Mean		32.69	75.97	177.09	4569	340.8	111.8	251.1	309.4
Std		10.56	6.60	6.96	700.6	45.9	22.7	44.3	45.1

Table 1: Data for every participant is provided to integrate the summary provided in the main manuscript and the Excel spreadsheet attached as additional material.

2 Sample data

Detailed information regarding the composition of our sample is provided in tab. 2.

3 Individual responses

Individual responses are provided in this section. Given that the plots are automatically generated, information regarding the number of the participant and trial1/2 settings are provided in the title and in text boxes.

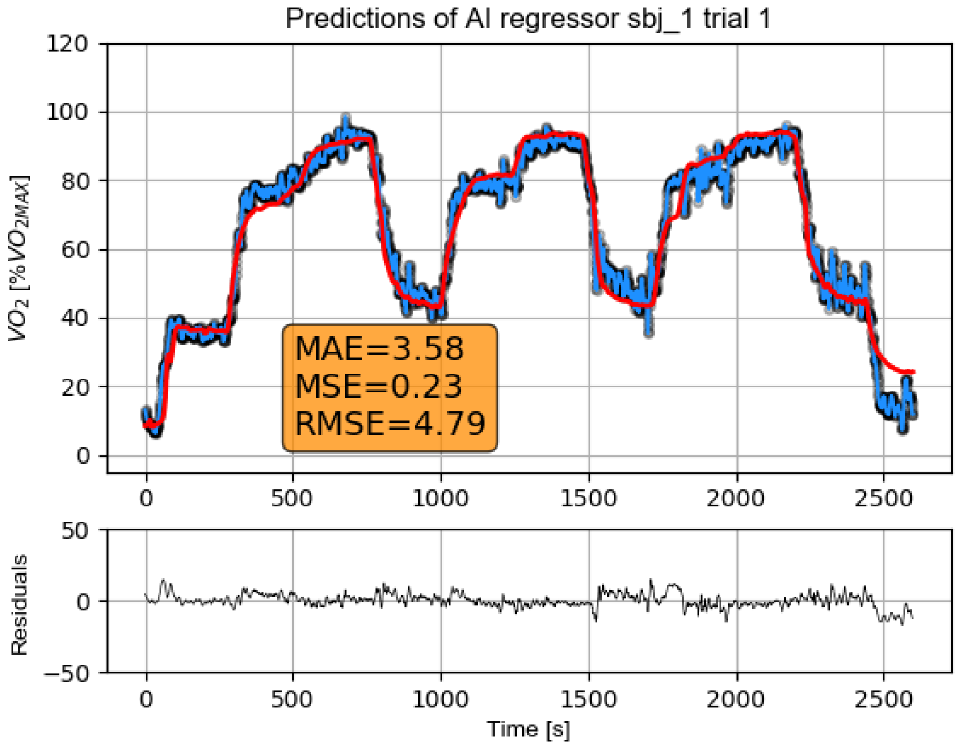

Figure 1: Oxygen uptake ($\dot{V}O_2$) experimental values (black semi-transparent dots and blue line) are compared to the estimated (red line) $\dot{V}O_2$ values. Residuals are computed as the difference between actual and estimated values. $\dot{V}O_2$ values are expressed as % of the individual's maximal $\dot{V}O_2$ (i.e. $\dot{V}O_{2MAX}$).

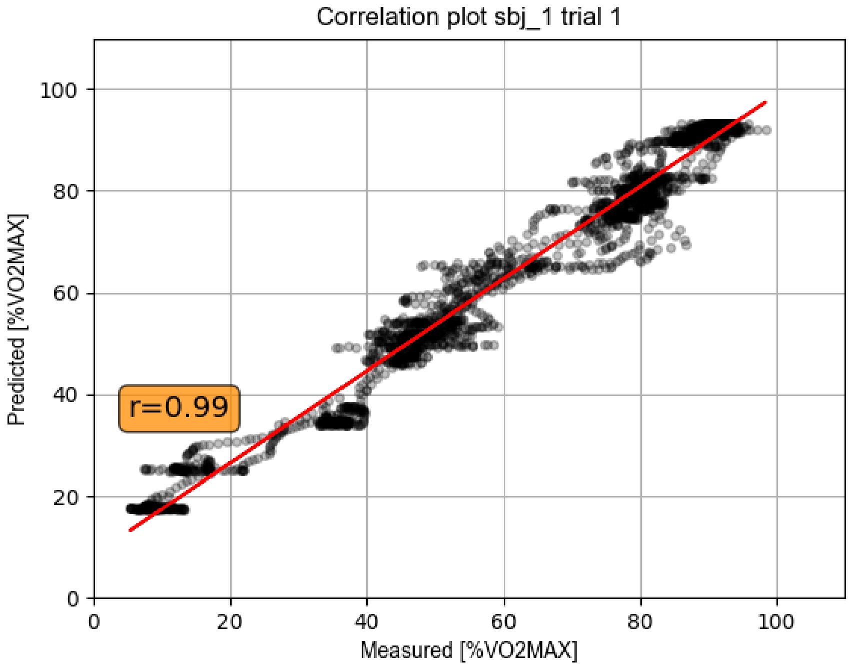

Figure 2: A scatterplot (semi-transparent black dots) that shows the relationship between actual and predicted $\dot{V}O_2$ responses. The red line represents the best-fit linear equation.

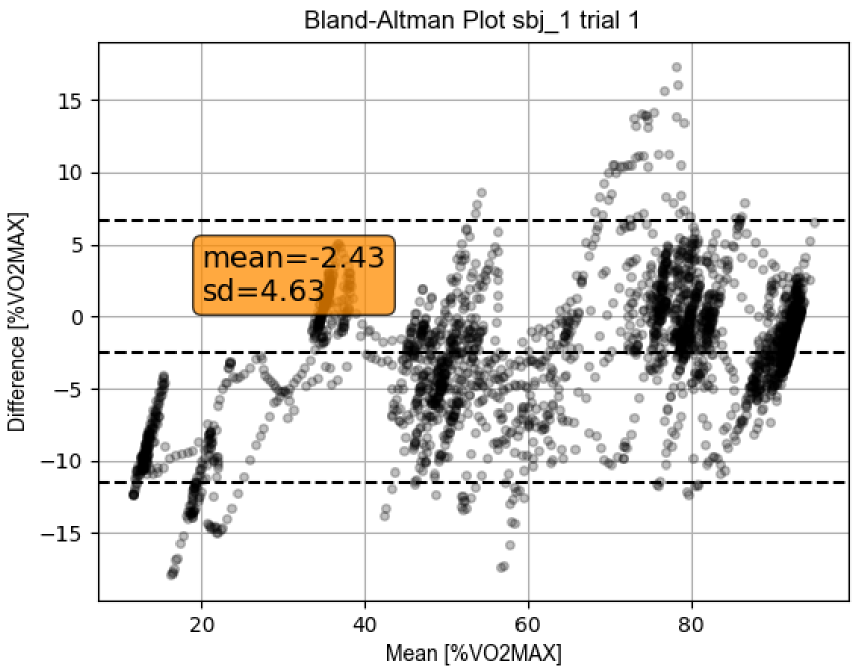

Figure 3: Bland-Altman plot is used to visually appreciate the agreement between actual and predicted $\dot{V}O_2$ responses.

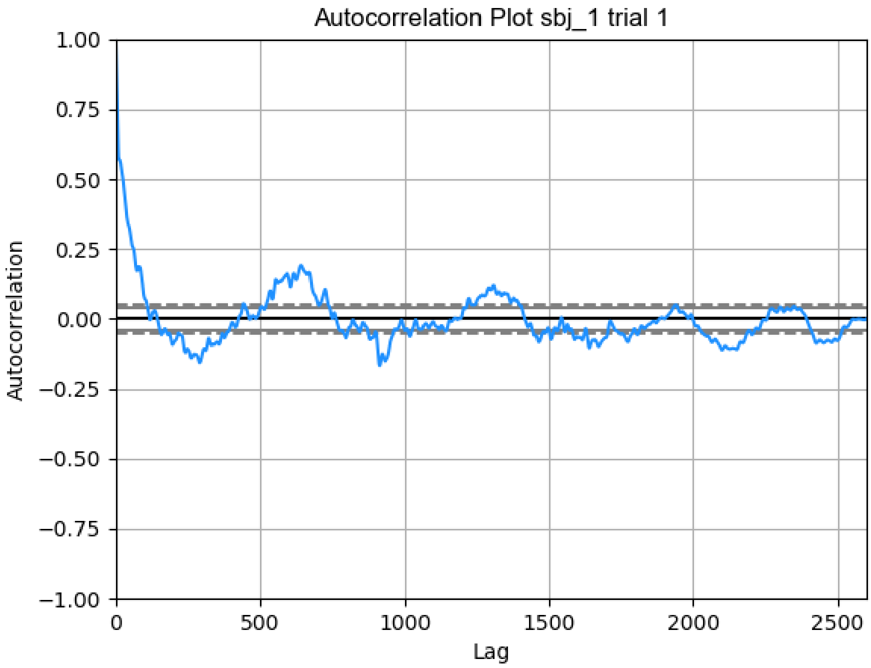

Figure 4: The autocorrelation plot (correlogram) is used to assess the correlation between the signal and the delayed (lagged) replication. Sample autocorrelation (blue line) is reported versus time lags. The horizontal (grey) lines displayed in the plot correspond to 95% and 99% (dashed) confidence bands.

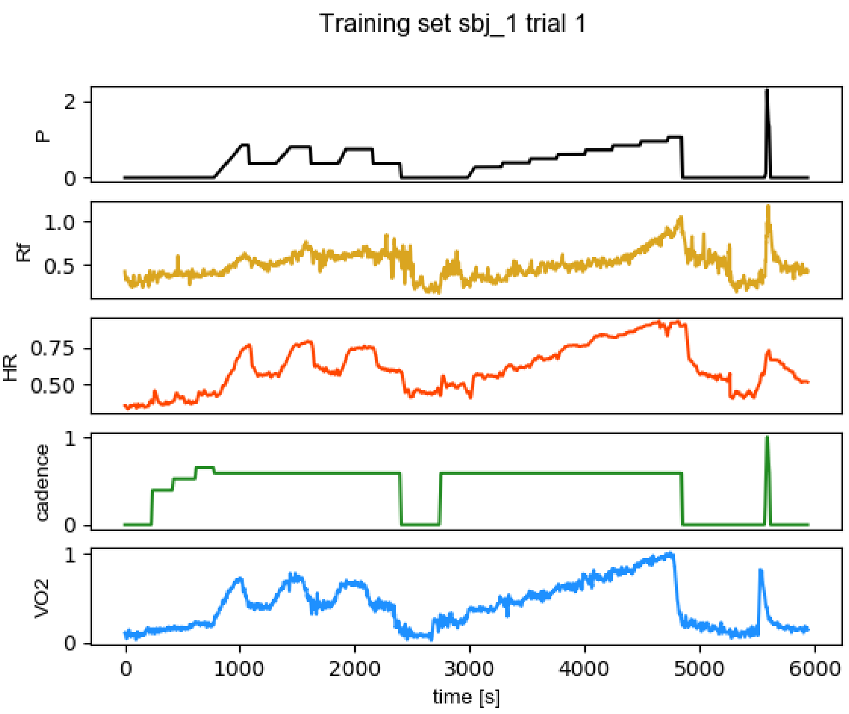

Figure 5: Training set include: power output (P, black), respiratory frequency (Rf, gold), heart rate (HR, red), cadence (ω, green) and $\dot{V}O_2$ (VO2, blue). Every quantity is normalised on the maximal value reported in the individual $.json$ file.

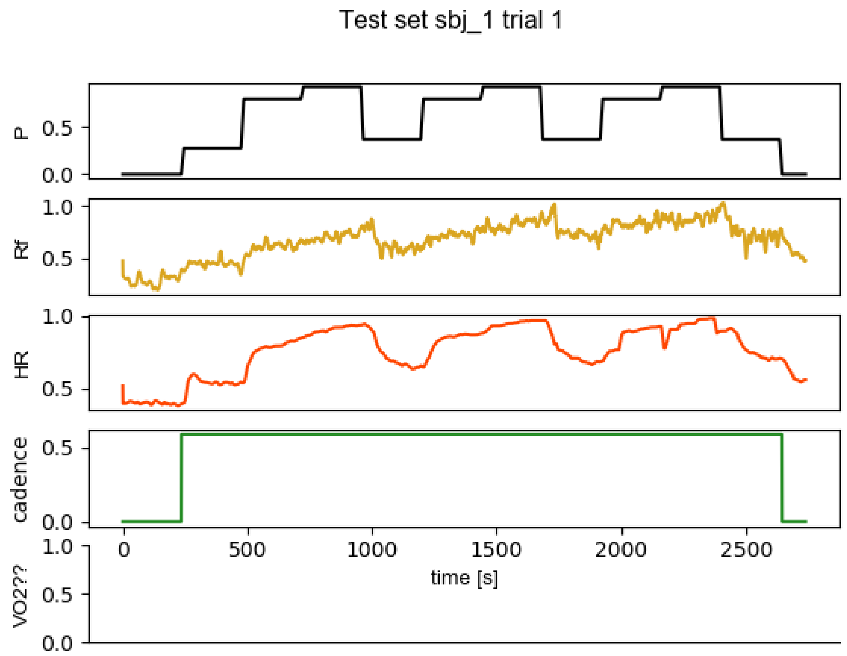

Figure 6: Test set include: power output (P, black), respiratory frequency (Rf, gold), heart rate (HR, red) and cadence (ω, green). The $\dot{V}O_2$ value is assumed to be unknown during the test set, therefore is left undefined in this plot. Every quantity is normalised on the maximal value reported in the individual $.json$ file.

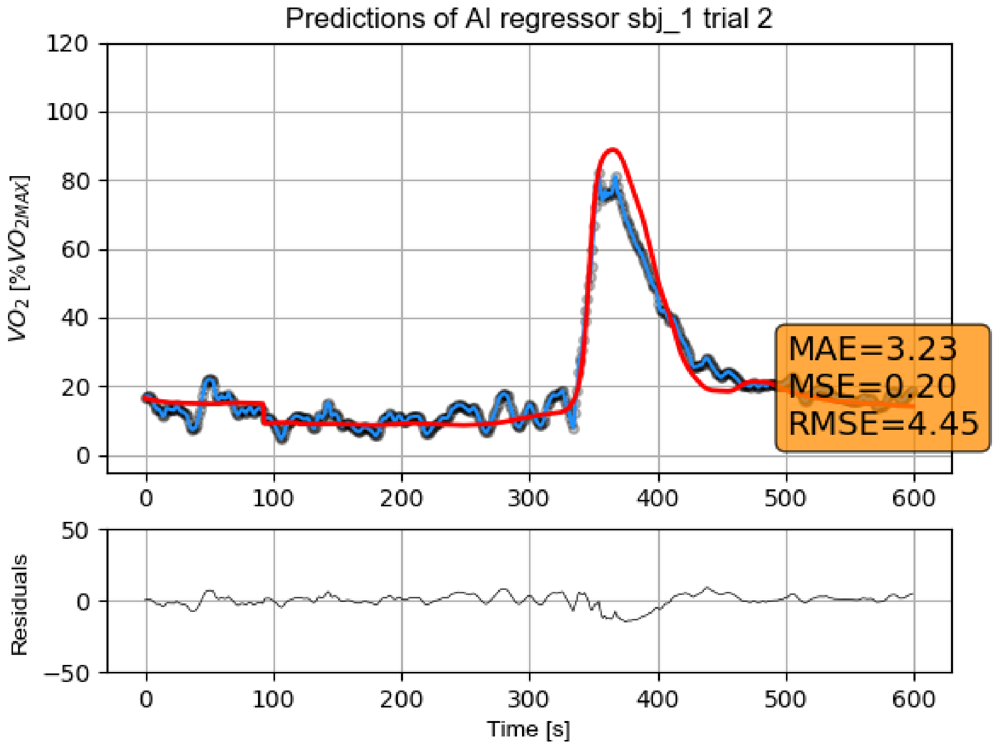

Figure 7: Oxygen uptake ($\dot{V}O_2$) experimental values (black semi-transparent dots and blue line) are compared to the estimated (red line) $\dot{V}O_2$ values. Residuals are computed as the difference between actual and estimated values. $\dot{V}O_2$ values are expressed as $\%$ of the individual's maximal $\dot{V}O_2$ (i.e. $\dot{V}O_{2MAX}$).

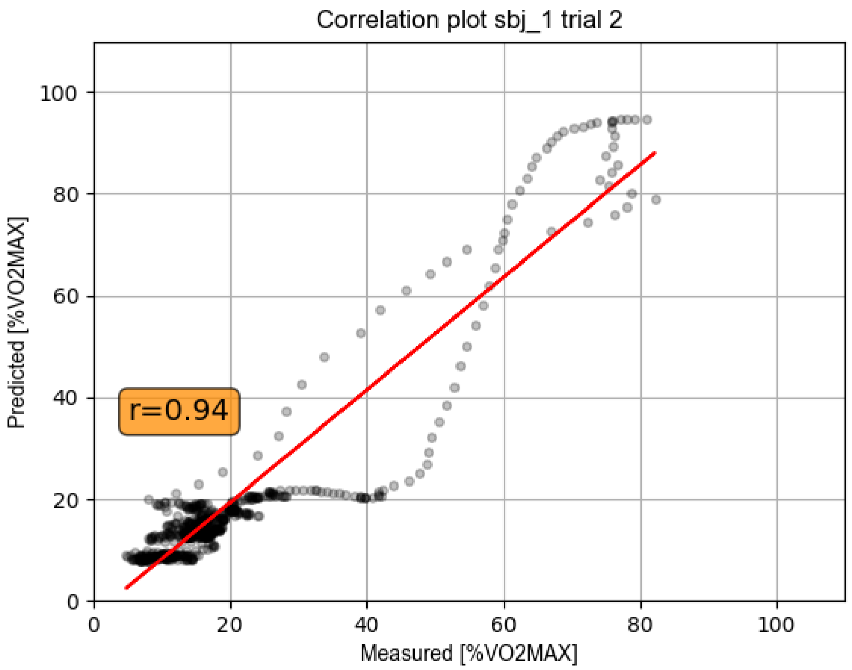

Figure 8: A scatterplot (semi-transparent black dots) that shows the relationship between actual and predicted $\dot{V}O_2$ responses. The red line represents the best-fit linear equation.

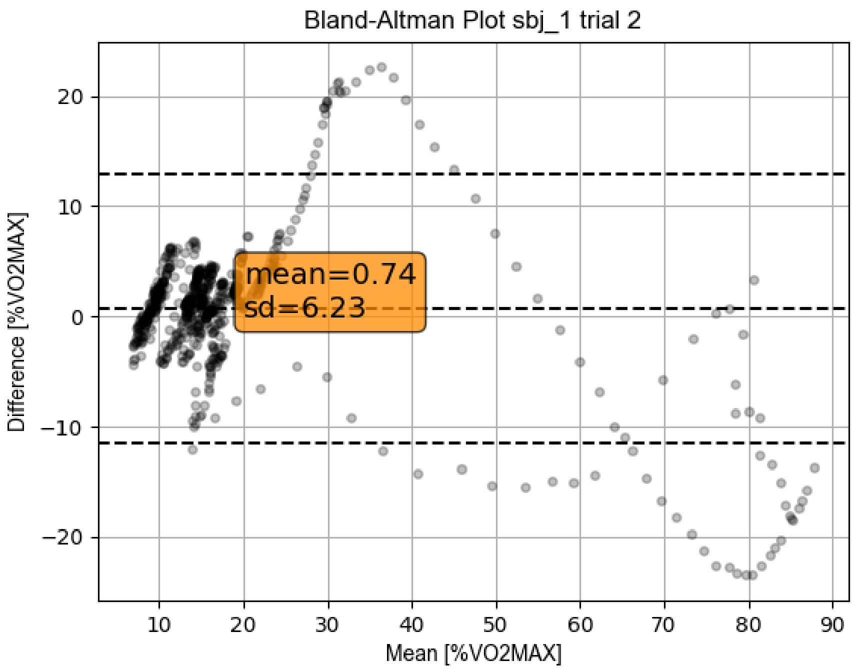

Figure 9: Bland-Altman plot is used to visually appreciate the agreement between actual and predicted $\dot{V}O_2$ responses.

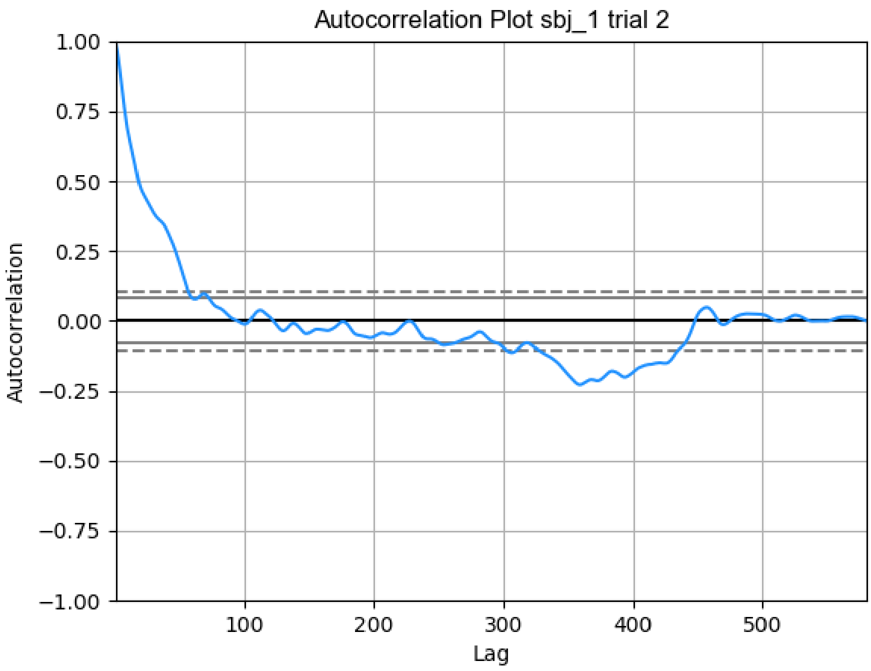

Figure 10: The autocorrelation plot (correlogram) is used to assess the correlation between the signal and the delayed (lagged) replication. Sample autocorrelation (blue line) is reported versus time lags. The horizontal (grey) lines displayed in the plot correspond to 95% and 99% (dashed) confidence bands.

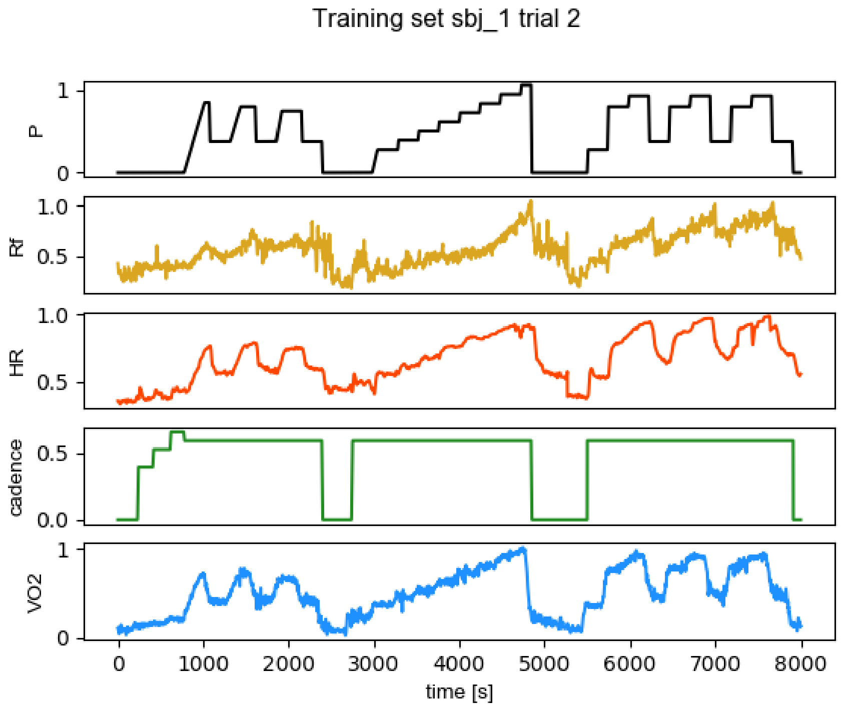

Figure 11: Training set include: power output (P, black), respiratory frequency (Rf, gold), heart rate (HR, red), cadence (ω, green) and $\dot{V}O_2$ (VO2, blue). Every quantity is normalised on the maximal value reported in the individual $.json$ file.

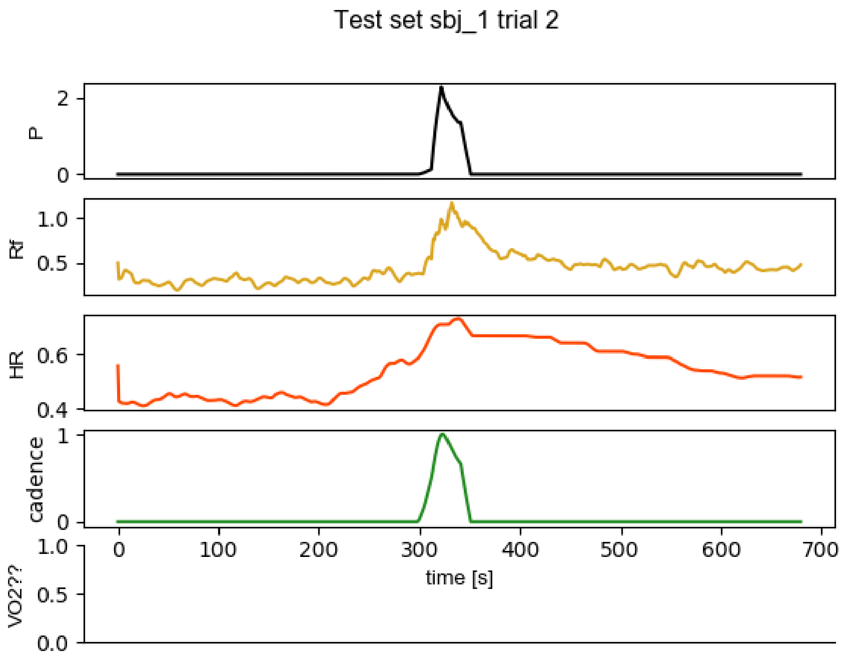

Figure 12: Test set include: power output (P, black), respiratory frequency (Rf, gold), heart rate (HR, red) and cadence (ω, green). The $\dot{V}O_2$ value is assumed to be unknown during the test set, therefore is left undefined in this plot. Every quantity is normalised on the maximal value reported in the individual $.json$ file.

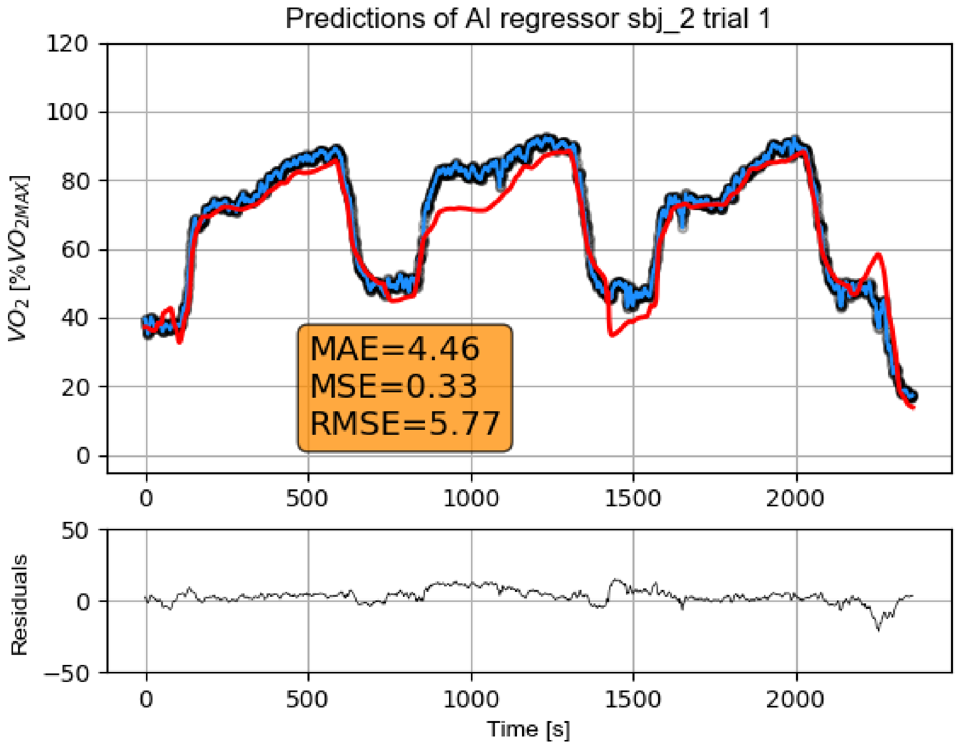

Figure 13: Oxygen uptake ($\dot{V}O_2$) experimental values (black semi-transparent dots and blue line) are compared to the estimated (red line) $\dot{V}O_2$ values. Residuals are computed as the difference between actual and estimated values. $\dot{V}O_2$ values are expressed as % of the individual's maximal $\dot{V}O_2$ (i.e. $\dot{V}O_{2MAX}$).

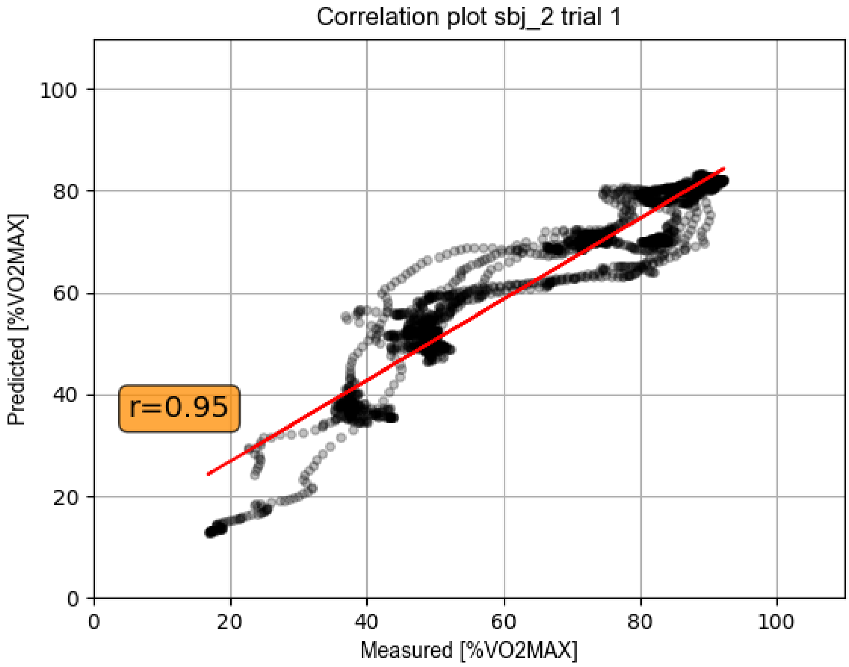

Figure 14: A scatterplot (semi-transparent black dots) that shows the relationship between actual and predicted $\dot{V}O_2$ responses. The red line represents the best-fit linear equation.

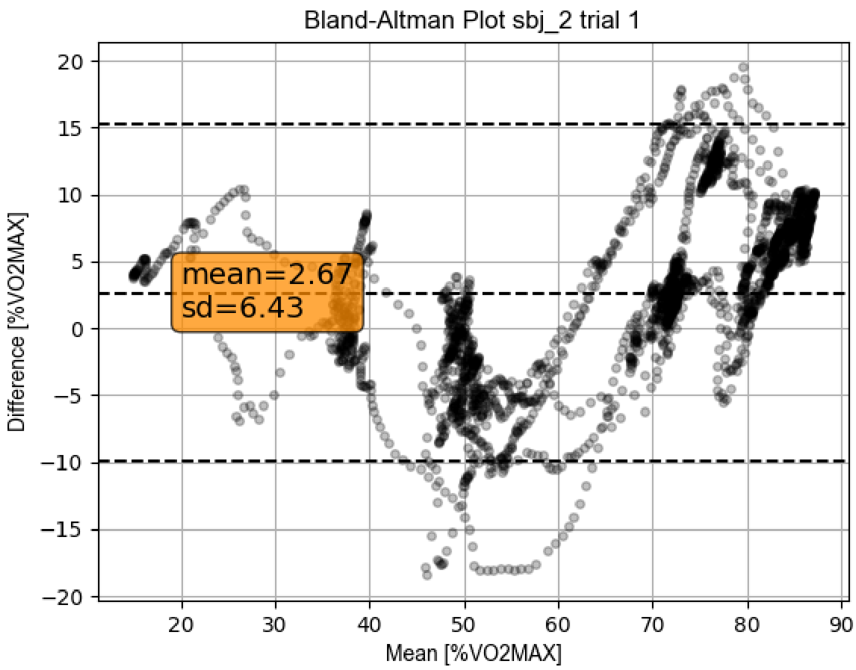

Figure 15: Bland-Altman plot is used to visually appreciate the agreement between actual and predicted $\dot{V}O_2$ responses.

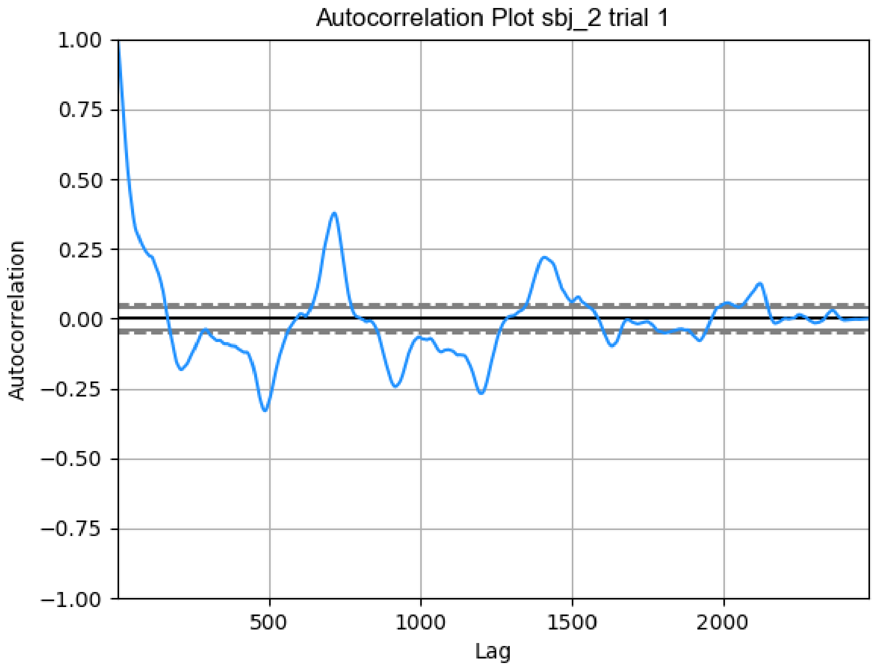

Figure 16: The autocorrelation plot (correlogram) is used to assess the correlation between the signal and the delayed (lagged) replication. Sample autocorrelation (blue line) is reported versus time lags. The horizontal (grey) lines displayed in the plot correspond to 95% and 99% (dashed) confidence bands.

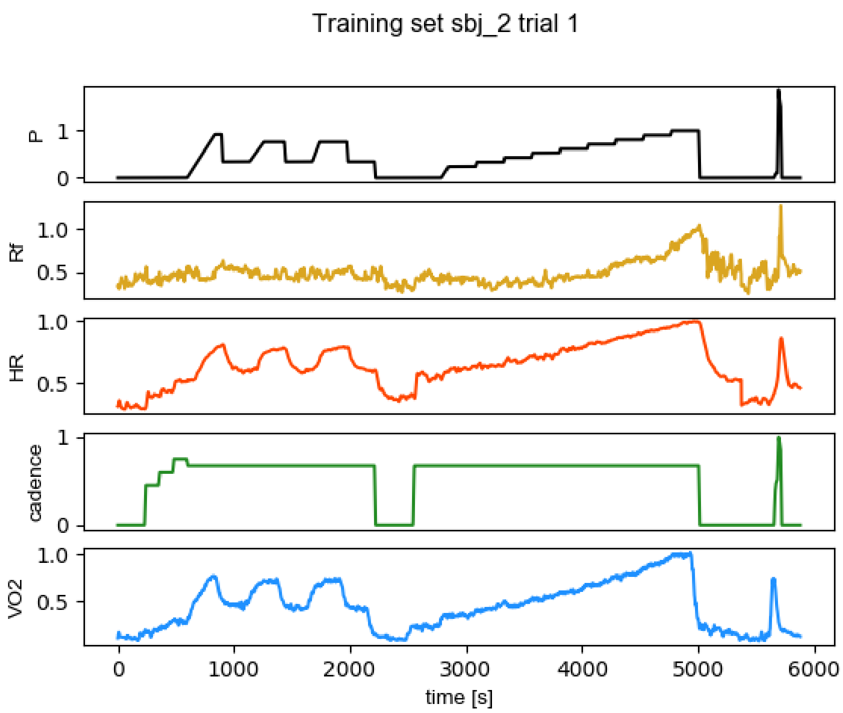

Figure 17: Training set include: power output (P, black), respiratory frequency (Rf, gold), heart rate (HR, red), cadence (ω, green) and $\dot{V}O_2$ (VO2, blue). Every quantity is normalised on the maximal value reported in the individual $.json$ file.

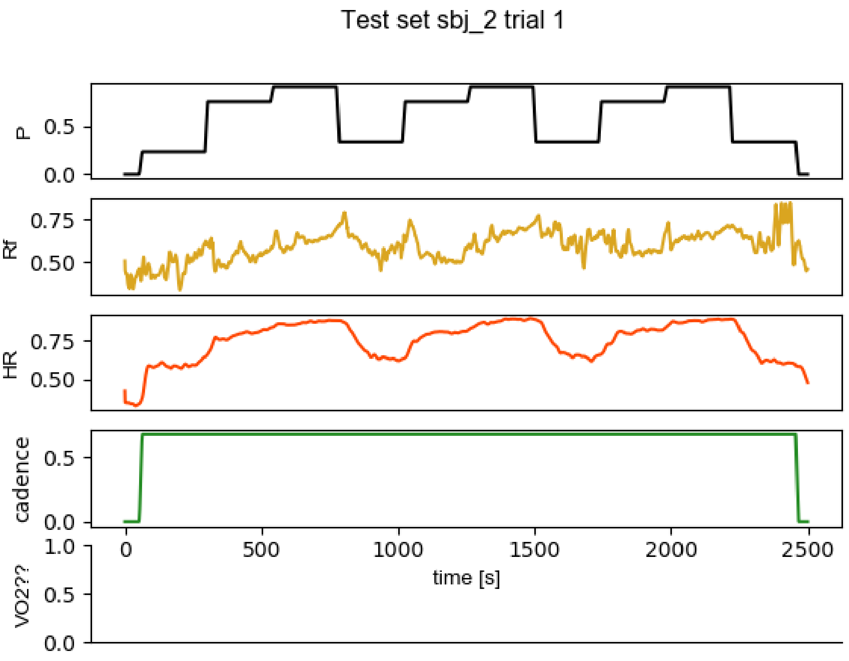

Figure 18: Test set include: power output (P, black), respiratory frequency (Rf, gold), heart rate (HR, red) and cadence (ω, green). The $\dot{V}O_2$ value is assumed to be unknown during the test set, therefore is left undefined in this plot. Every quantity is normalised on the maximal value reported in the individual $.json$ file.

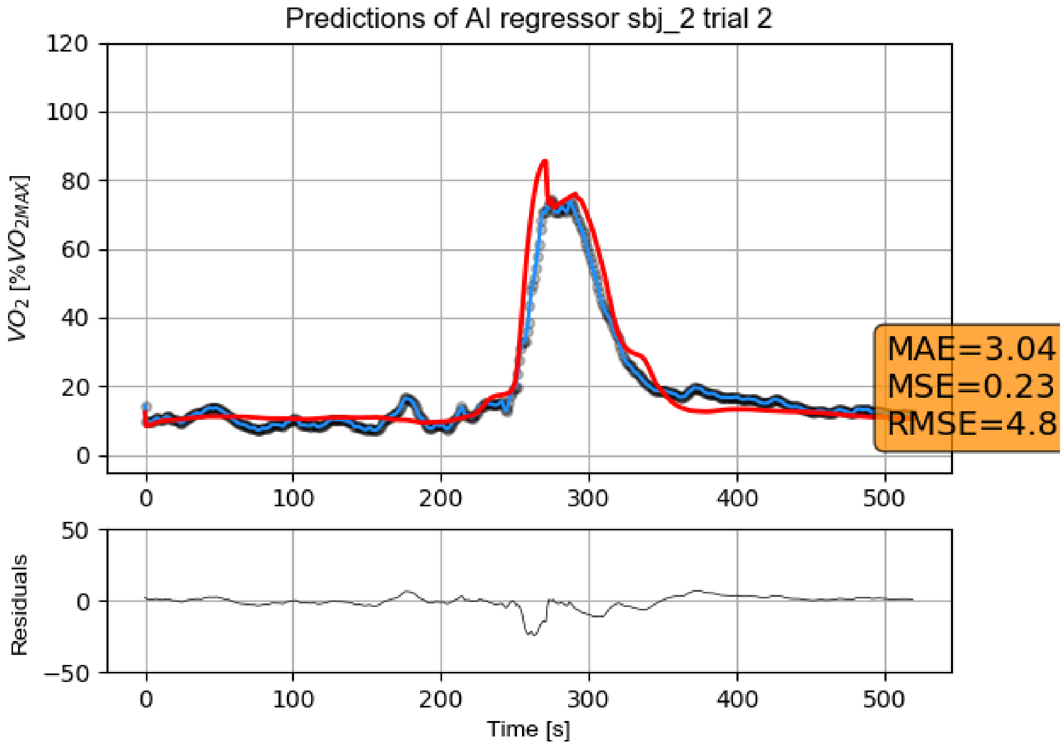

Figure 19: Oxygen uptake ($\dot{V}O_2$) experimental values (black semi-transparent dots and blue line) are compared to the estimated (red line) $\dot{V}O_2$ values. Residuals are computed as the difference between actual and estimated values. $\dot{V}O_2$ values are expressed as % of the individual's maximal $\dot{V}O_2$ (i.e. $\dot{V}O_{2MAX}$).

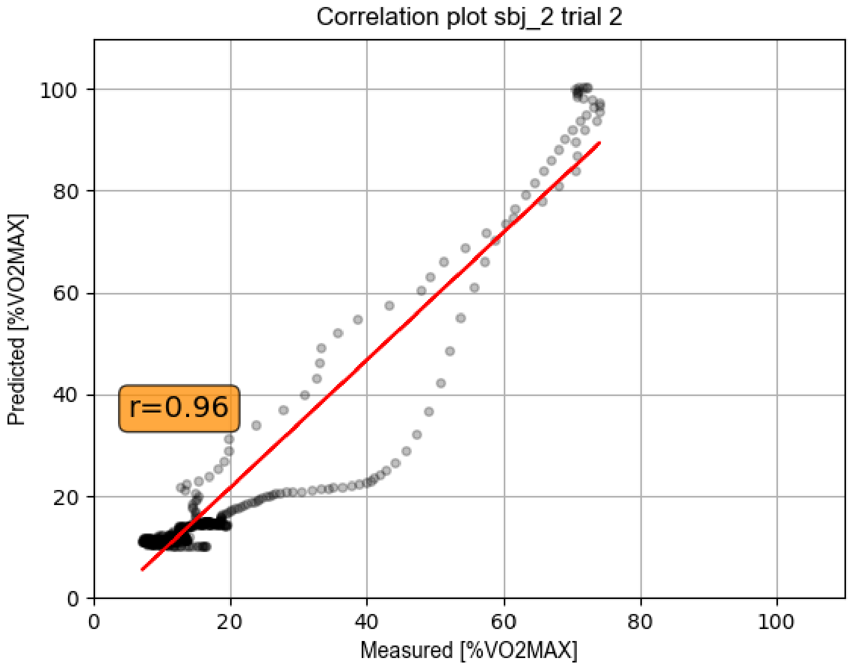

Figure 20: A scatterplot (semi-transparent black dots) that shows the relationship between actual and predicted $\dot{V}O_2$ responses. The red line represents the best-fit linear equation.

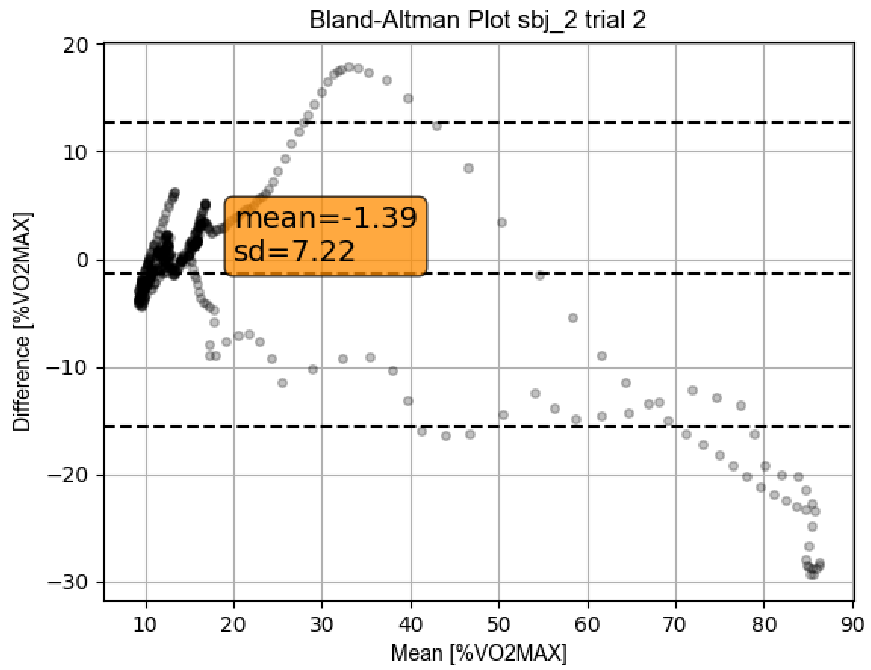

Figure 21: Bland-Altman plot is used to visually appreciate the agreement between actual and predicted $\dot{V}O_2$ responses.

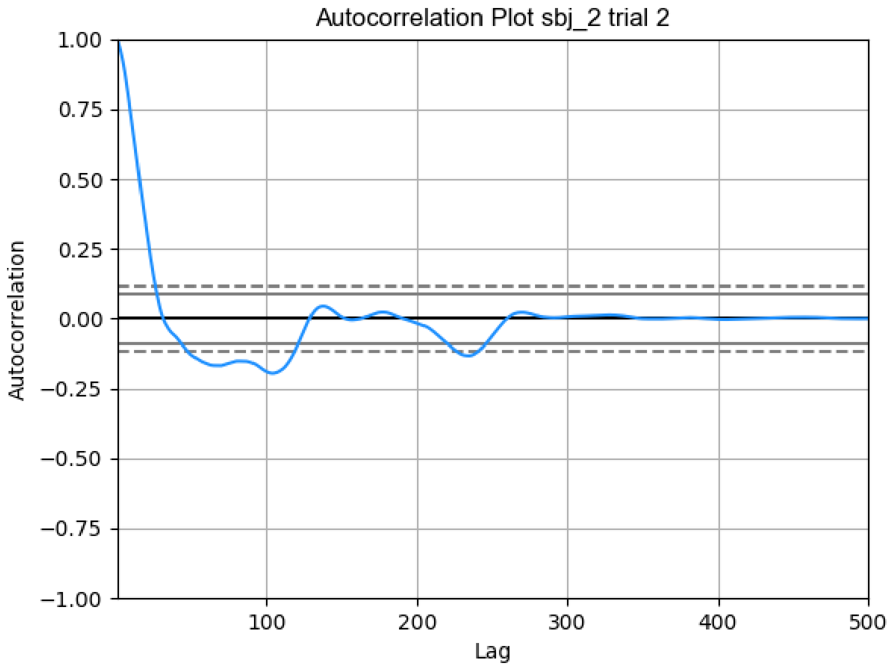

Figure 22: The autocorrelation plot (correlogram) is used to assess the correlation between the signal and the delayed (lagged) replication. Sample autocorrelation (blue line) is reported versus time lags. The horizontal (grey) lines displayed in the plot correspond to 95% and 99% (dashed) confidence bands.

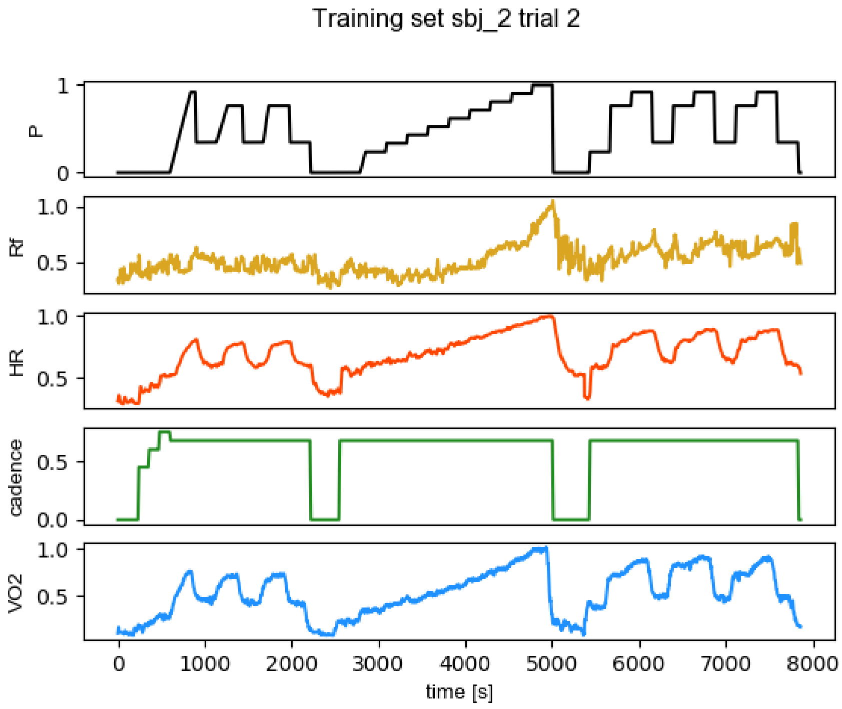

Figure 23: Training set include: power output (P, black), respiratory frequency (Rf, gold), heart rate (HR, red), cadence (ω, green) and $\dot{V}O_2$ (VO2, blue). Every quantity is normalised on the maximal value reported in the individual $.json$ file.

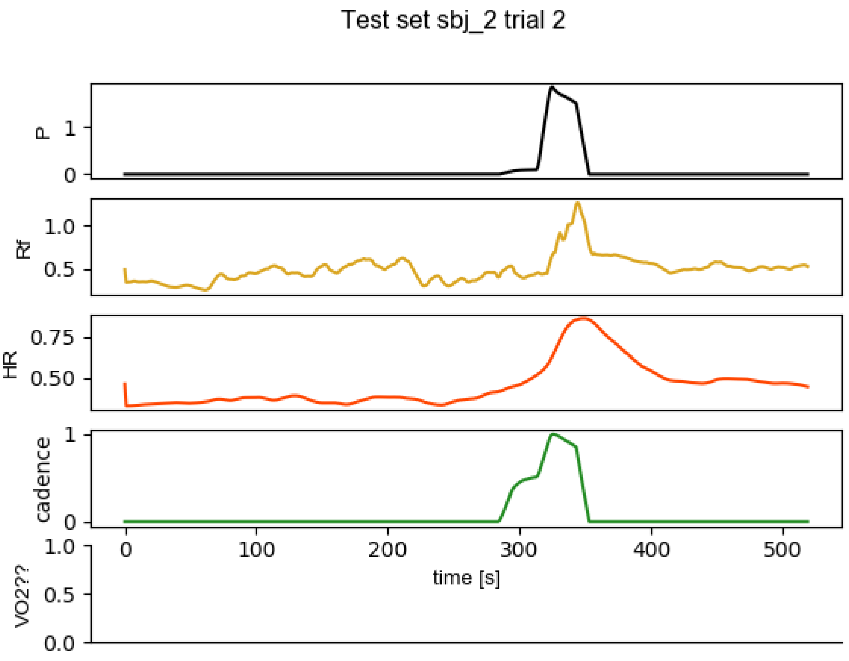

Figure 24: Test set include: power output (P, black), respiratory frequency (Rf, gold), heart rate (HR, red) and cadence (ω, green). The $\dot{V}O_2$ value is assumed to be unknown during the test set, therefore is left undefined in this plot. Every quantity is normalised on the maximal value reported in the individual $.json$ file.

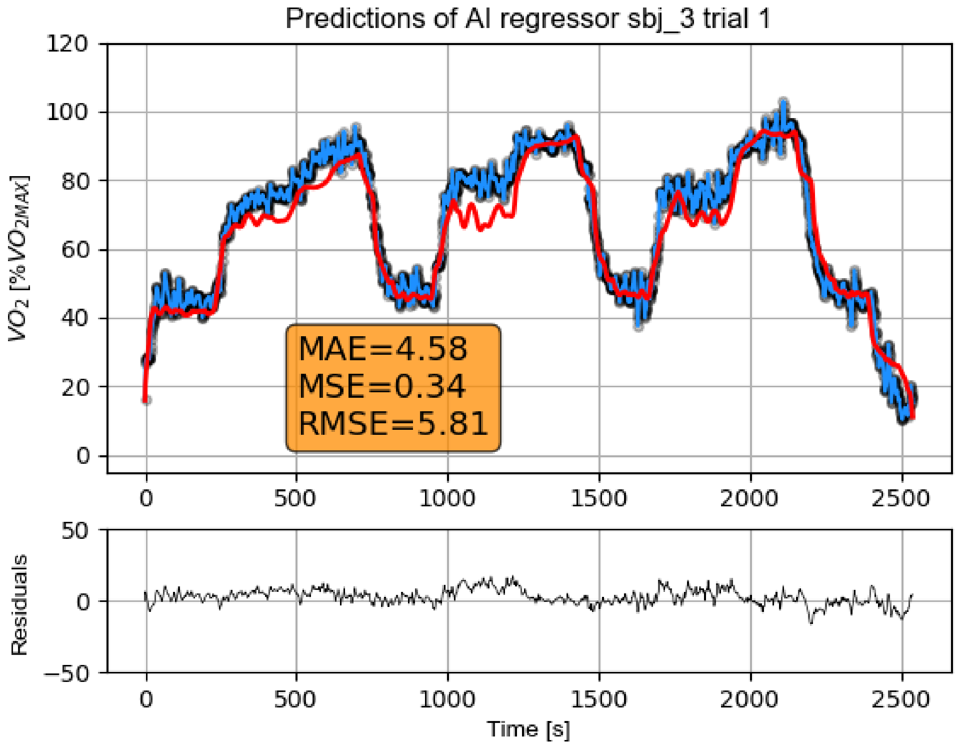

Figure 25: Oxygen uptake ($\dot{V}O_2$) experimental values (black semi-transparent dots and blue line) are compared to the estimated (red line) $\dot{V}O_2$ values. Residuals are computed as the difference between actual and estimated values. $\dot{V}O_2$ values are expressed as % of the individual's maximal $\dot{V}O_2$ (i.e. $\dot{V}O_{2MAX}$).

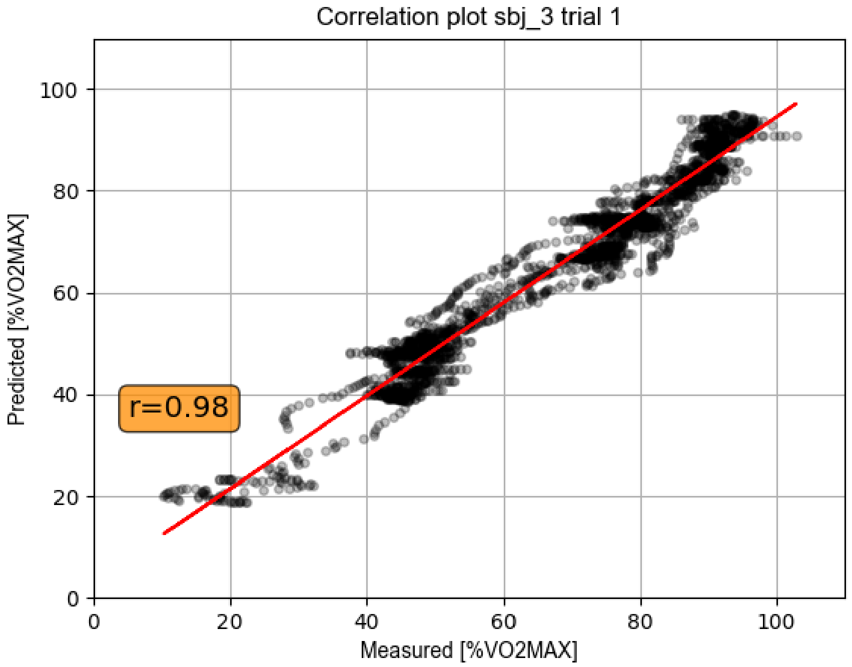

Figure 26: A scatterplot (semi-transparent black dots) that shows the relationship between actual and predicted $\dot{V}O_2$ responses. The red line represents the best-fit linear equation.

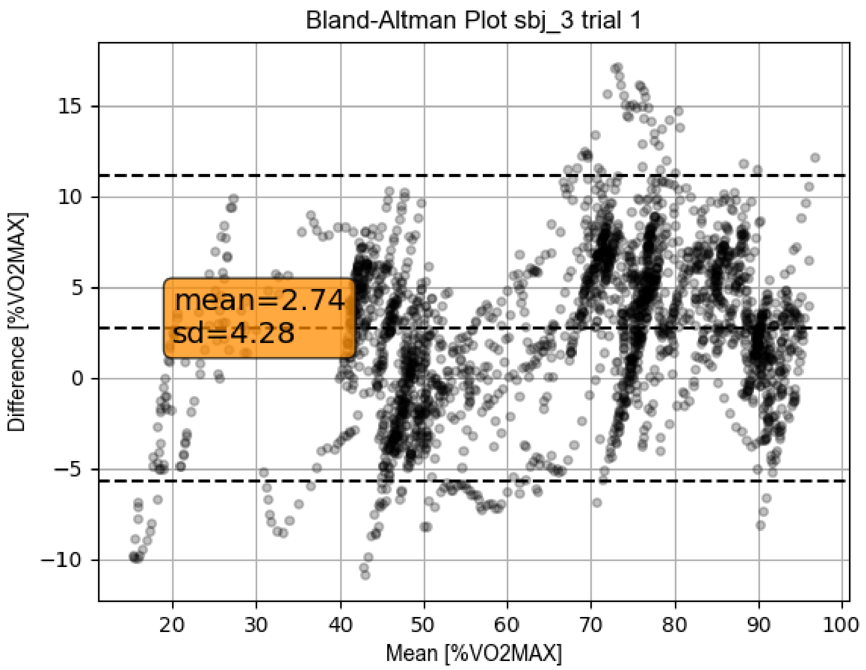

Figure 27: Bland-Altman plot is used to visually appreciate the agreement between actual and predicted $\dot{V}O_2$ responses.

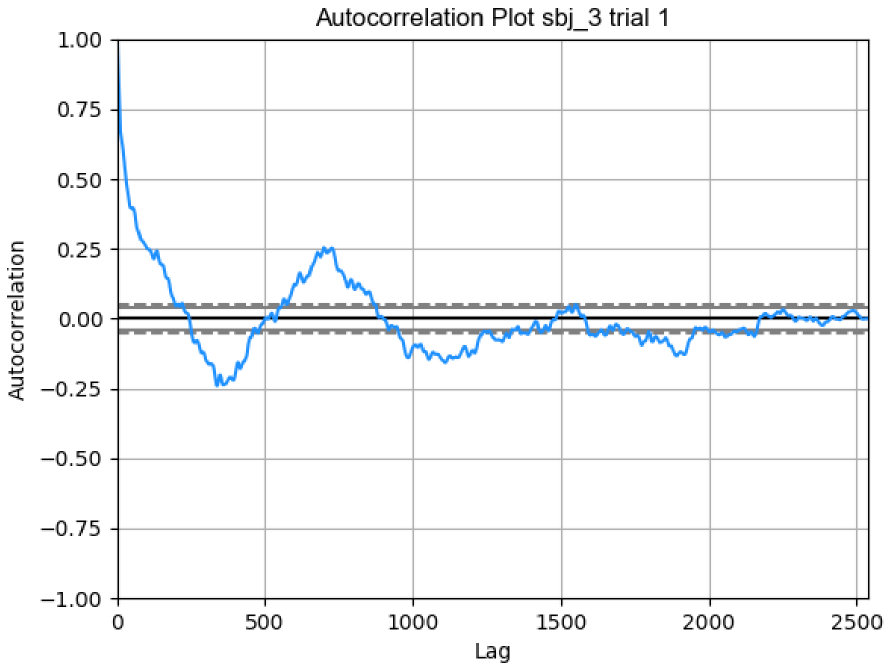

Figure 28: The autocorrelation plot (correlogram) is used to assess the correlation between the signal and the delayed (lagged) replication. Sample autocorrelation (blue line) is reported versus time lags. The horizontal (grey) lines displayed in the plot correspond to 95% and 99% (dashed) confidence bands.

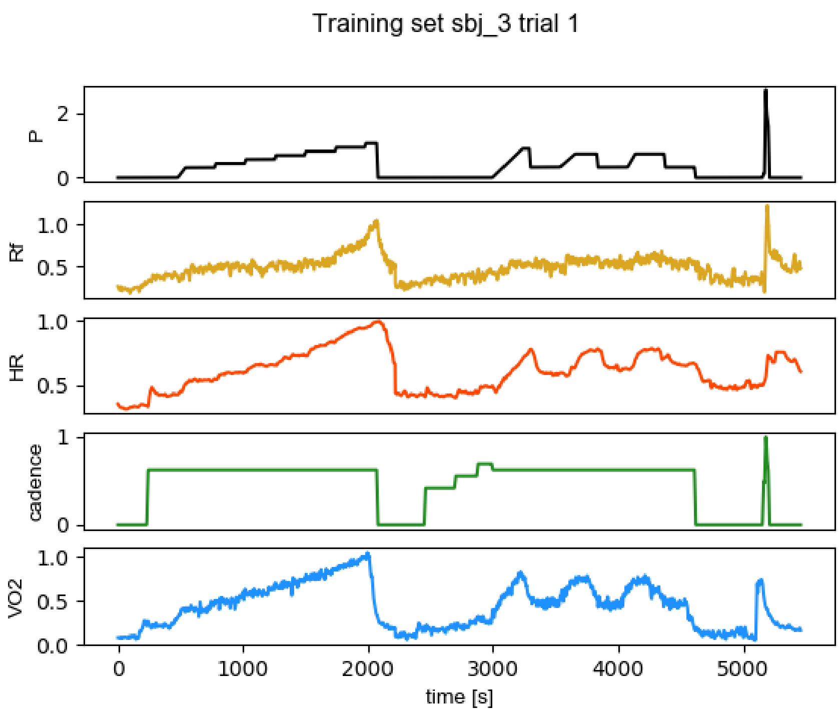

Figure 29: Training set include: power output (P, black), respiratory frequency (Rf, gold), heart rate (HR, red), cadence (ω, green) and $\dot{V}O_2$ (VO2, blue). Every quantity is normalised on the maximal value reported in the individual $.json$ file.

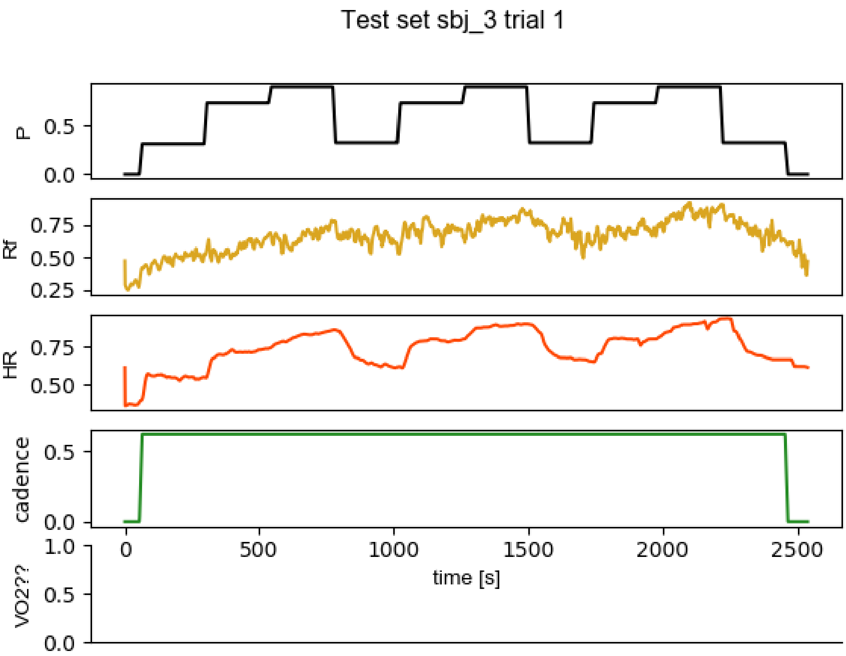

Figure 30: Test set include: power output (P, black), respiratory frequency (Rf, gold), heart rate (HR, red) and cadence (ω, green). The $\dot{V}O_2$ value is assumed to be unknown during the test set, therefore is left undefined in this plot. Every quantity is normalised on the maximal value reported in the individual $.json$ file.

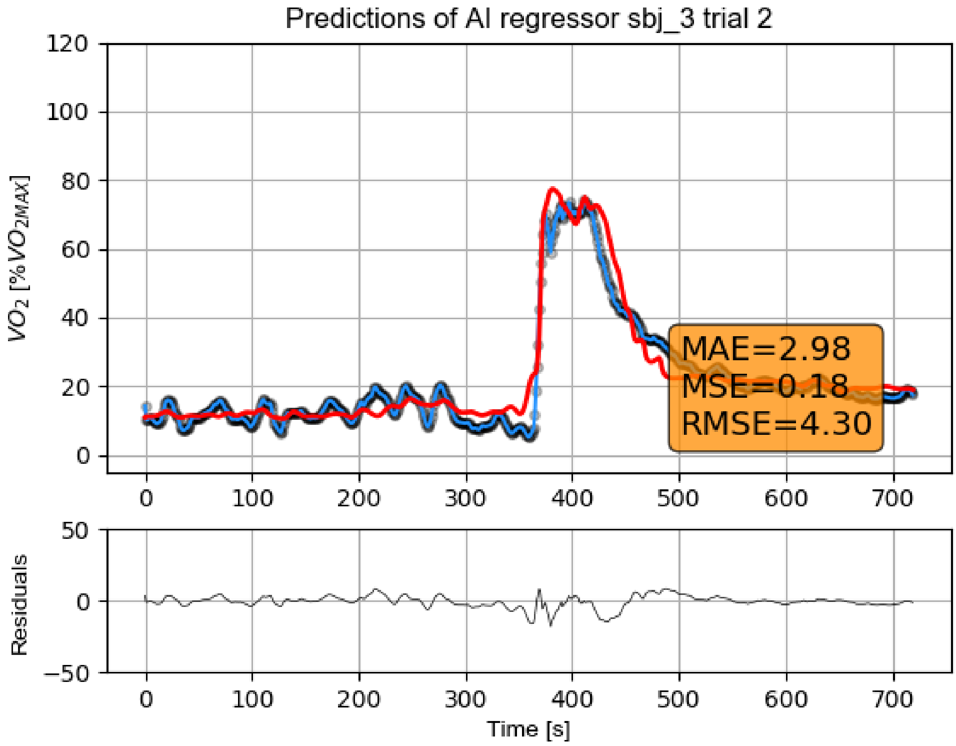

Figure 31: Oxygen uptake ($\dot{V}O_2$) experimental values (black semi-transparent dots and blue line) are compared to the estimated (red line) $\dot{V}O_2$ values. Residuals are computed as the difference between actual and estimated values. $\dot{V}O_2$ values are expressed as % of the individual's maximal $\dot{V}O_2$ (i.e. $\dot{V}O_{2MAX}$).

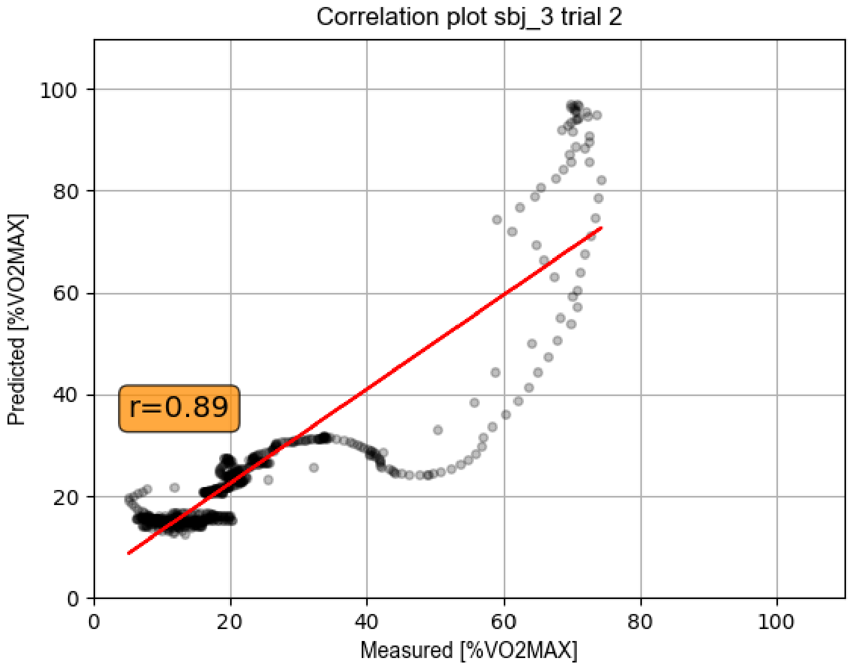

Figure 32: A scatterplot (semi-transparent black dots) that shows the relationship between actual and predicted $\dot{V}O_2$ responses. The red line represents the best-fit linear equation.

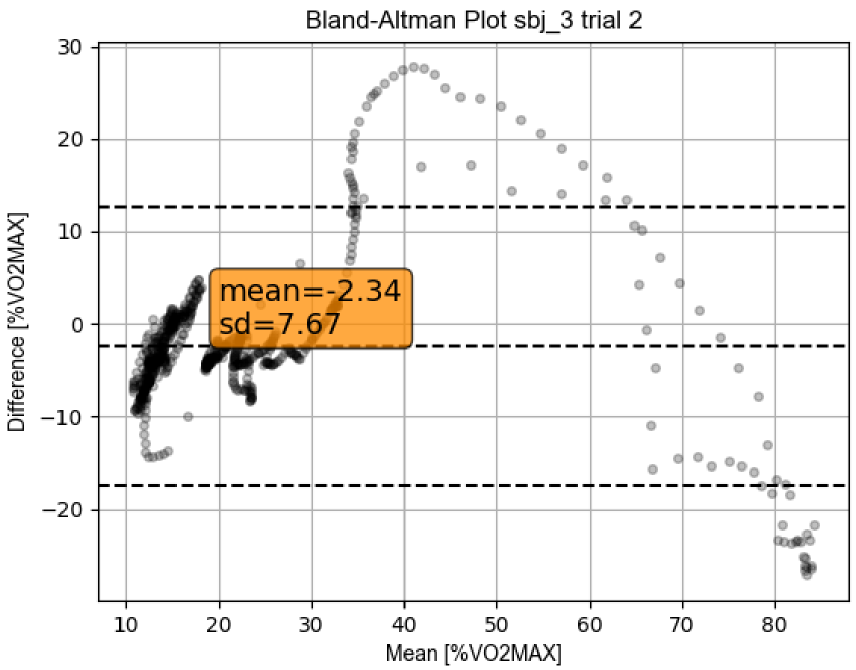

Figure 33: Bland-Altman plot is used to visually appreciate the agreement between actual and predicted $\dot{V}O_2$ responses.

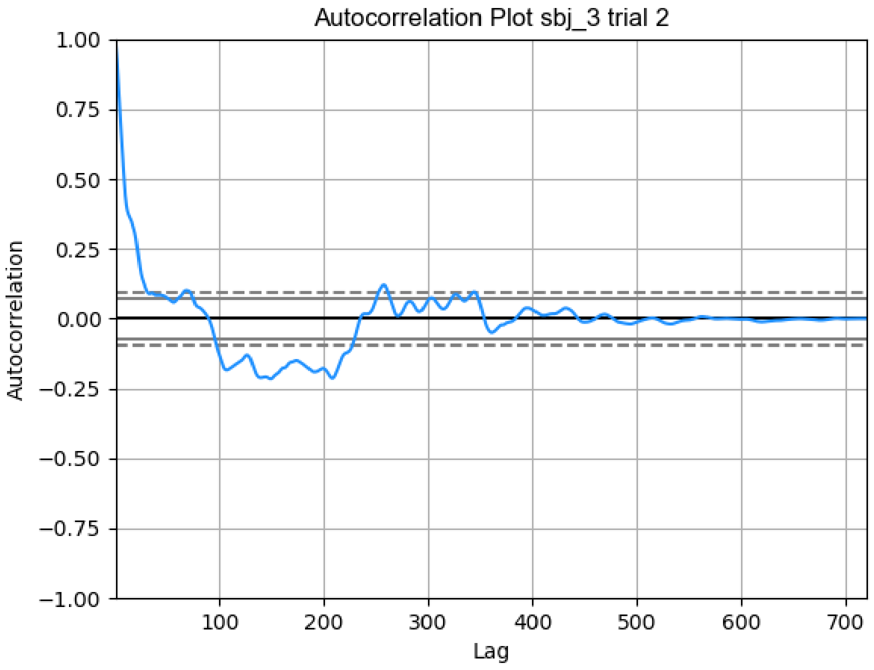

Figure 34: The autocorrelation plot (correlogram) is used to assess the correlation between the signal and the delayed (lagged) replication. Sample autocorrelation (blue line) is reported versus time lags. The horizontal (grey) lines displayed in the plot correspond to 95% and 99% (dashed) confidence bands.

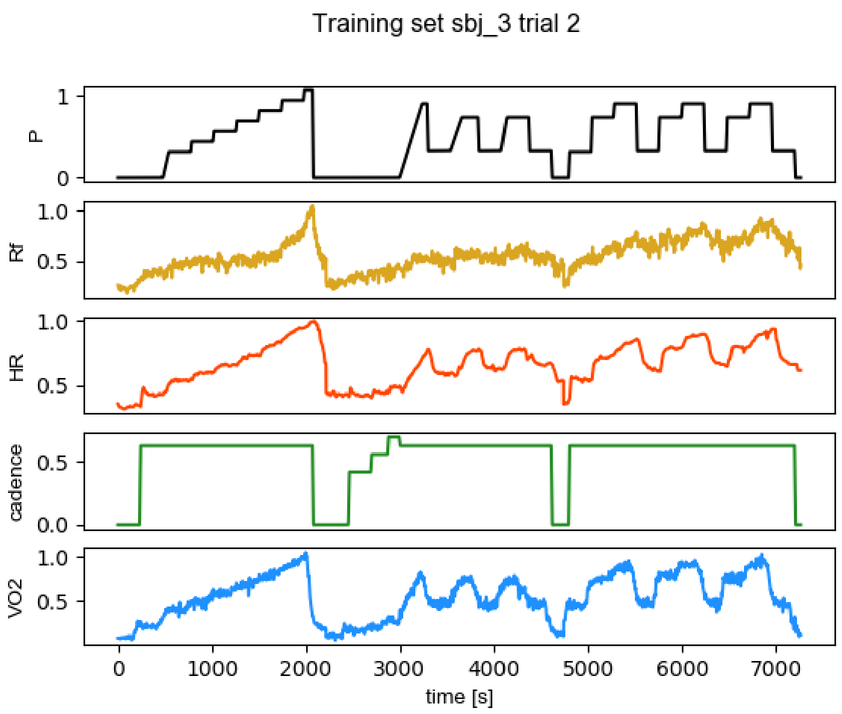

Figure 35: Training set include: power output (P, black), respiratory frequency (Rf, gold), heart rate (HR, red), cadence (ω, green) and $\dot{V}O_2$ (VO2, blue). Every quantity is normalised on the maximal value reported in the individual $.json$ file.

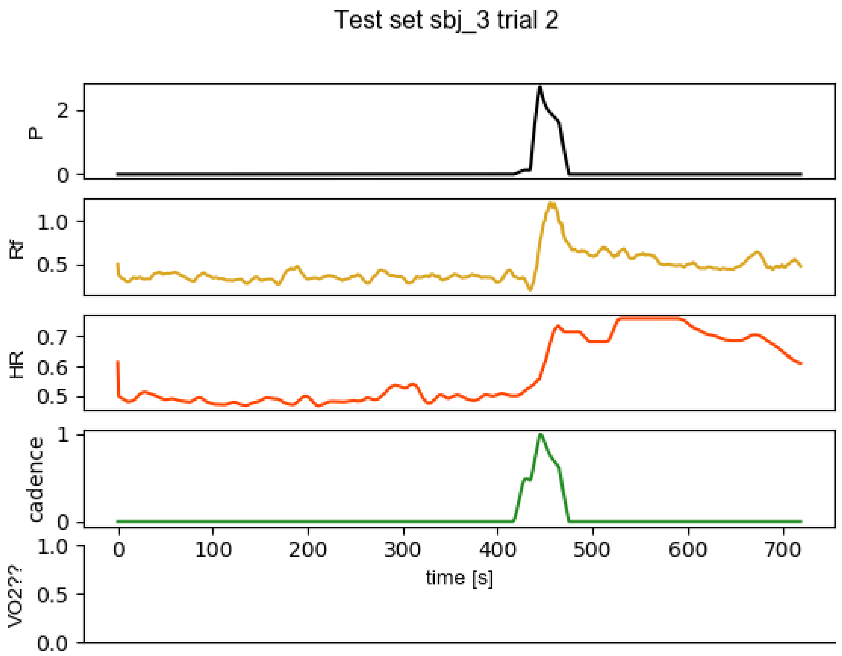

Figure 36: Test set include: power output (P, black), respiratory frequency (Rf, gold), heart rate (HR, red) and cadence (ω, green). The $\dot{V}O_2$ value is assumed to be unknown during the test set, therefore is left undefined in this plot. Every quantity is normalised on the maximal value reported in the individual $.json$ file.

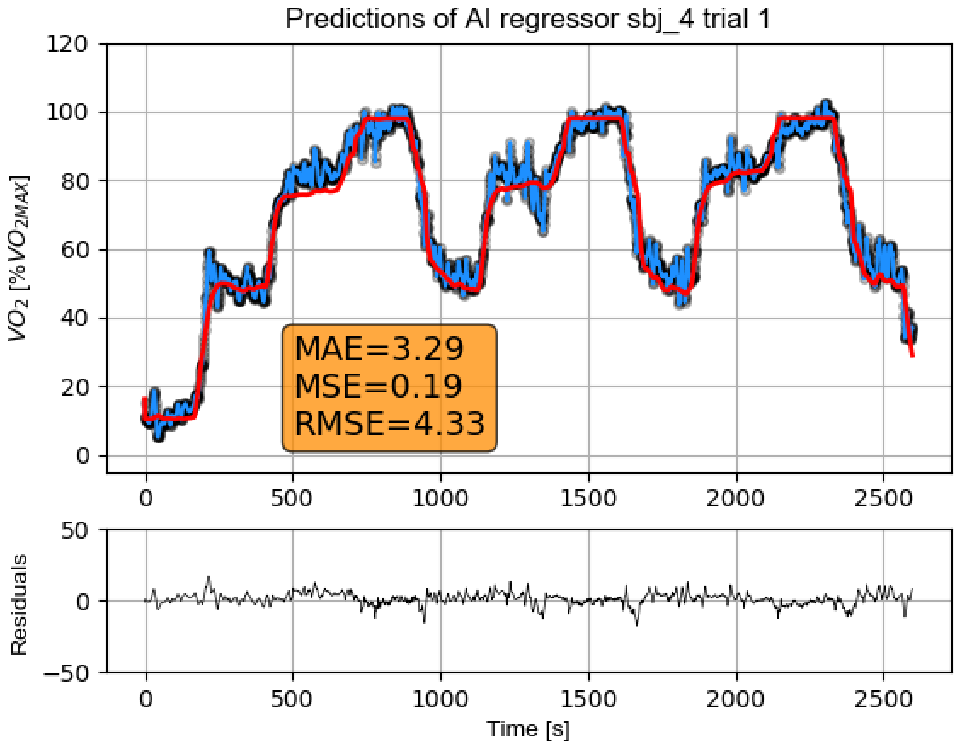

Figure 37: Oxygen uptake ($\dot{V}O_2$) experimental values (black semi-transparent dots and blue line) are compared to the estimated (red line) $\dot{V}O_2$ values. Residuals are computed as the difference between actual and estimated values. $\dot{V}O_2$ values are expressed as % of the individual's maximal $\dot{V}O_2$ (i.e. $\dot{V}O_{2MAX}$).

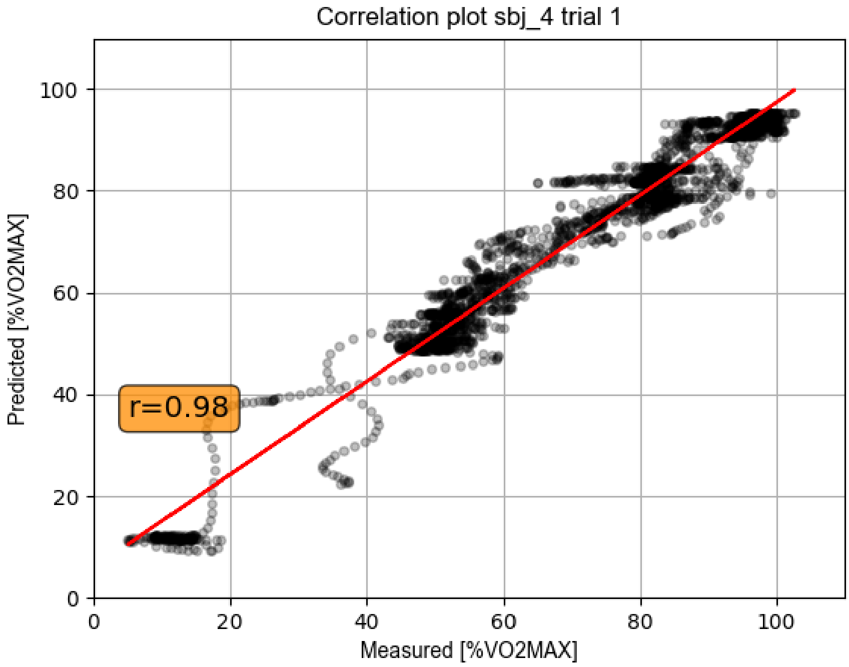

Figure 38: A scatterplot (semi-transparent black dots) that shows the relationship between actual and predicted $\dot{V}O_2$ responses. The red line represents the best-fit linear equation.

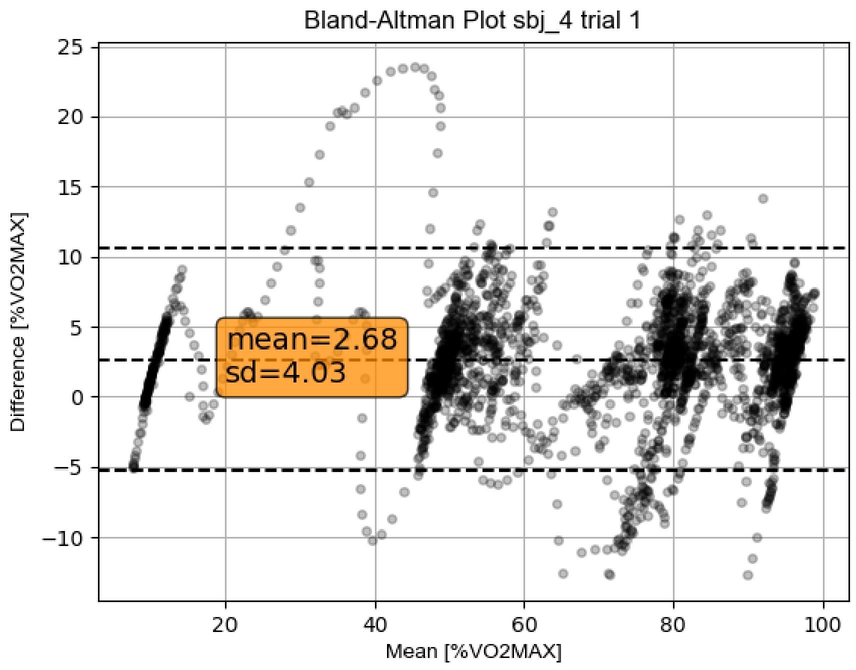

Figure 39: Bland-Altman plot is used to visually appreciate the agreement between actual and predicted $\dot{V}O_2$ responses.

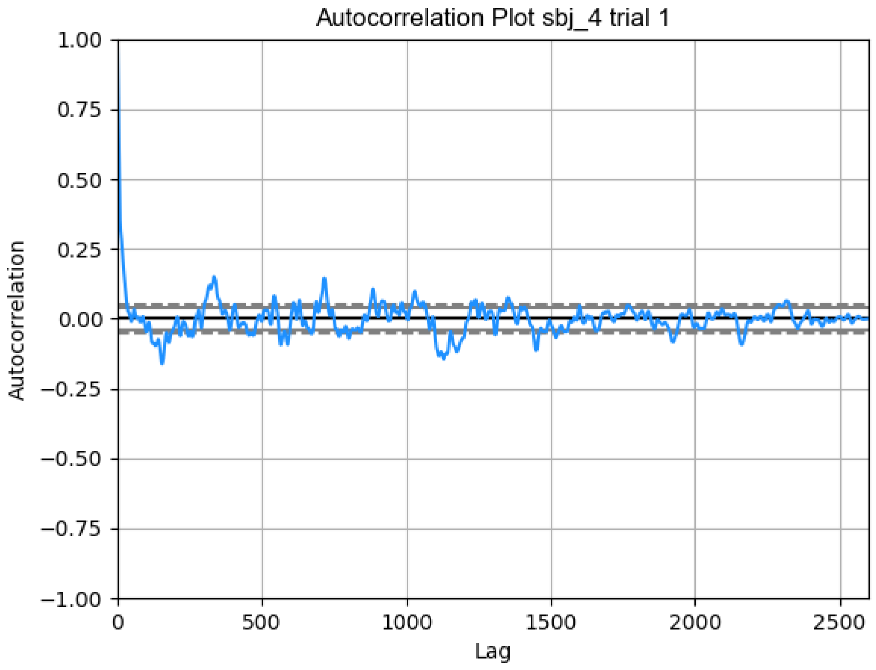

Figure 40: The autocorrelation plot (correlogram) is used to assess the correlation between the signal and the delayed (lagged) replication. Sample autocorrelation (blue line) is reported versus time lags. The horizontal (grey) lines displayed in the plot correspond to 95% and 99% (dashed) confidence bands.

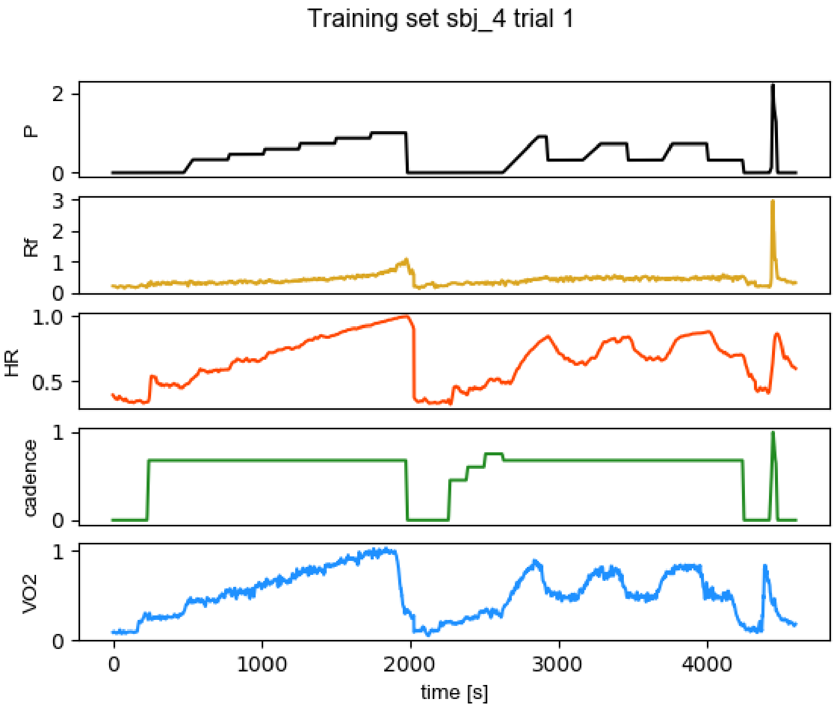

Figure 41: Training set include: power output (P, black), respiratory frequency (Rf, gold), heart rate (HR, red), cadence (ω, green) and $\dot{V}O_2$ (VO2, blue). Every quantity is normalised on the maximal value reported in the individual $.json$ file.

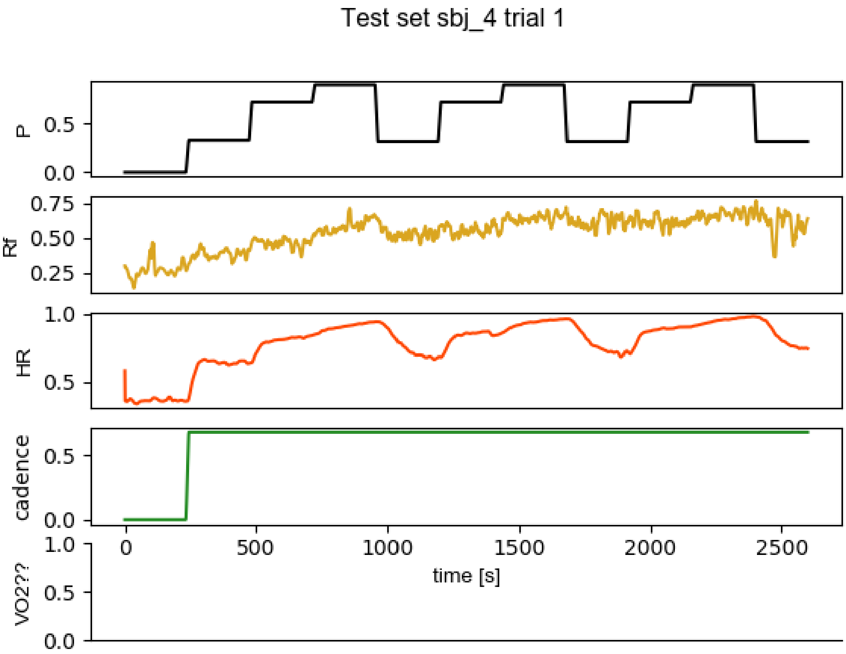

Figure 42: Test set include: power output (P, black), respiratory frequency (Rf, gold), heart rate (HR, red) and cadence (ω, green). The $\dot{V}O_2$ value is assumed to be unknown during the test set, therefore is left undefined in this plot. Every quantity is normalised on the maximal value reported in the individual $.json$ file.

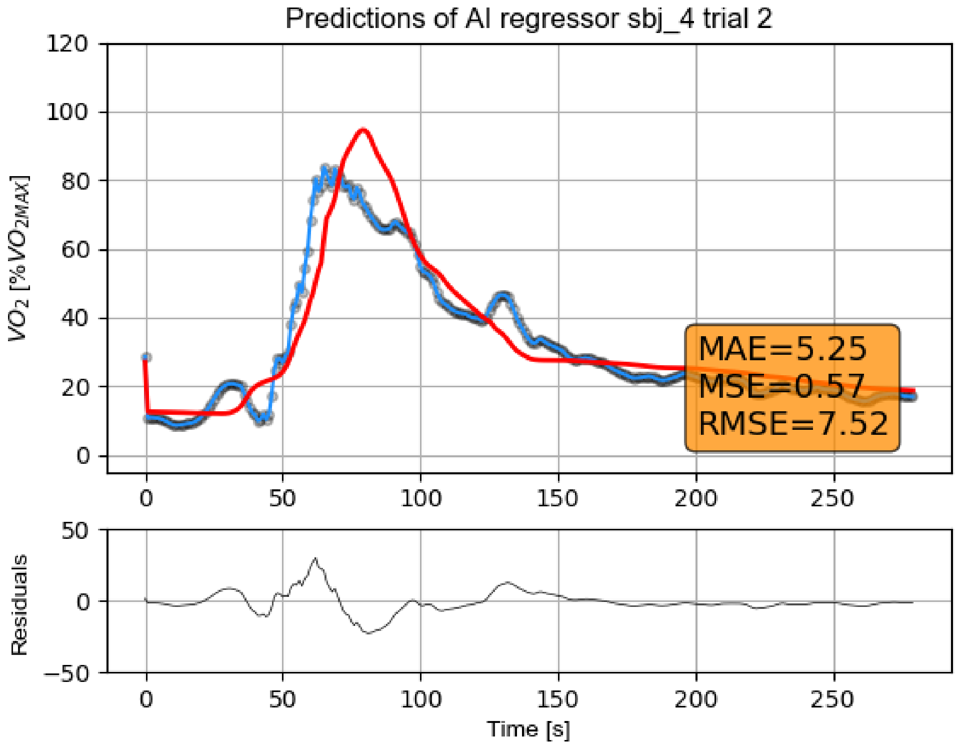

Figure 43: Oxygen uptake ($\dot{V}O_2$) experimental values (black semi-transparent dots and blue line) are compared to the estimated (red line) $\dot{V}O_2$ values. Residuals are computed as the difference between actual and estimated values. $\dot{V}O_2$ values are expressed as % of the individual's maximal $\dot{V}O_2$ (i.e. $\dot{V}O_{2MAX}$).

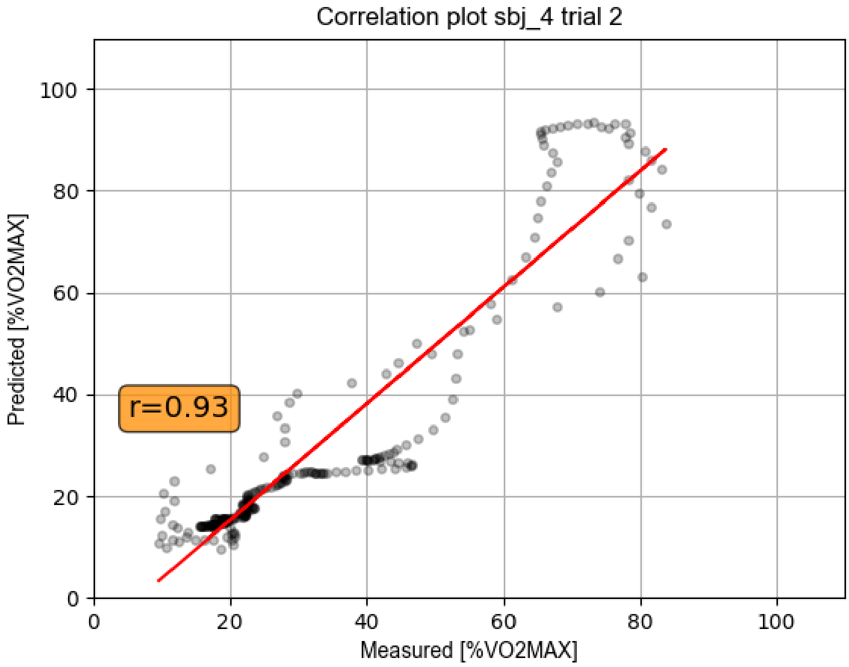

Figure 44: A scatterplot (semi-transparent black dots) that shows the relationship between actual and predicted $\dot{V}O_2$ responses. The red line represents the best-fit linear equation.

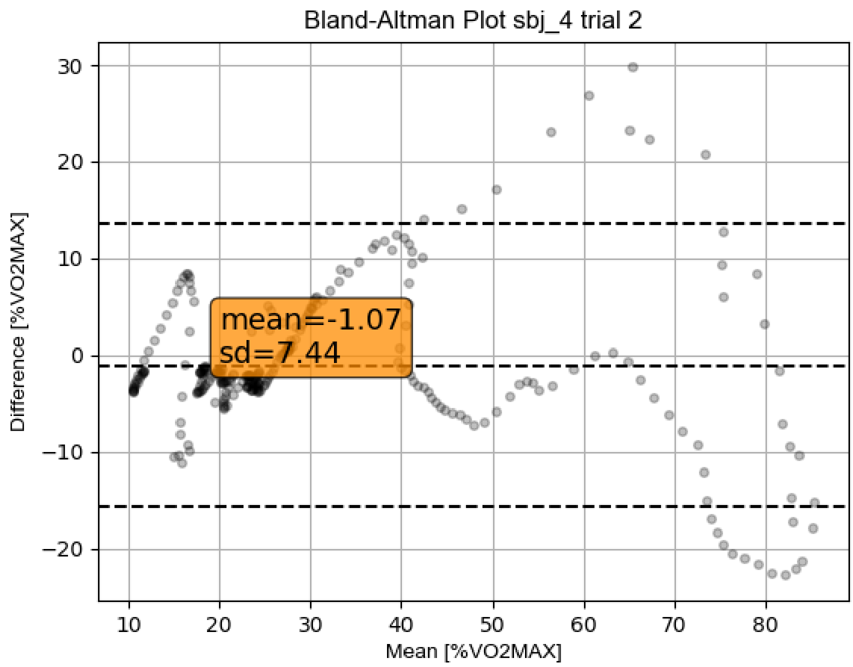

Figure 45: Bland-Altman plot is used to visually appreciate the agreement between actual and predicted $\dot{V}O_2$ responses.

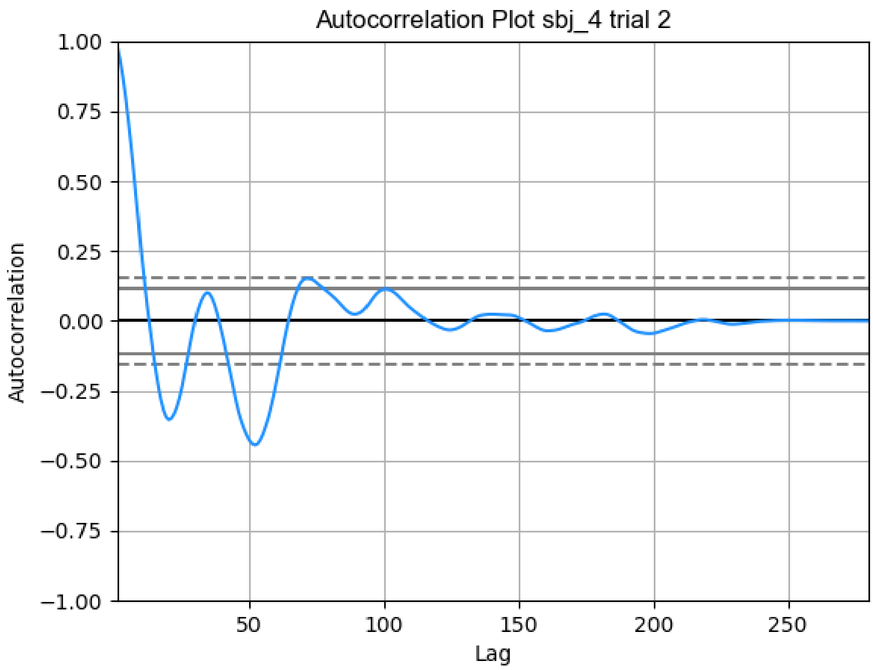

Figure 46: The autocorrelation plot (correlogram) is used to assess the correlation between the signal and the delayed (lagged) replication. Sample autocorrelation (blue line) is reported versus time lags. The horizontal (grey) lines displayed in the plot correspond to 95% and 99% (dashed) confidence bands.

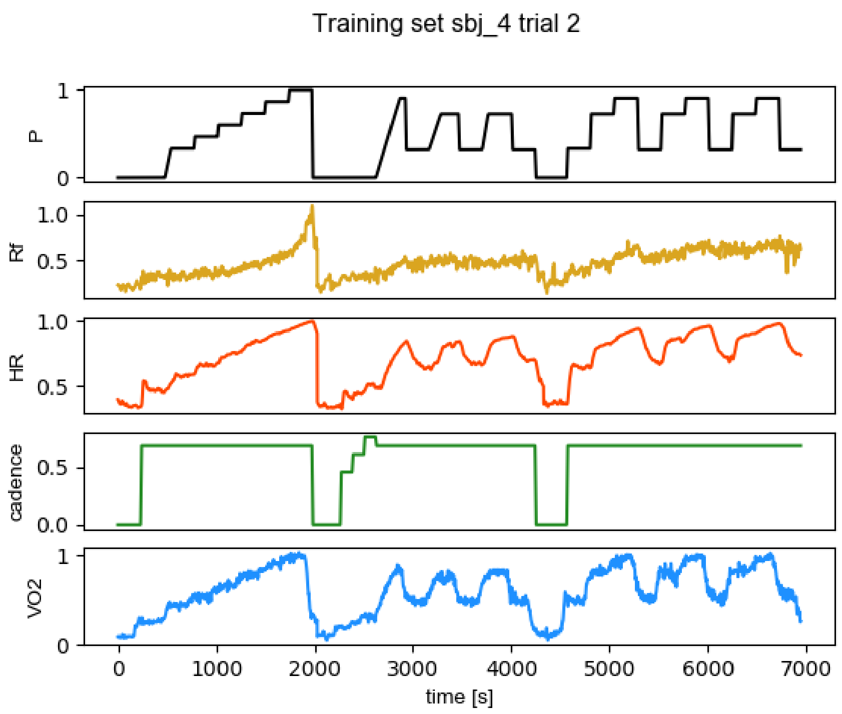

Figure 47: Training set include: power output (P, black), respiratory frequency (Rf, gold), heart rate (HR, red), cadence (ω, green) and $\dot{V}O_2$ (VO2, blue). Every quantity is normalised on the maximal value reported in the individual $.json$ file.

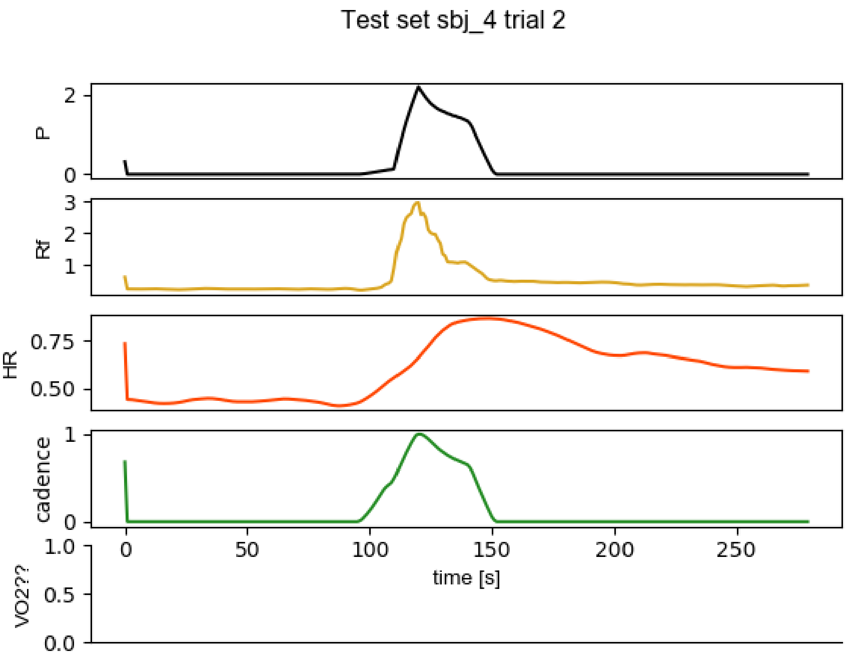

Figure 48: Test set include: power output (P, black), respiratory frequency (Rf, gold), heart rate (HR, red) and cadence (ω, green). The $\dot{V}O_2$ value is assumed to be unknown during the test set, therefore is left undefined in this plot. Every quantity is normalised on the maximal value reported in the individual $.json$ file.

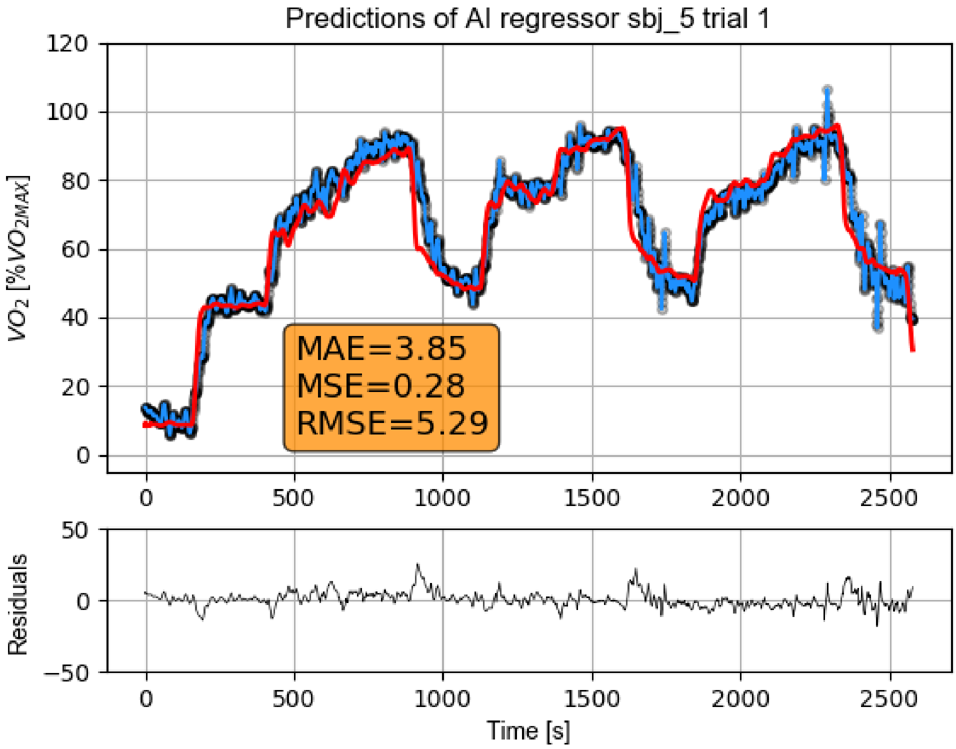

Figure 49: Oxygen uptake ($\dot{V}O_2$) experimental values (black semi-transparent dots and blue line) are compared to the estimated (red line) $\dot{V}O_2$ values. Residuals are computed as the difference between actual and estimated values. $\dot{V}O_2$ values are expressed as % of the individual's maximal $\dot{V}O_2$ (i.e. $\dot{V}O_{2MAX}$).

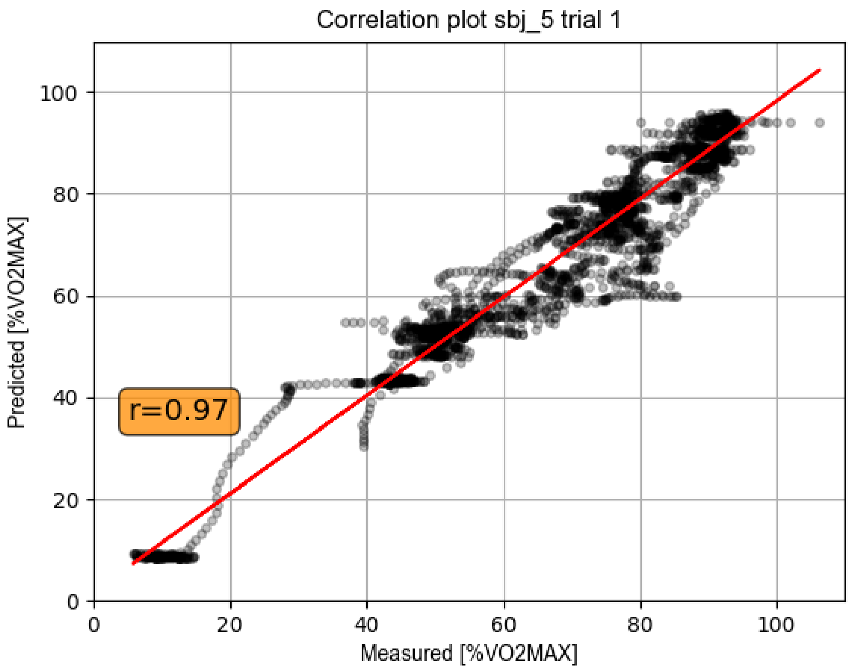

Figure 50: A scatterplot (semi-transparent black dots) that shows the relationship between actual and predicted $\dot{V}O_2$ responses. The red line represents the best-fit linear equation.

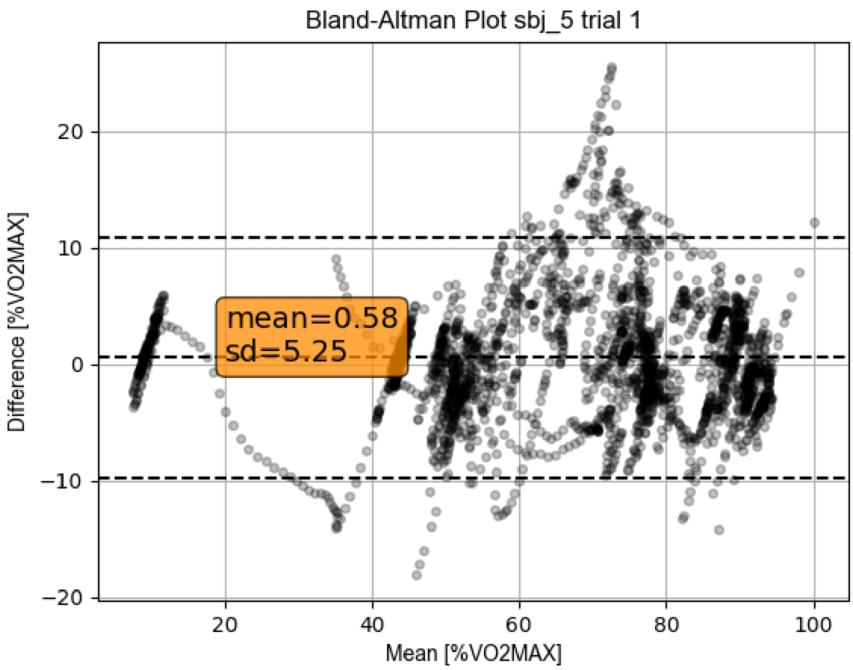

Figure 51: Bland-Altman plot is used to visually appreciate the agreement between actual and predicted $\dot{V}O_2$ responses.

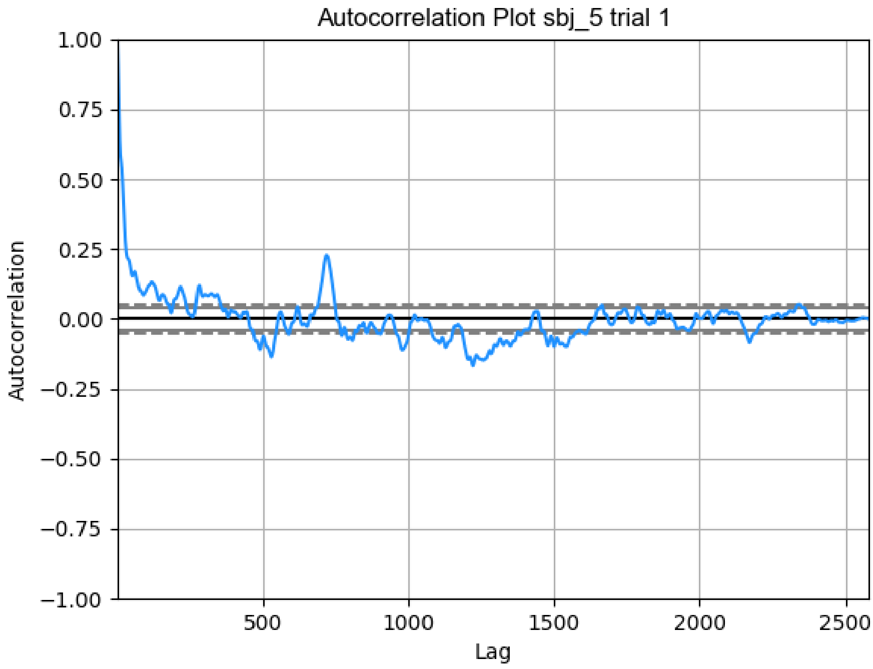

Figure 52: The autocorrelation plot (correlogram) is used to assess the correlation between the signal and the delayed (lagged) replication. Sample autocorrelation (blue line) is reported versus time lags. The horizontal (grey) lines displayed in the plot correspond to 95% and 99% (dashed) confidence bands.

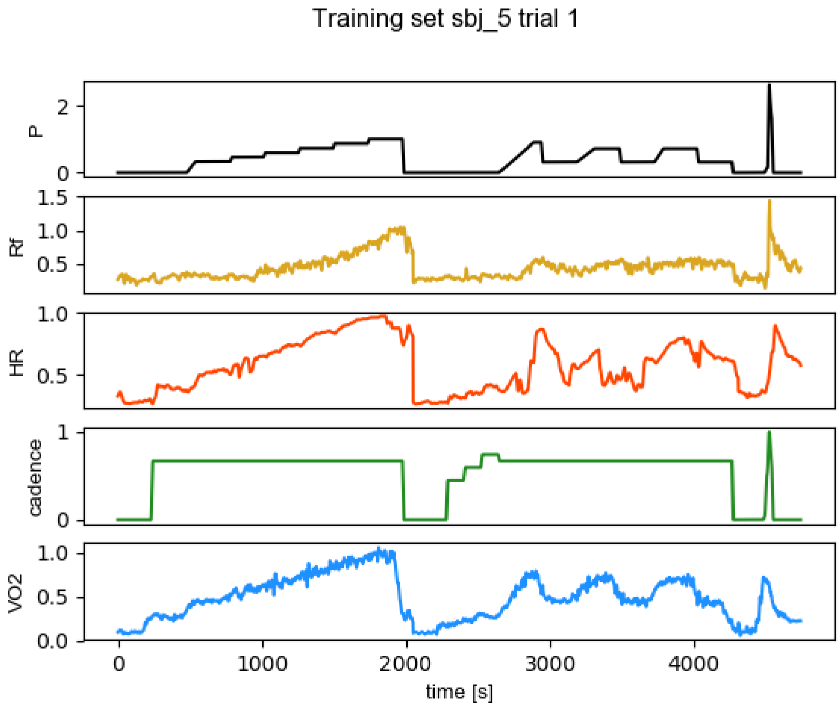

Figure 53: Training set include: power output (P, black), respiratory frequency (Rf, gold), heart rate (HR, red), cadence (ω, green) and $\dot{V}O_2$ (VO2, blue). Every quantity is normalised on the maximal value reported in the individual $.json$ file.

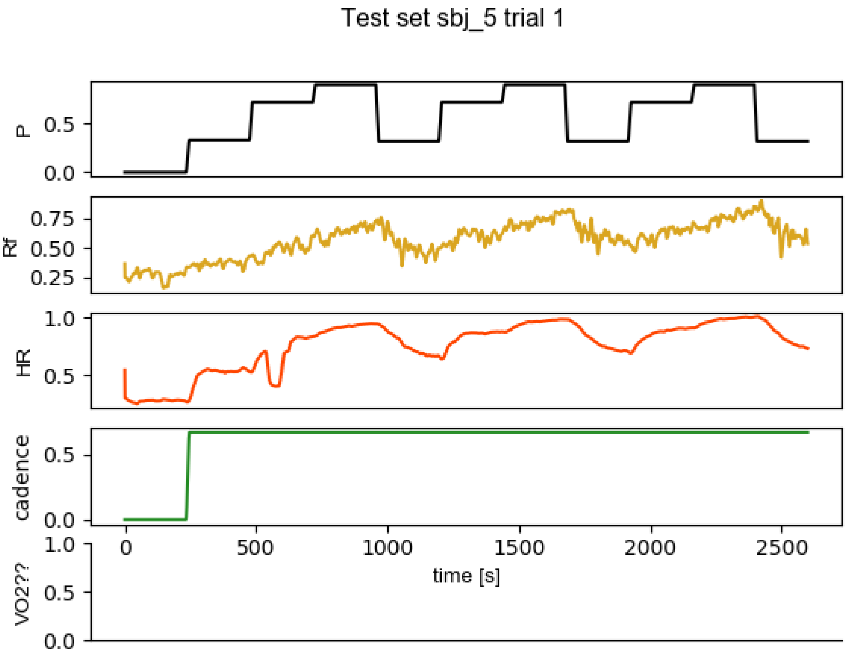

Figure 54: Test set include: power output (P, black), respiratory frequency (Rf, gold), heart rate (HR, red) and cadence (ω, green). The $\dot{V}O_2$ value is assumed to be unknown during the test set, therefore is left undefined in this plot. Every quantity is normalised on the maximal value reported in the individual $.json$ file.

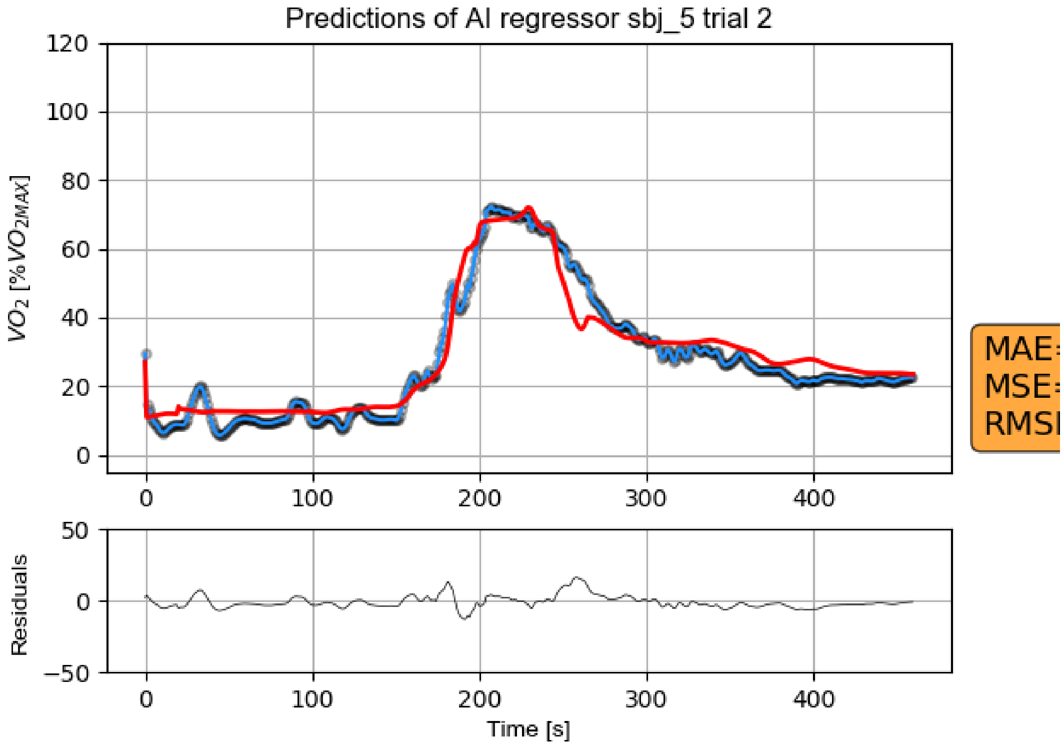

Figure 55: Oxygen uptake ($\dot{V}O_2$) experimental values (black semi-transparent dots and blue line) are compared to the estimated (red line) $\dot{V}O_2$ values. Residuals are computed as the difference between actual and estimated values. $\dot{V}O_2$ values are expressed as % of the individual's maximal $\dot{V}O_2$ (i.e. $\dot{V}O_{2MAX}$).

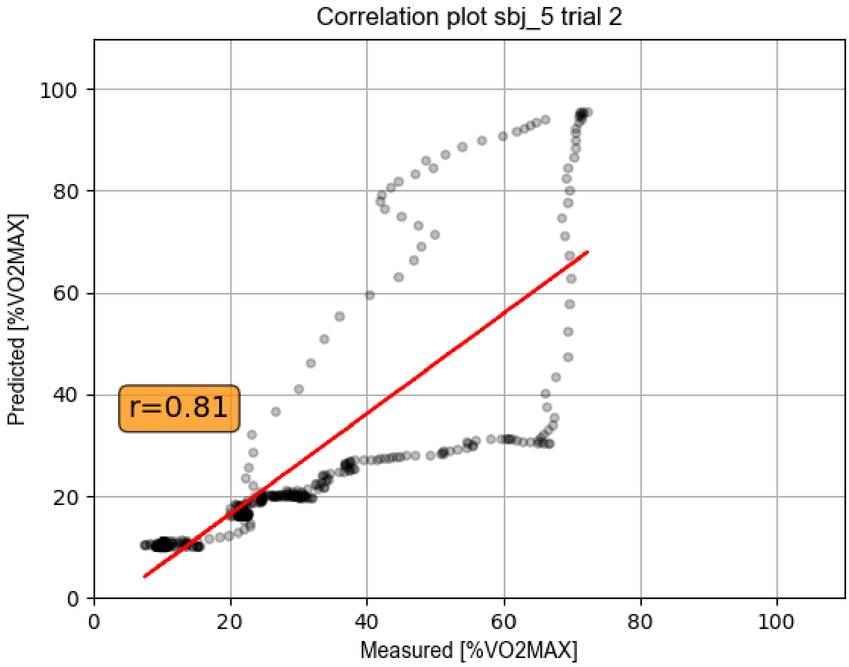

Figure 56: A scatterplot (semi-transparent black dots) that shows the relationship between actual and predicted $\dot{V}O_2$ responses. The red line represents the best-fit linear equation.

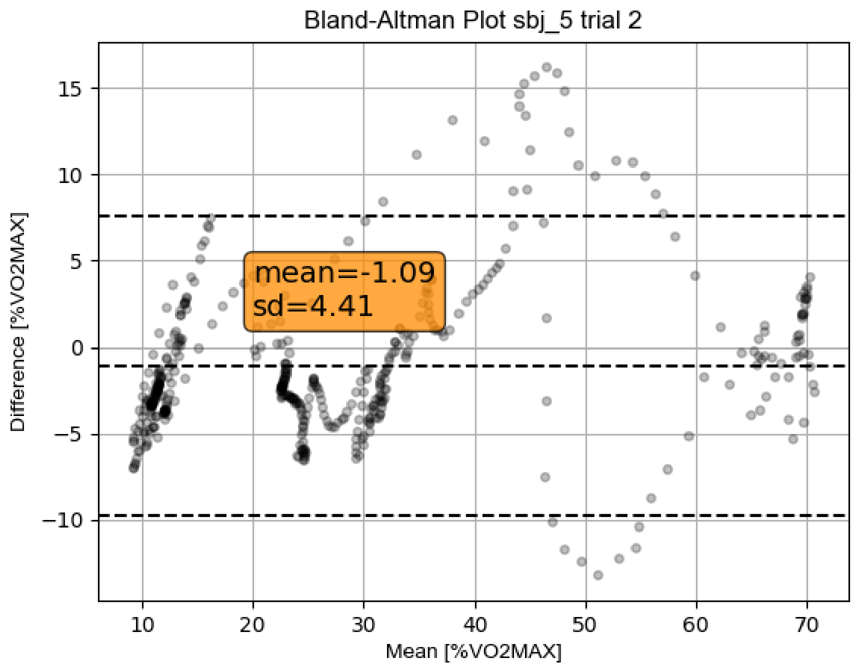

Figure 57: Bland-Altman plot is used to visually appreciate the agreement between actual and predicted $\dot{V}O_2$ responses.

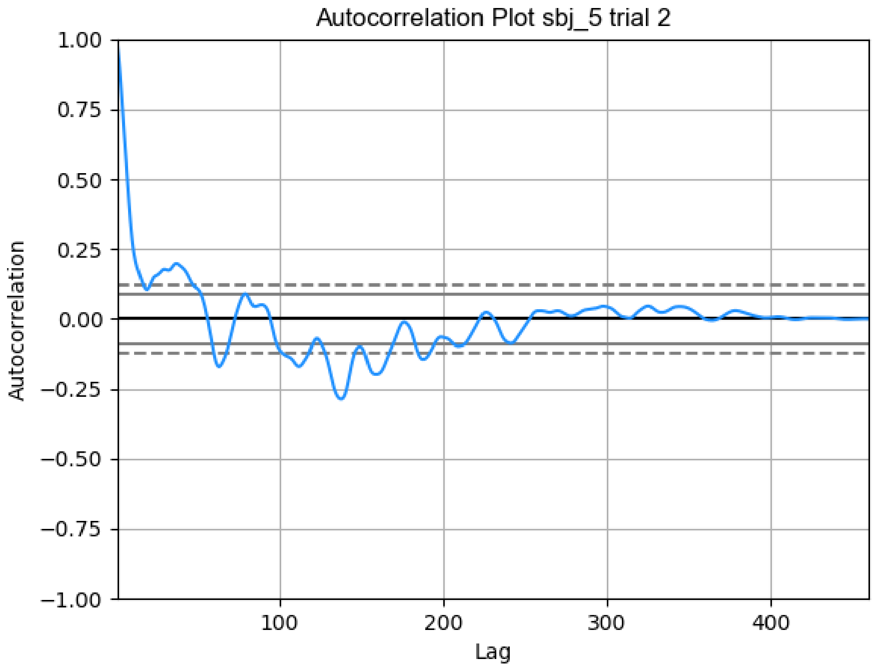

Figure 58: The autocorrelation plot (correlogram) is used to assess the correlation between the signal and the delayed (lagged) replication. Sample autocorrelation (blue line) is reported versus time lags. The horizontal (grey) lines displayed in the plot correspond to 95% and 99% (dashed) confidence bands.

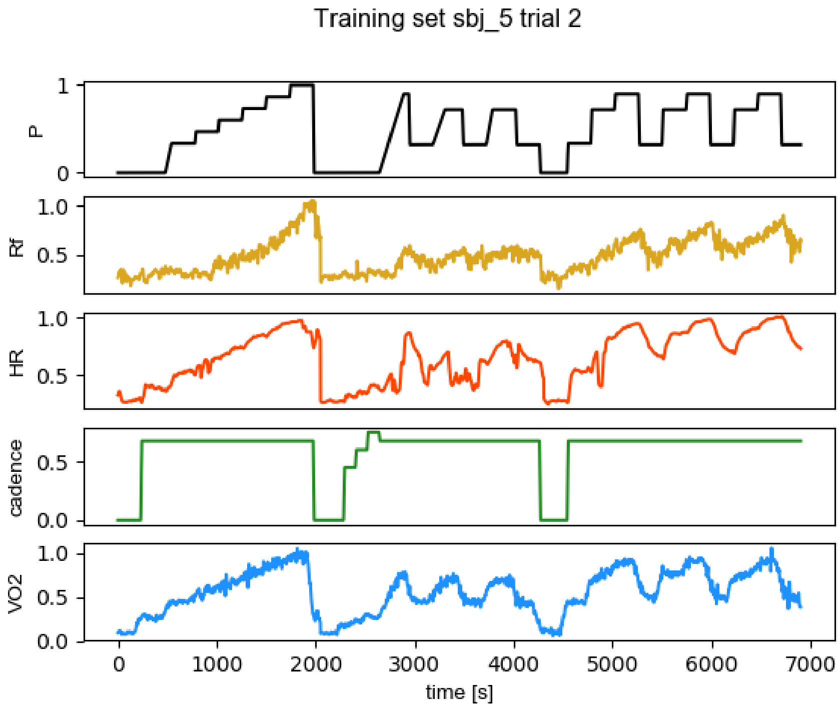

Figure 59: Training set include: power output (P, black), respiratory frequency (Rf, gold), heart rate (HR, red), cadence (ω, green) and $\dot{V}O_2$ (VO2, blue). Every quantity is normalised on the maximal value reported in the individual $.json$ file.

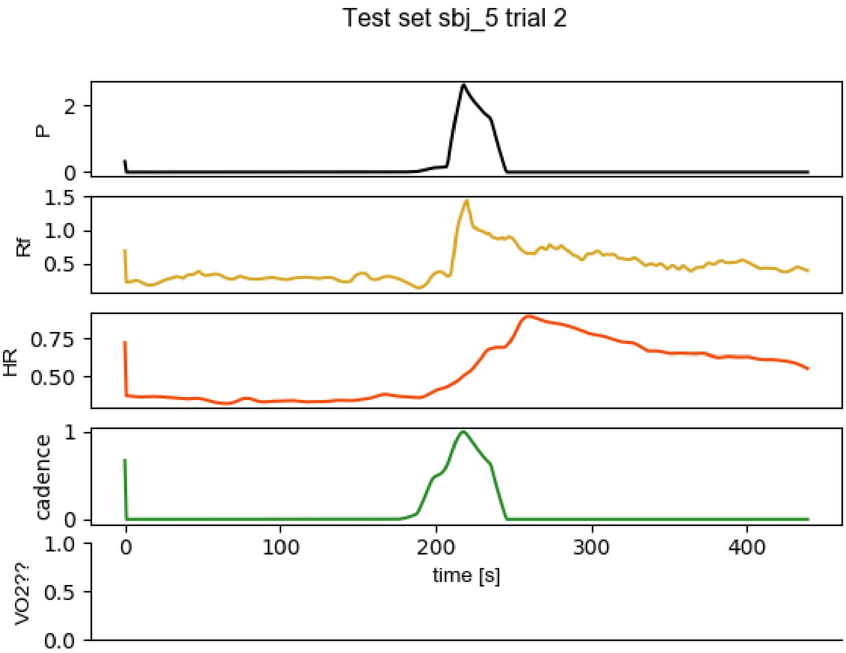

Figure 60: Test set include: power output (P, black), respiratory frequency (Rf, gold), heart rate (HR, red) and cadence (ω, green). The $\dot{V}O_2$ value is assumed to be unknown during the test set, therefore is left undefined in this plot. Every quantity is normalised on the maximal value reported in the individual $.json$ file.

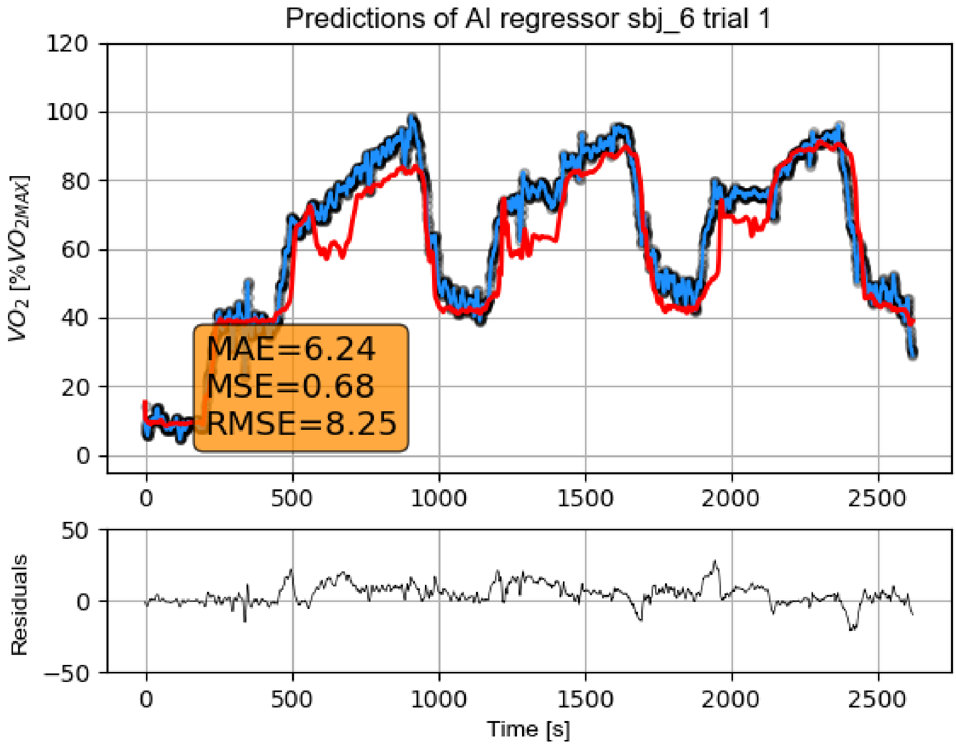

Figure 61: Oxygen uptake ($\dot{V}O_2$) experimental values (black semi-transparent dots and blue line) are compared to the estimated (red line) $\dot{V}O_2$ values. Residuals are computed as the difference between actual and estimated values. $\dot{V}O_2$ values are expressed as % of the individual's maximal $\dot{V}O_2$ (i.e. $\dot{V}O_{2MAX}$).

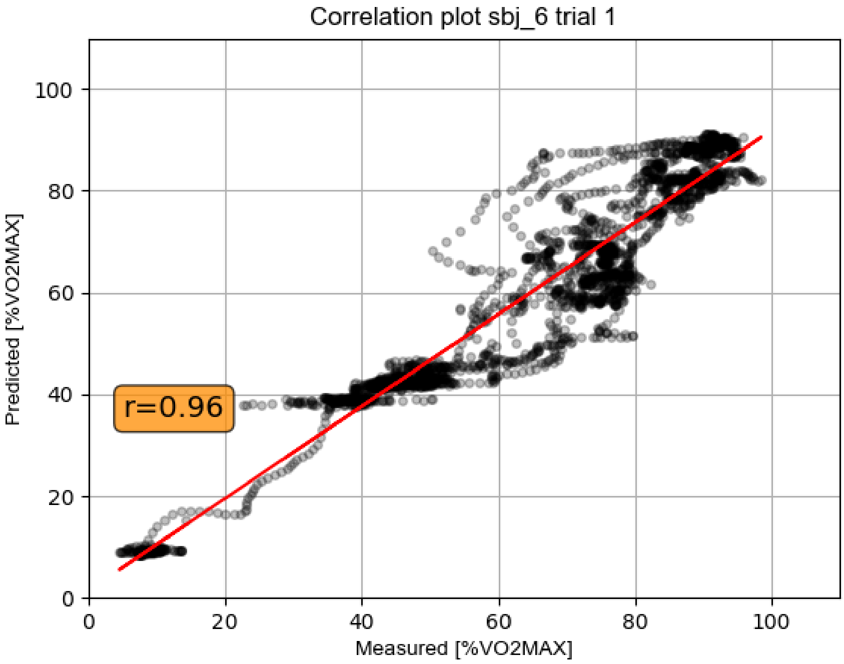

Figure 62: A scatterplot (semi-transparent black dots) that shows the relationship between actual and predicted $\dot{V}O_2$ responses. The red line represents the best-fit linear equation.

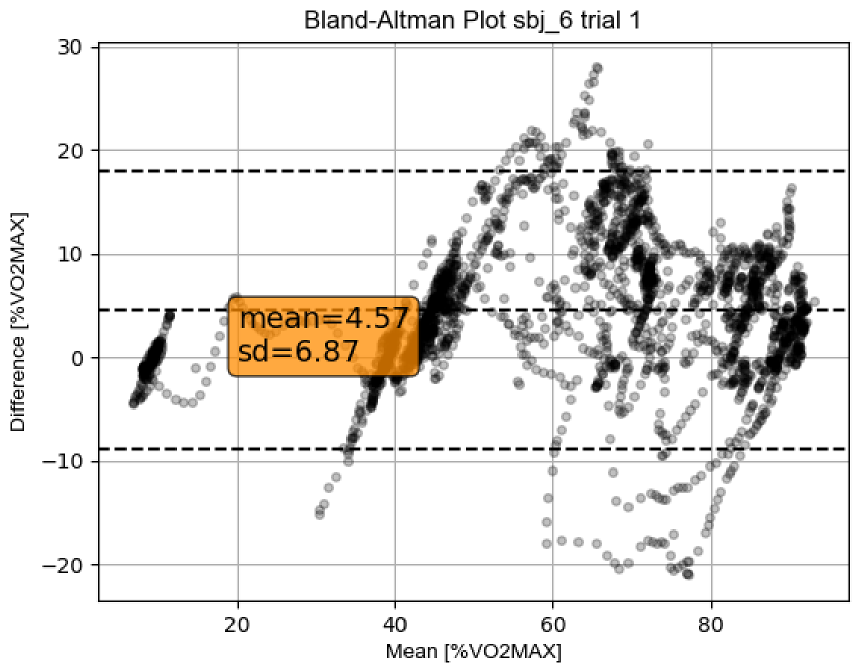

Figure 63: Bland-Altman plot is used to visually appreciate the agreement between actual and predicted $\dot{V}O_2$ responses.

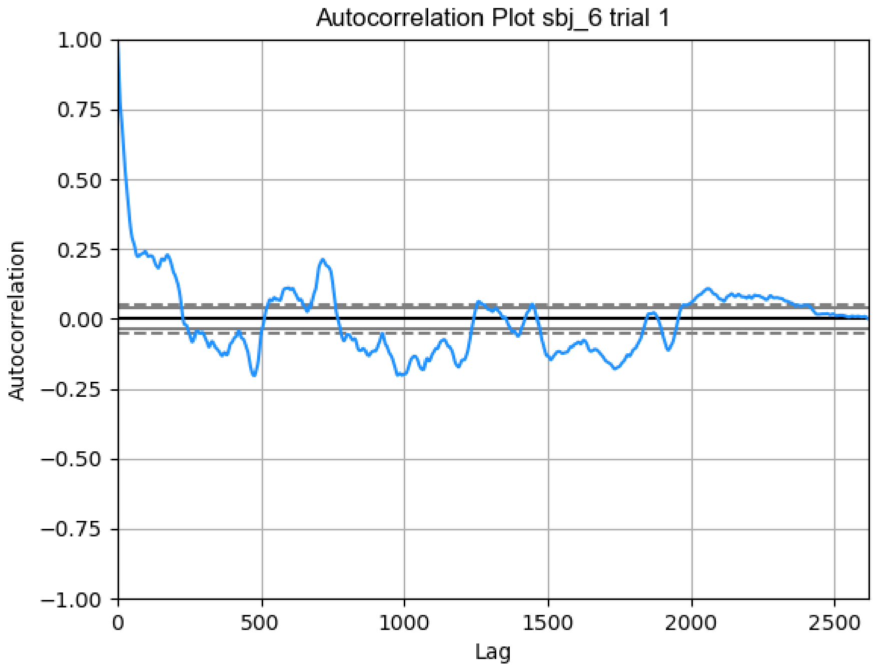

Figure 64: The autocorrelation plot (correlogram) is used to assess the correlation between the signal and the delayed (lagged) replication. Sample autocorrelation (blue line) is reported versus time lags. The horizontal (grey) lines displayed in the plot correspond to 95% and 99% (dashed) confidence bands.

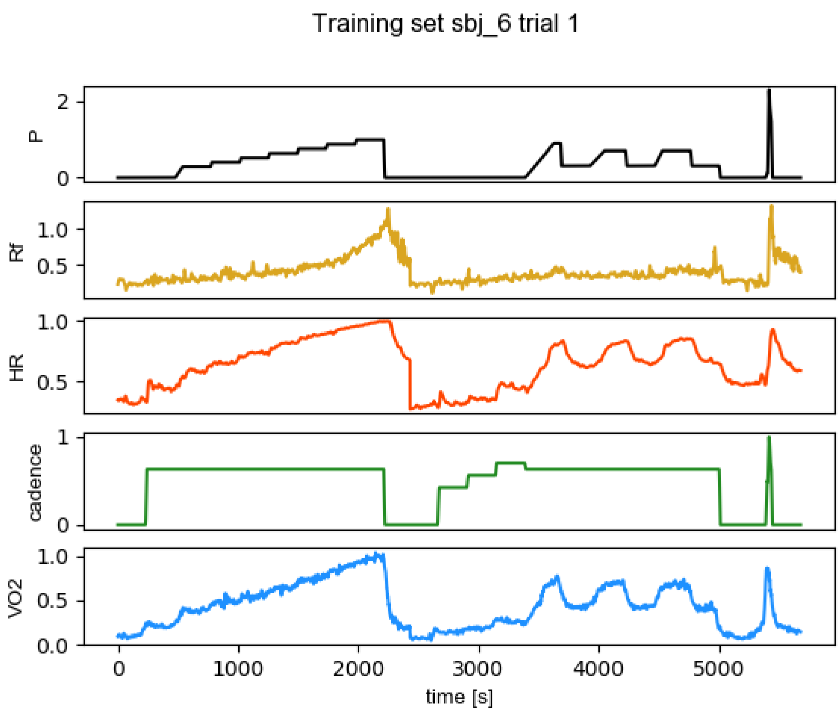

Figure 65: Training set include: power output (P, black), respiratory frequency (Rf, gold), heart rate (HR, red), cadence (ω, green) and $\dot{V}O_2$ (VO2, blue). Every quantity is normalised on the maximal value reported in the individual $.json$ file.

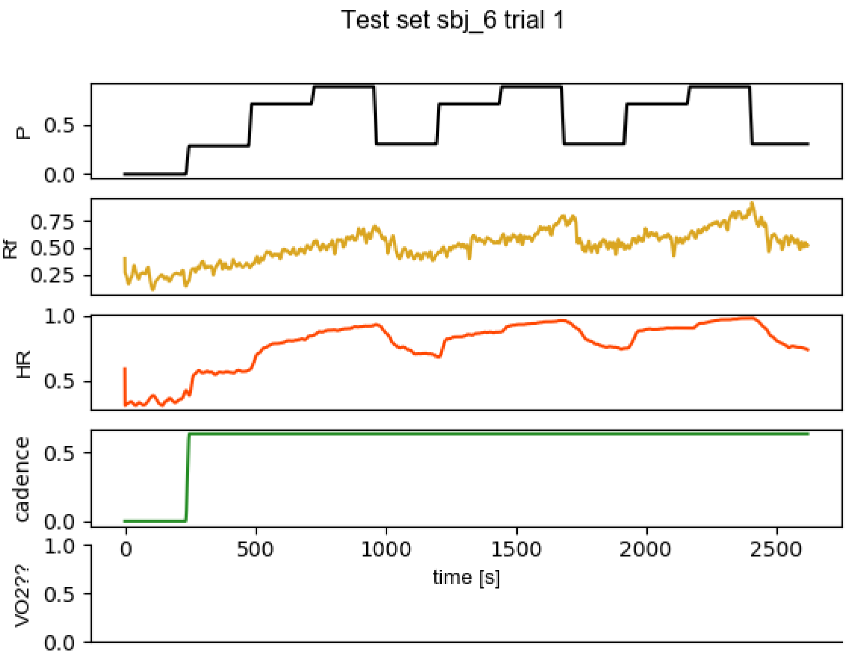

Figure 66: Test set include: power output (P, black), respiratory frequency (Rf, gold), heart rate (HR, red) and cadence (ω, green). The $\dot{V}O_2$ value is assumed to be unknown during the test set, therefore is left undefined in this plot. Every quantity is normalised on the maximal value reported in the individual $.json$ file.

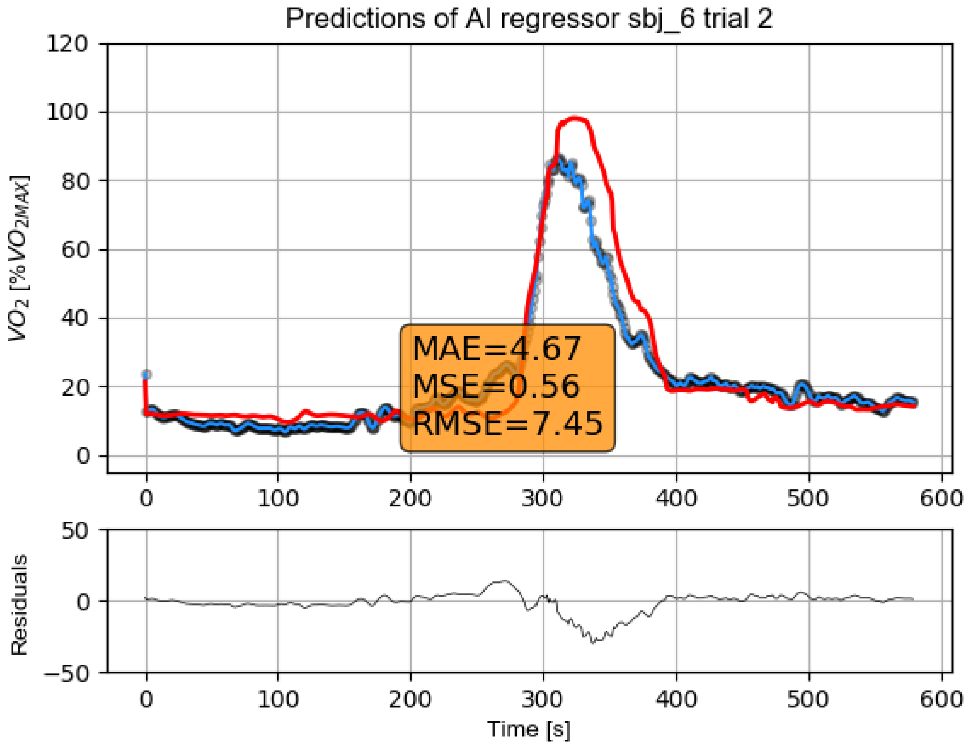

Figure 67: Oxygen uptake ($\dot{V}O_2$) experimental values (black semi-transparent dots and blue line) are compared to the estimated (red line) $\dot{V}O_2$ values. Residuals are computed as the difference between actual and estimated values. $\dot{V}O_2$ values are expressed as % of the individual's maximal $\dot{V}O_2$ (i.e. $\dot{V}O_{2MAX}$).

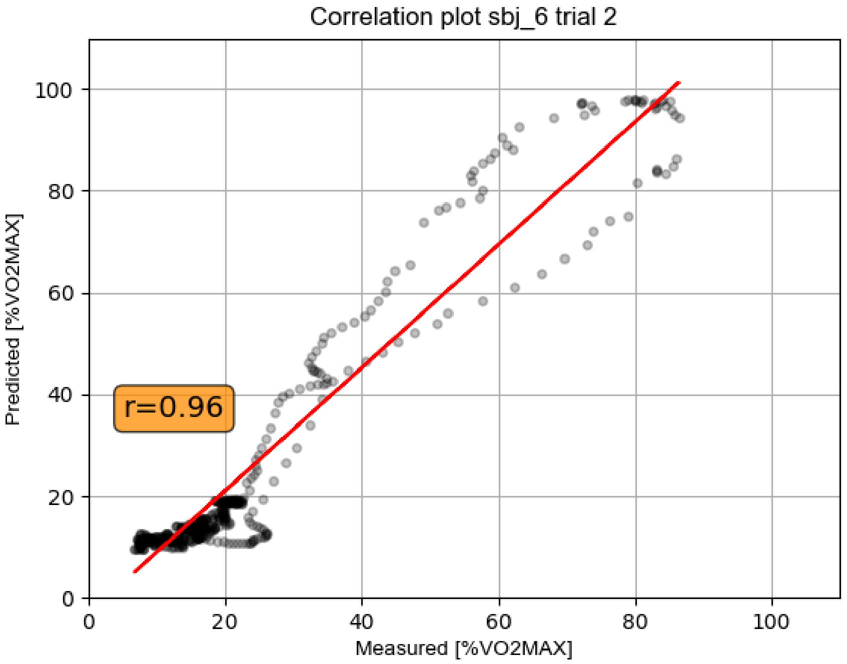

Figure 68: A scatterplot (semi-transparent black dots) that shows the relationship between actual and predicted $\dot{V}O_2$ responses. The red line represents the best-fit linear equation.

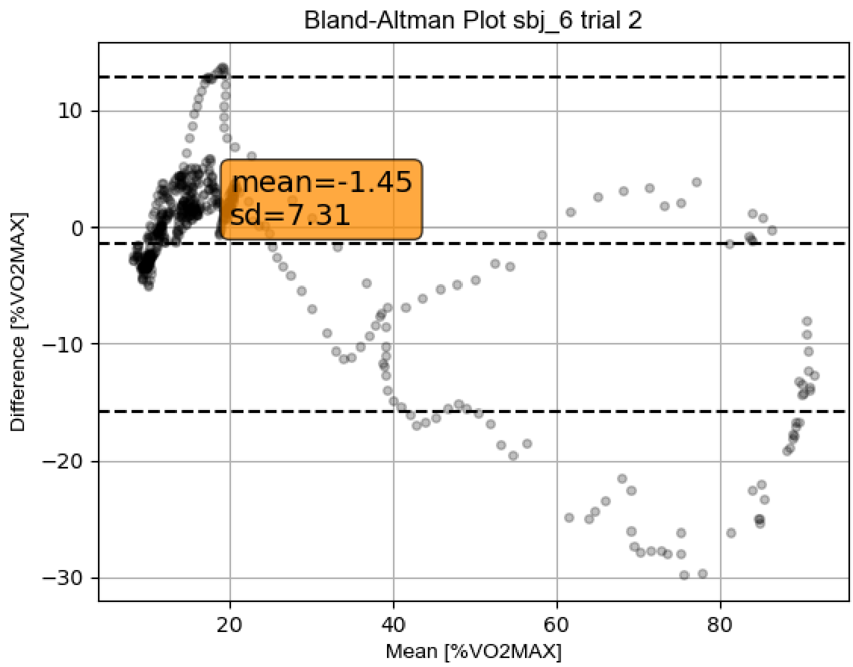

Figure 69: Bland-Altman plot is used to visually appreciate the agreement between actual and predicted $\dot{V}O_2$ responses.

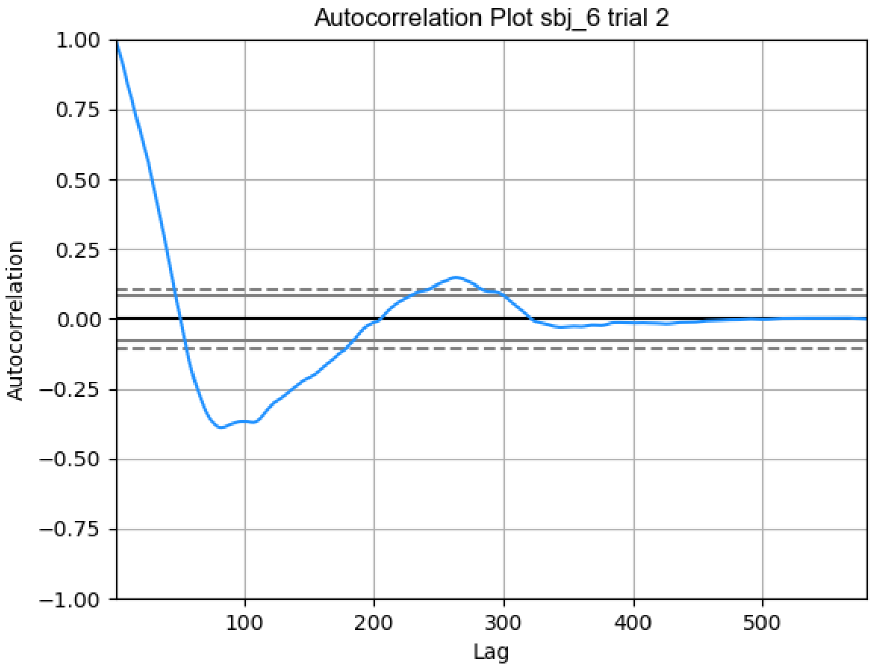

Figure 70: The autocorrelation plot (correlogram) is used to assess the correlation between the signal and the delayed (lagged) replication. Sample autocorrelation (blue line) is reported versus time lags. The horizontal (grey) lines displayed in the plot correspond to 95% and 99% (dashed) confidence bands.

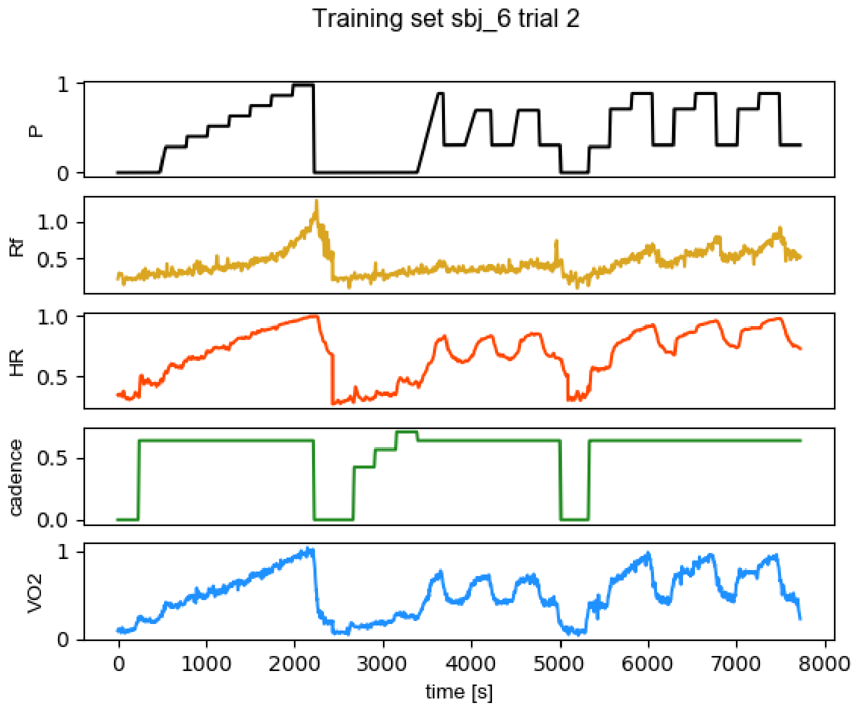

Figure 71: Training set include: power output (P, black), respiratory frequency (Rf, gold), heart rate (HR, red), cadence (ω, green) and $\dot{V}O_2$ (VO2, blue). Every quantity is normalised on the maximal value reported in the individual $.json$ file.

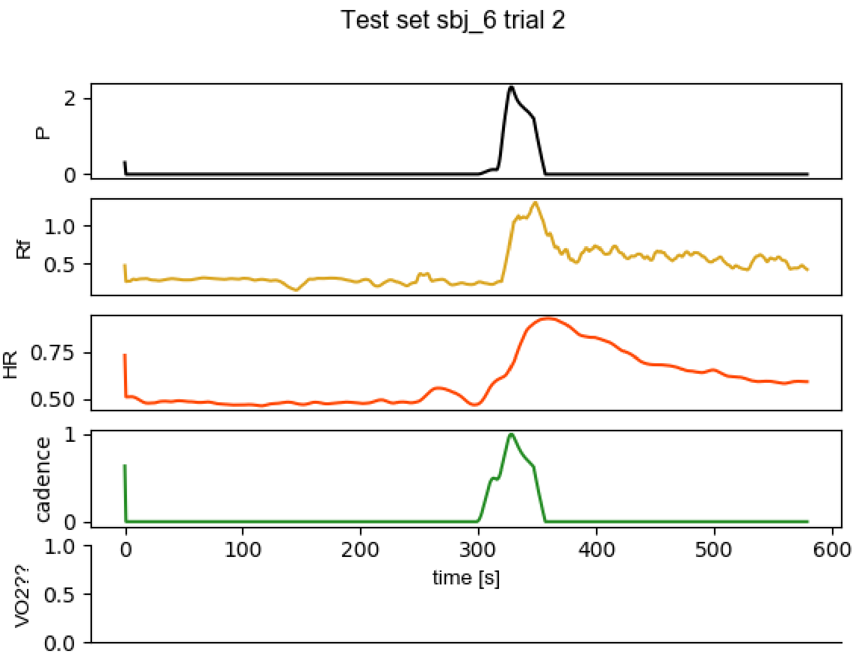

Figure 72: Test set include: power output (P, black), respiratory frequency (Rf, gold), heart rate (HR, red) and cadence (ω, green). The $\dot{V}O_2$ value is assumed to be unknown during the test set, therefore is left undefined in this plot. Every quantity is normalised on the maximal value reported in the individual $.json$ file.

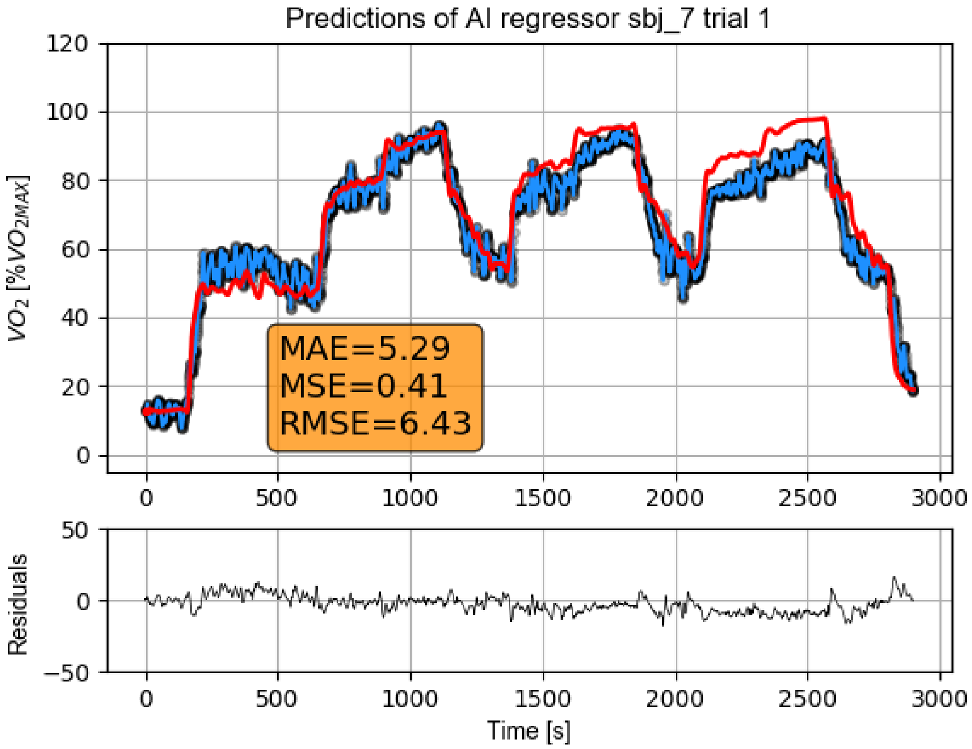

Figure 73: Oxygen uptake ($\dot{V}O_2$) experimental values (black semi-transparent dots and blue line) are compared to the estimated (red line) $\dot{V}O_2$ values. Residuals are computed as the difference between actual and estimated values. $\dot{V}O_2$ values are expressed as $\%$ of the individual's maximal $\dot{V}O_2$ (i.e. $\dot{V}O_{2MAX}$).

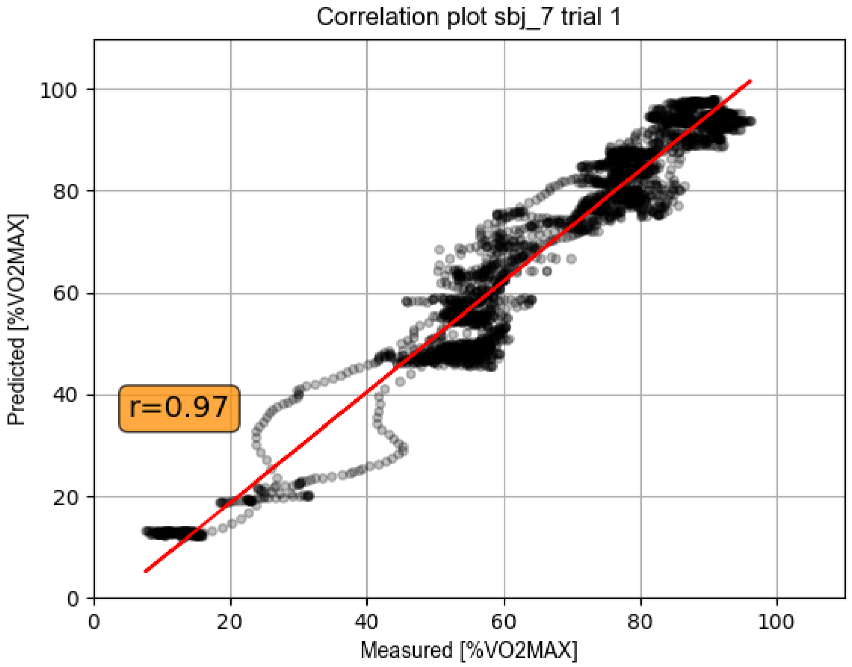

Figure 74: A scatterplot (semi-transparent black dots) that shows the relationship between actual and predicted $\dot{V}O_2$ responses. The red line represents the best-fit linear equation.

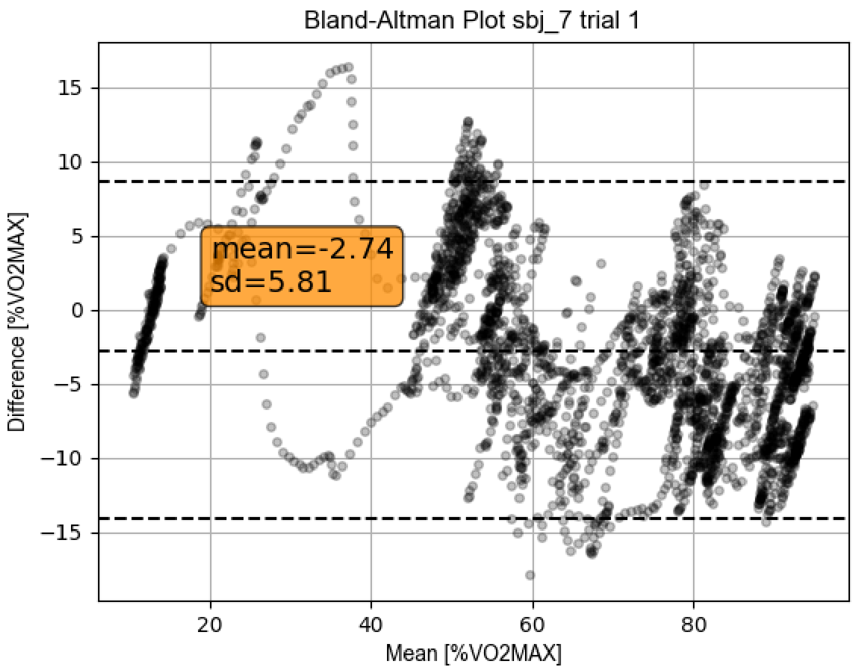

Figure 75: Bland-Altman plot is used to visually appreciate the agreement between actual and predicted $\dot{V}O_2$ responses.

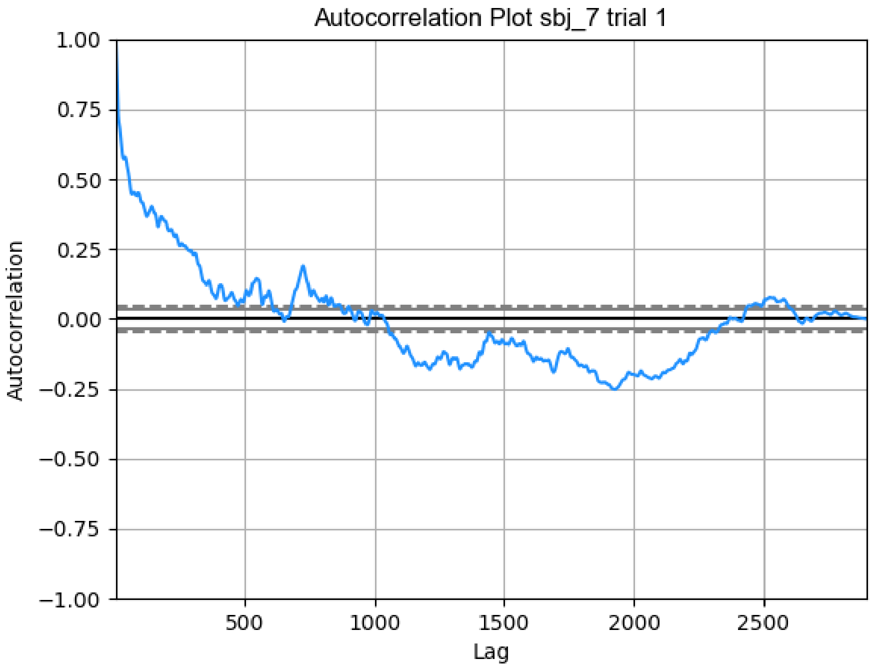

Figure 76: The autocorrelation plot (correlogram) is used to assess the correlation between the signal and the delayed (lagged) replication. Sample autocorrelation (blue line) is reported versus time lags. The horizontal (grey) lines displayed in the plot correspond to 95% and 99% (dashed) confidence bands.

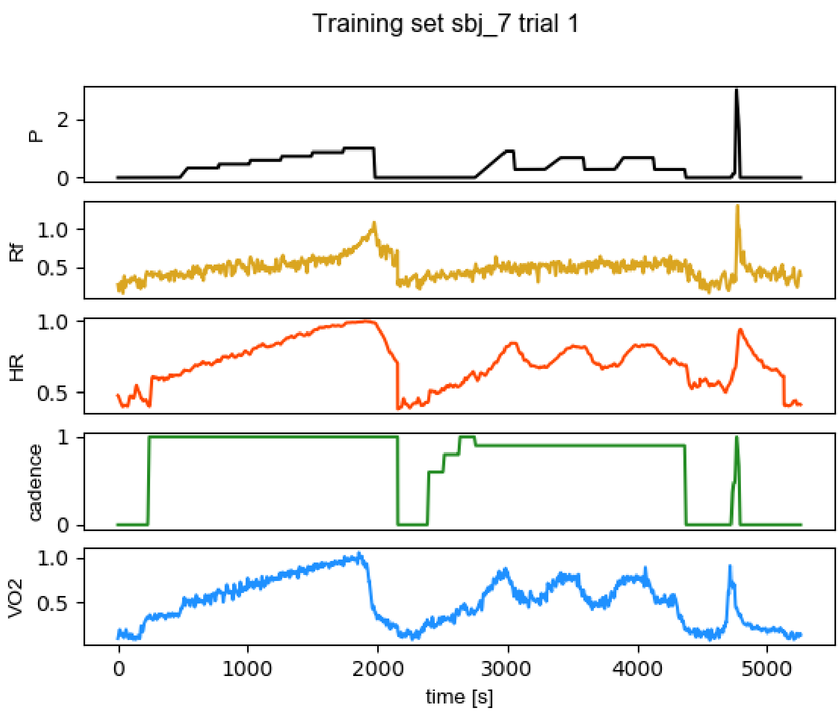

Figure 77: Training set include: power output (P, black), respiratory frequency (Rf, gold), heart rate (HR, red), cadence (ω, green) and $\dot{V}O_2$ (VO2, blue). Every quantity is normalised on the maximal value reported in the individual $.json$ file.

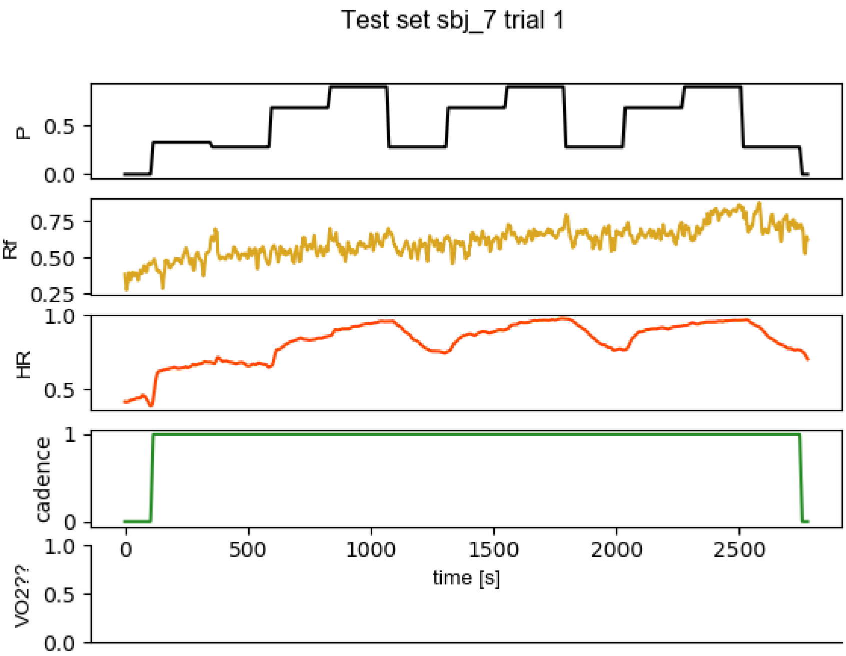

Figure 78: Test set include: power output (P, black), respiratory frequency (Rf, gold), heart rate (HR, red) and cadence (ω, green). The $\dot{V}O_2$ value is assumed to be unknown during the test set, therefore is left undefined in this plot. Every quantity is normalised on the maximal value reported in the individual $.json$ file.

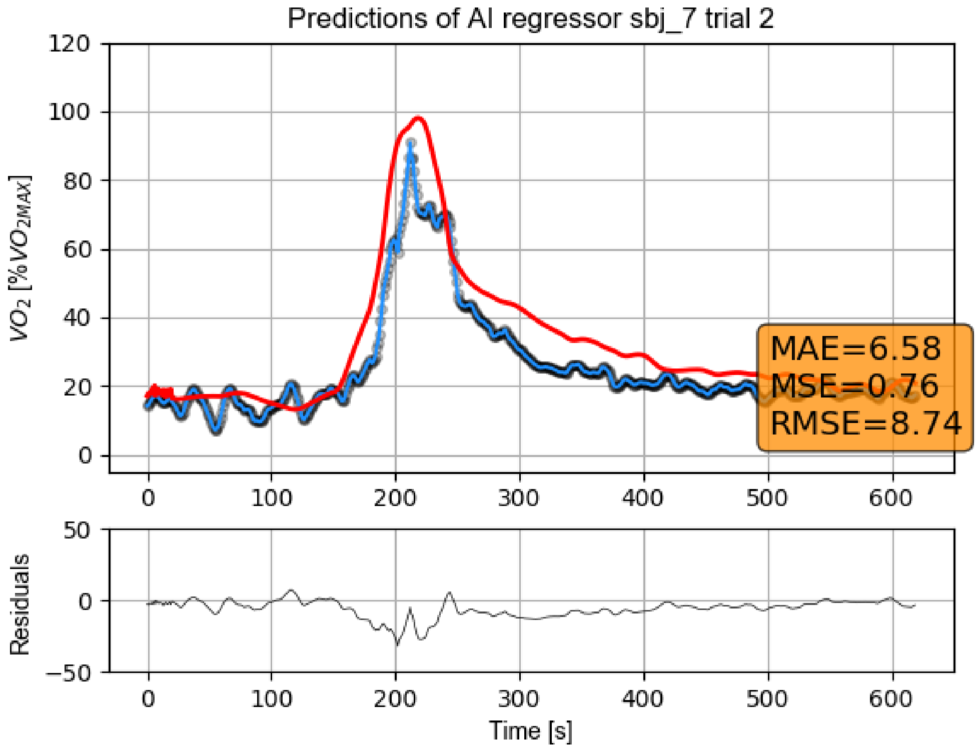

Figure 79: Oxygen uptake ($\dot{V}O_2$) experimental values (black semi-transparent dots and blue line) are compared to the estimated (red line) $\dot{V}O_2$ values. Residuals are computed as the difference between actual and estimated values. $\dot{V}O_2$ values are expressed as % of the individual's maximal $\dot{V}O_2$ (i.e. $\dot{V}O_{2MAX}$).

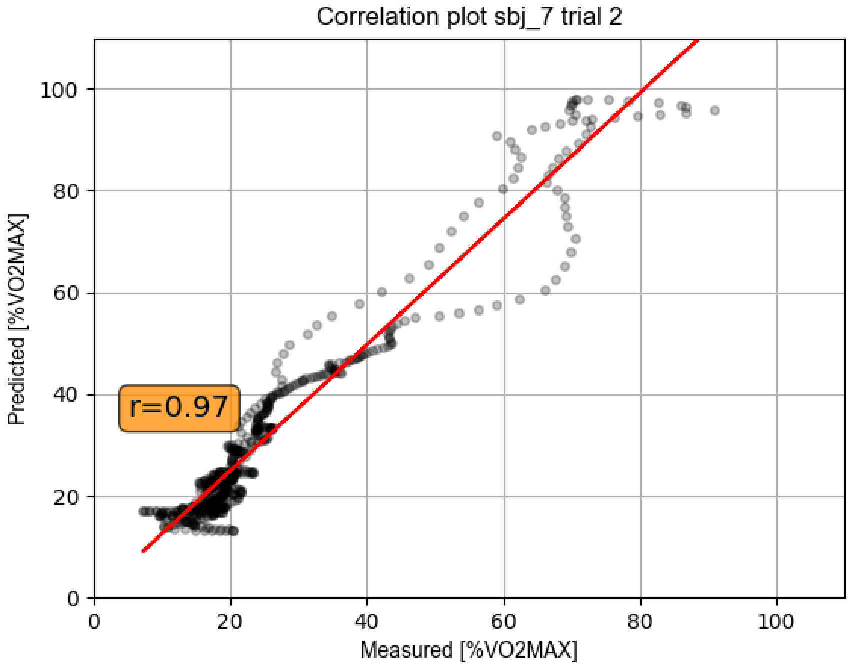

Figure 80: A scatterplot (semi-transparent black dots) that shows the relationship between actual and predicted $\dot{V}O_2$ responses. The red line represents the best-fit linear equation.

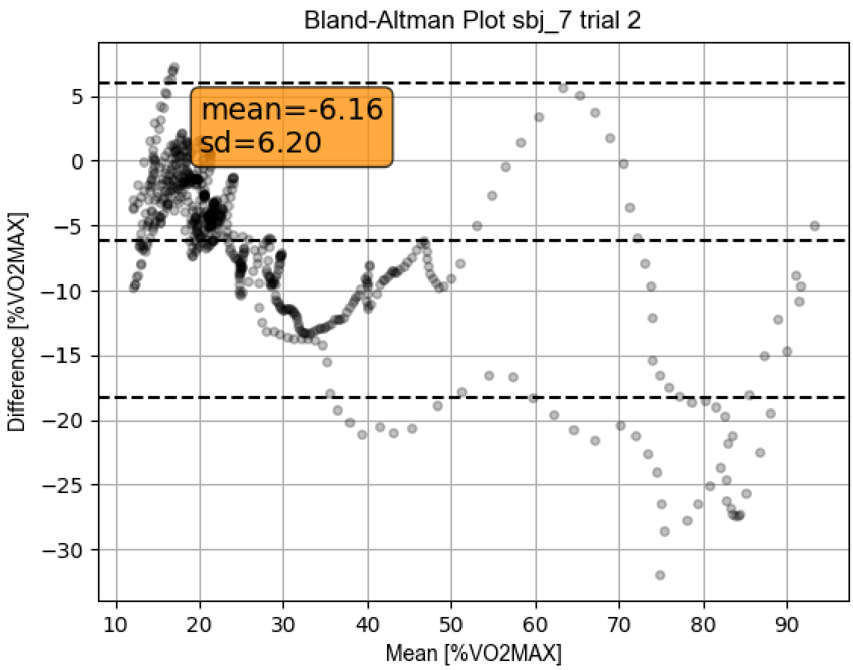

Figure 81: Bland-Altman plot is used to visually appreciate the agreement between actual and predicted $\dot{V}O_2$ responses.

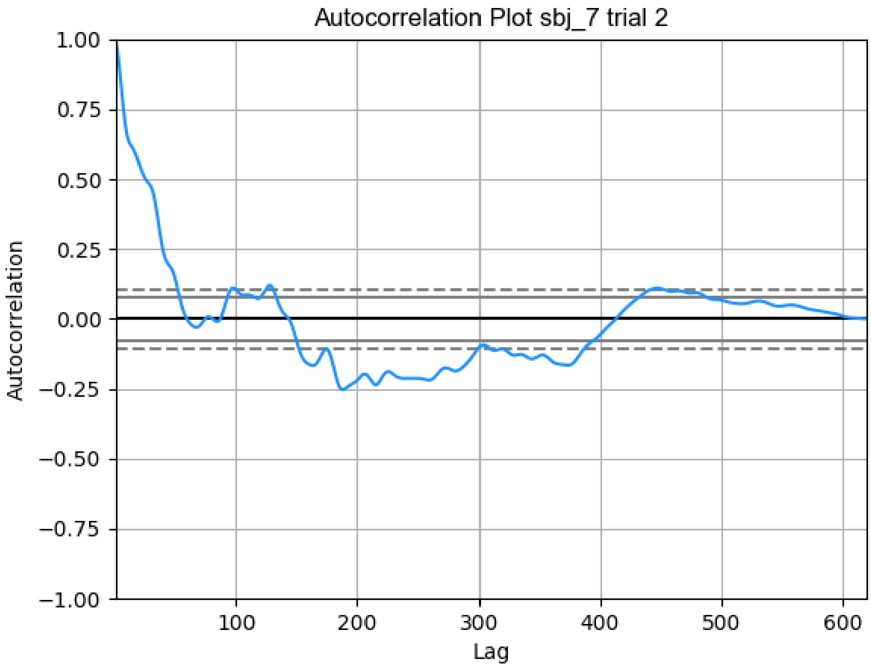

Figure 82: The autocorrelation plot (correlogram) is used to assess the correlation between the signal and the delayed (lagged) replication. Sample autocorrelation (blue line) is reported versus time lags. The horizontal (grey) lines displayed in the plot correspond to 95% and 99% (dashed) confidence bands.

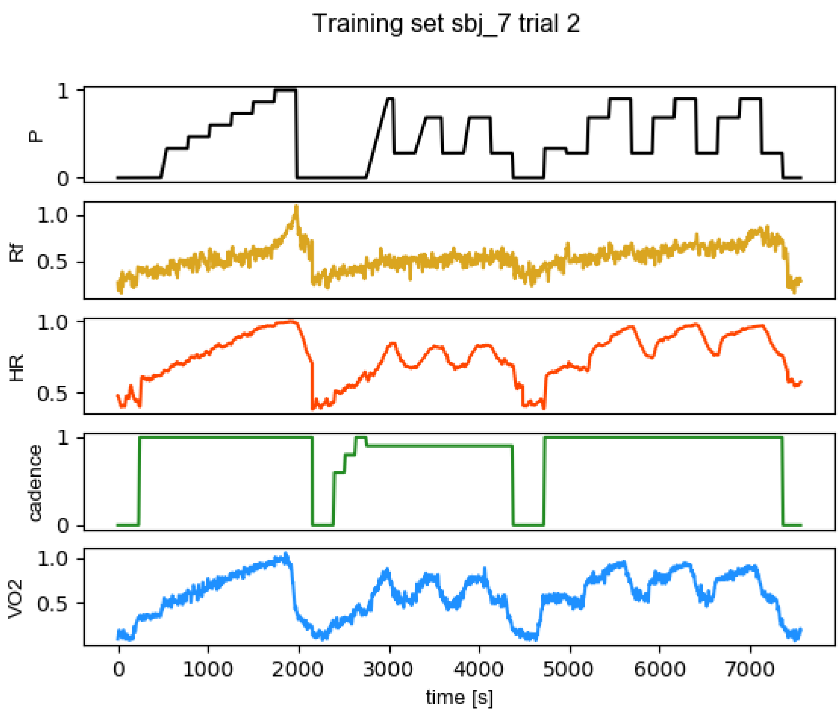

Figure 83: Training set include: power output (P, black), respiratory frequency (Rf, gold), heart rate (HR, red), cadence (ω, green) and $\dot{V}O_2$ (VO2, blue). Every quantity is normalised on the maximal value reported in the individual $.json$ file.

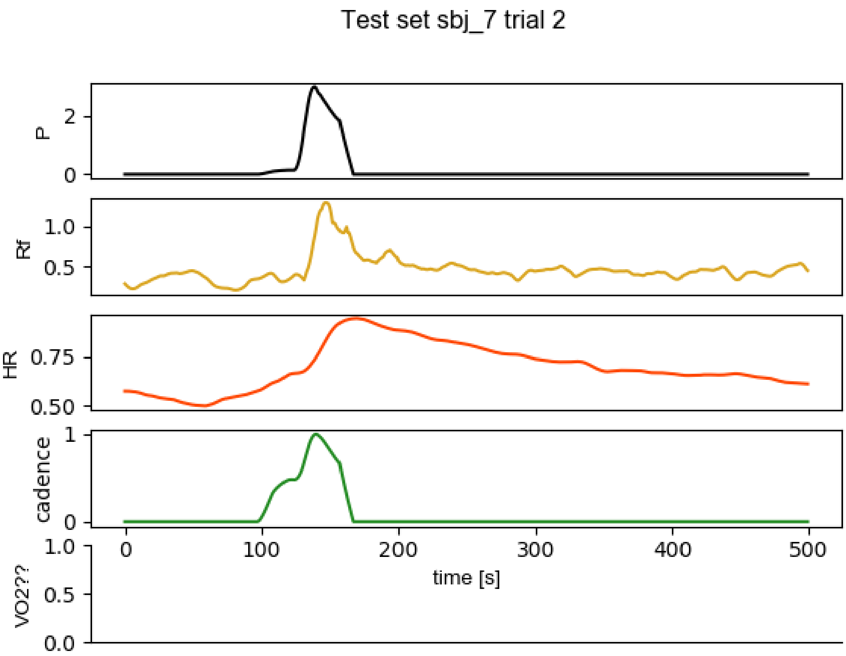

Figure 84: Test set include: power output (P, black), respiratory frequency (Rf, gold), heart rate (HR, red) and cadence (ω, green). The $\dot{V}O_2$ value is assumed to be unknown during the test set, therefore is left undefined in this plot. Every quantity is normalised on the maximal value reported in the individual $.json$ file.

4 Adding a new participant

In this section we report the results obtained by training the neural network with just an incremental-to-exhaustion test. This participant was not included in the main study. Here, we want to check whether the number of test needed to train the neural network can be reduced to a single test. This should reduce the time spent by the athletes in the laboratory. We tested the neural network on two different high-intensity interval training protocols.

3 Test a new participant

The neural network can be trained and tested with a new participant as follows:

(a) Collect and save the data in $.csv$ files.

(b) Create a $.json$ file and indicate maximal values (e.g. those obtained during the incremental to exhaustion test).

(c) Modify the code and include the input/output that you would like to use (e.g. you have other info like oxygen saturation or you would like to use RF as output).

(d) Train the neural network.

(e) Test the neural network.

The results of the predictions (i.e. residuals, correlation, autocorrelation and Bland-Altman) and the plots of the training/testing datasets are provided in the following figures.

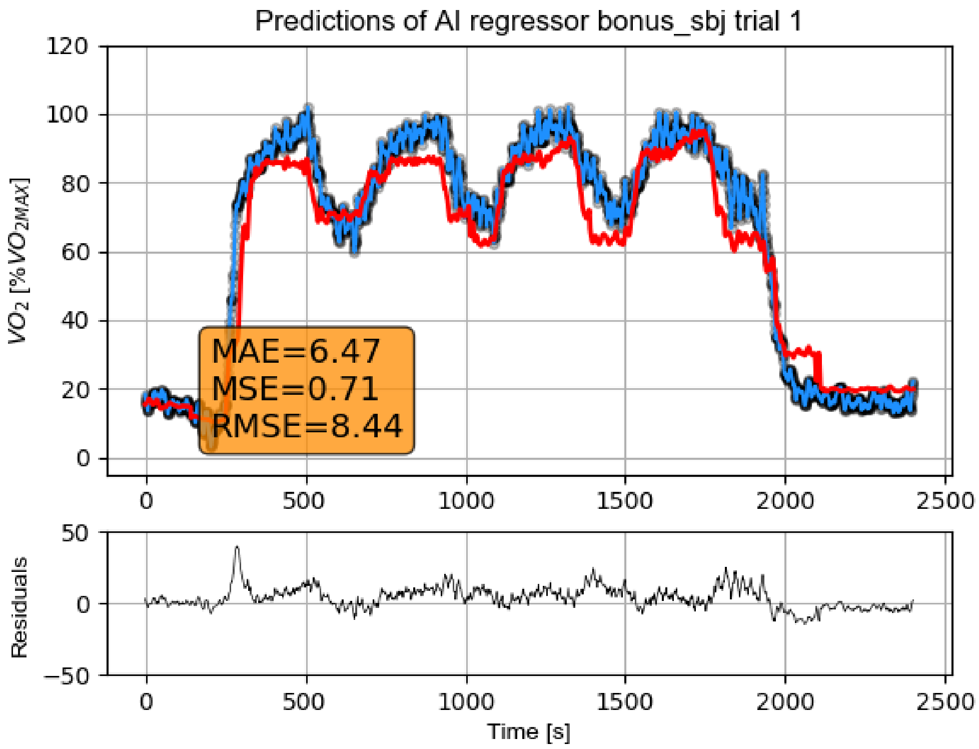

Figure 85: Oxygen uptake ($\dot{V}O_2$) experimental values (black semi-transparent dots and blue line) are compared to the estimated (red line) $\dot{V}O_2$ values. Residuals are computed as the difference between actual and estimated values. $\dot{V}O_2$ values are expressed as % of the individual's maximal $\dot{V}O_2$ (i.e. $\dot{V}O_{2MAX}$).

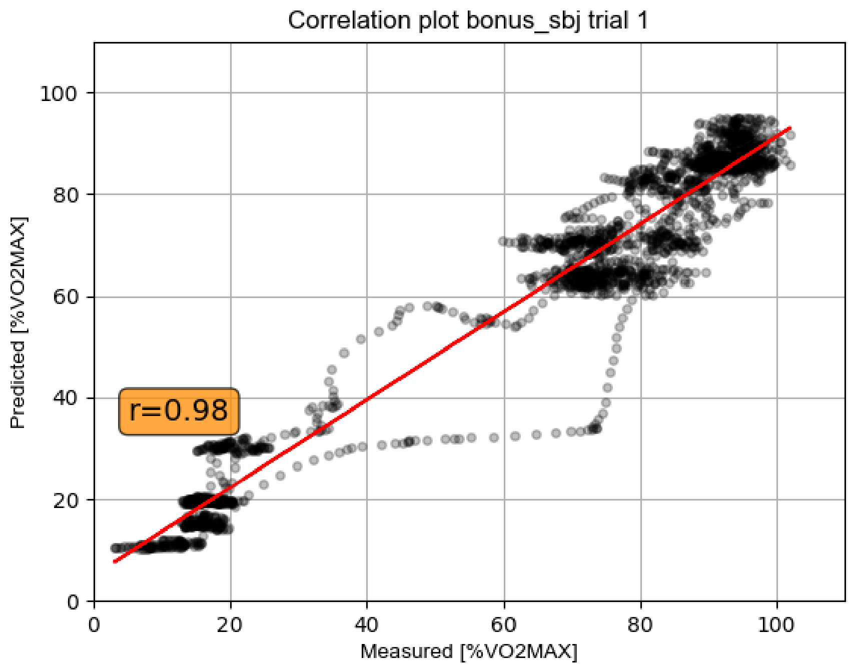

Figure 86: A scatterplot (semi-transparent black dots) that shows the relationship between actual and predicted $\dot{V}O_2$ responses. The red line represents the best-fit linear equation.

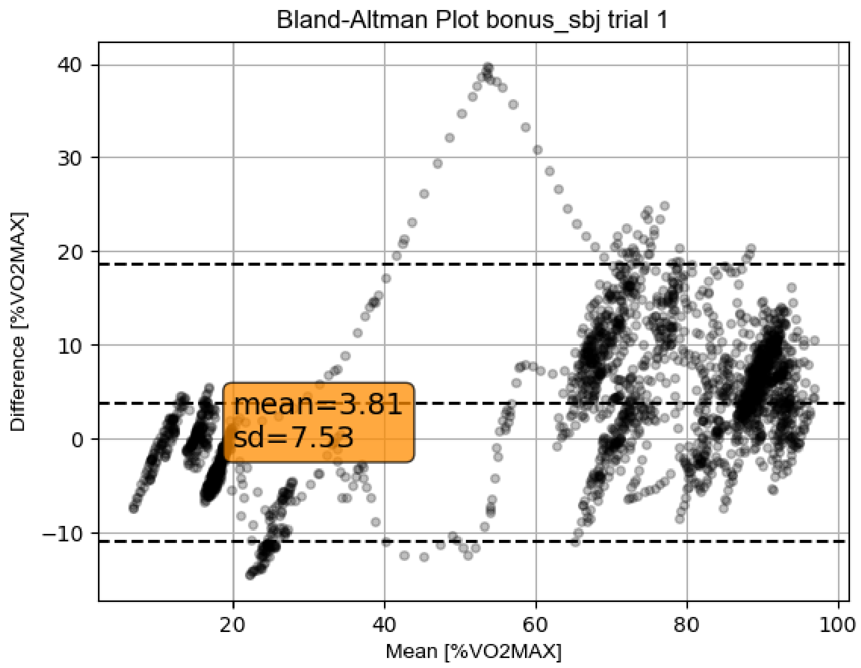

Figure 87: Bland-Altman plot is used to visually appreciate the agreement between actual and predicted $\dot{V}O_2$ responses.

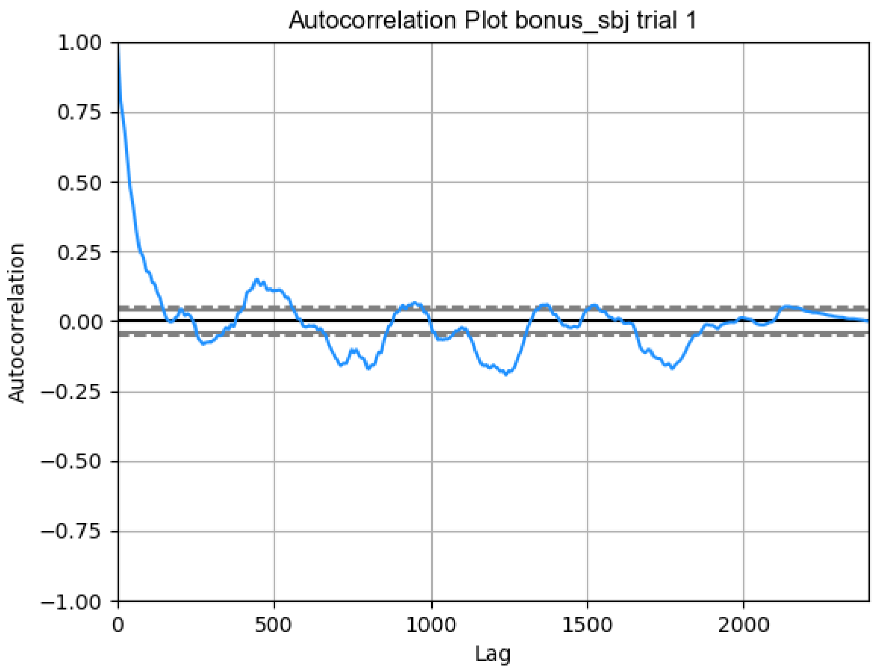

Figure 88: The autocorrelation plot (correlogram) is used to assess the correlation between the signal and the delayed (lagged) replication. Sample autocorrelation (blue line) is reported versus time lags. The horizontal (grey) lines displayed in the plot correspond to 95% and 99% (dashed) confidence bands.

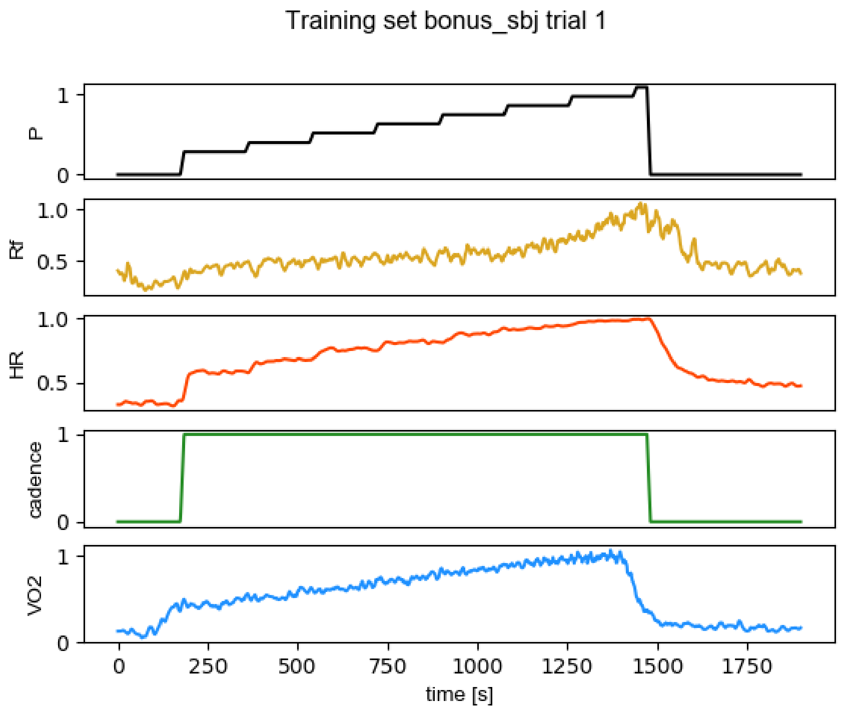

Figure 89: Training set include: power output (P, black), respiratory frequency (Rf, gold), heart rate (HR, red), cadence (ω, green) and $\dot{V}O_2$ (VO2, blue). Every quantity is normalised on the maximal value reported in the individual $.json$ file.

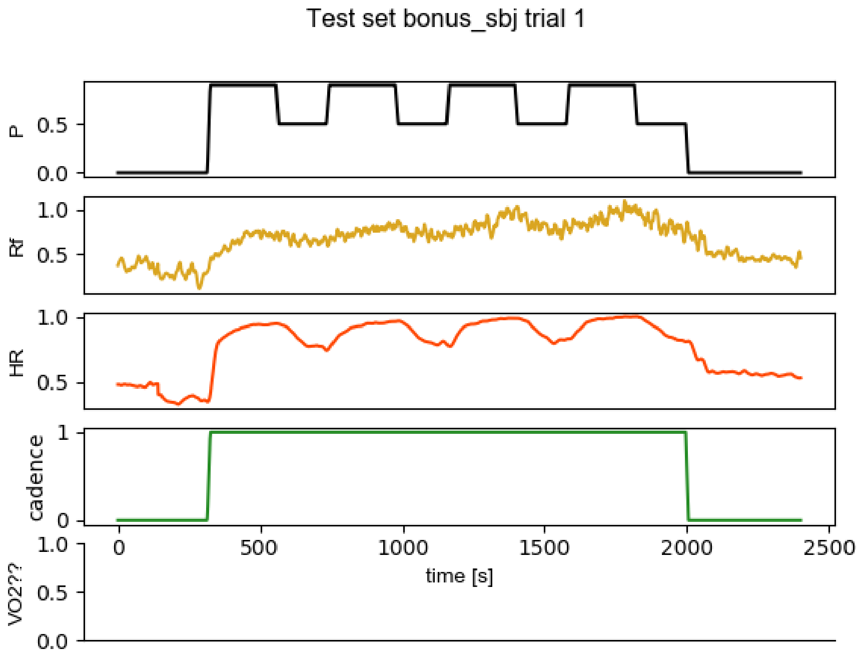

Figure 90: Test set include: power output (P, black), respiratory frequency (Rf, gold), heart rate (HR, red) and cadence (ω, green). The $\dot{V}O_2$ value is assumed to be unknown during the test set, therefore is left undefined in this plot. Every quantity is normalised on the maximal value reported in the individual $.json$ file.

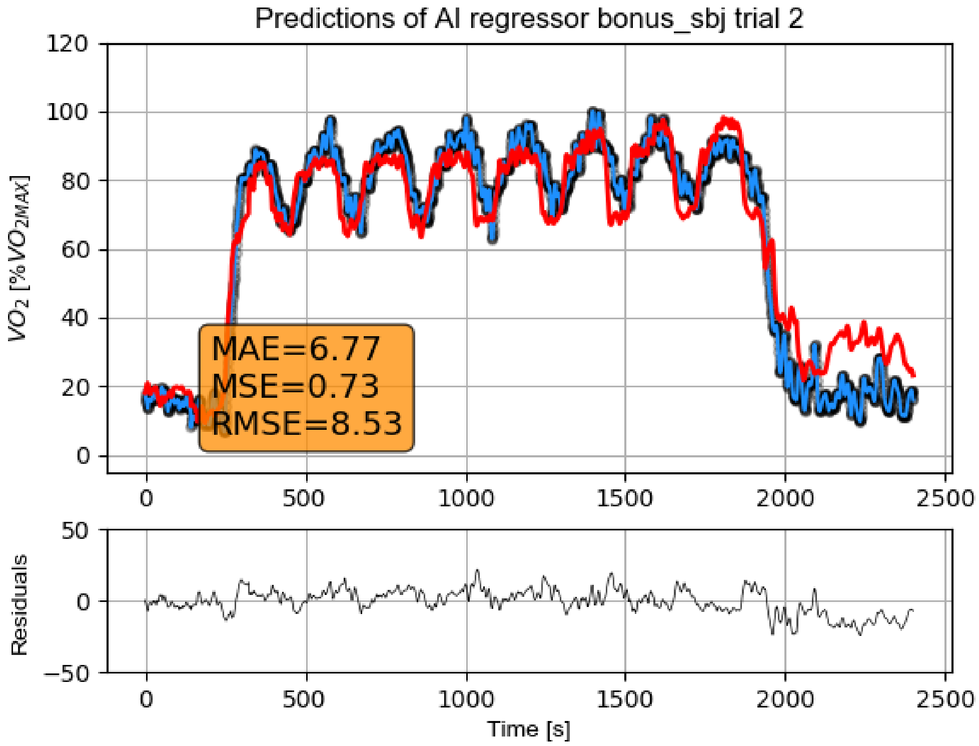

Figure 91: Oxygen uptake ($\dot{V}O_2$) experimental values (black semi-transparent dots and blue line) are compared to the estimated (red line) $\dot{V}O_2$ values. Residuals are computed as the difference between actual and estimated values. $\dot{V}O_2$ values are expressed as % of the individual's maximal $\dot{V}O_2$ (i.e. $\dot{V}O_{2MAX}$).

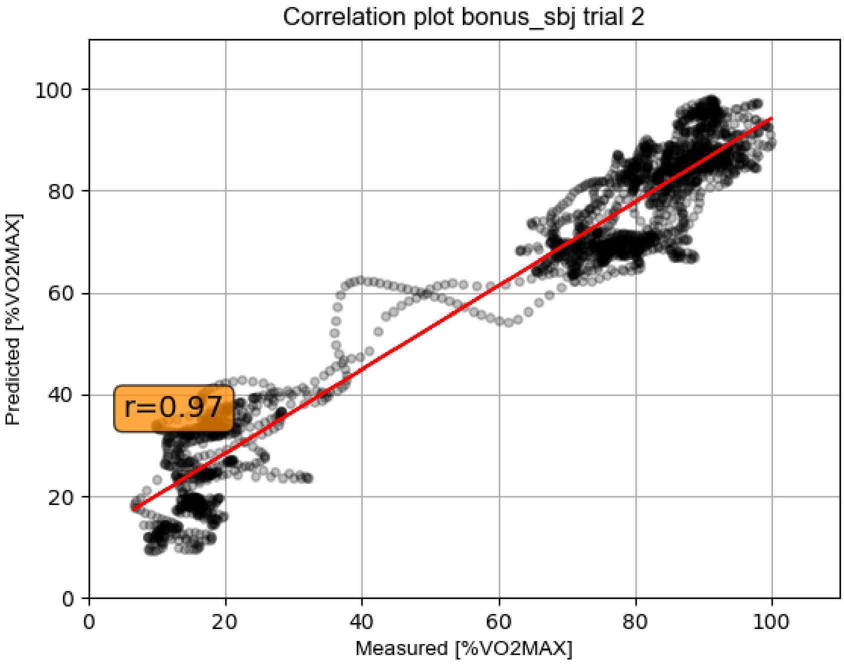

Figure 92: A scatterplot (semi-transparent black dots) that shows the relationship between actual and predicted $\dot{V}O_2$ responses. The red line represents the best-fit linear equation.

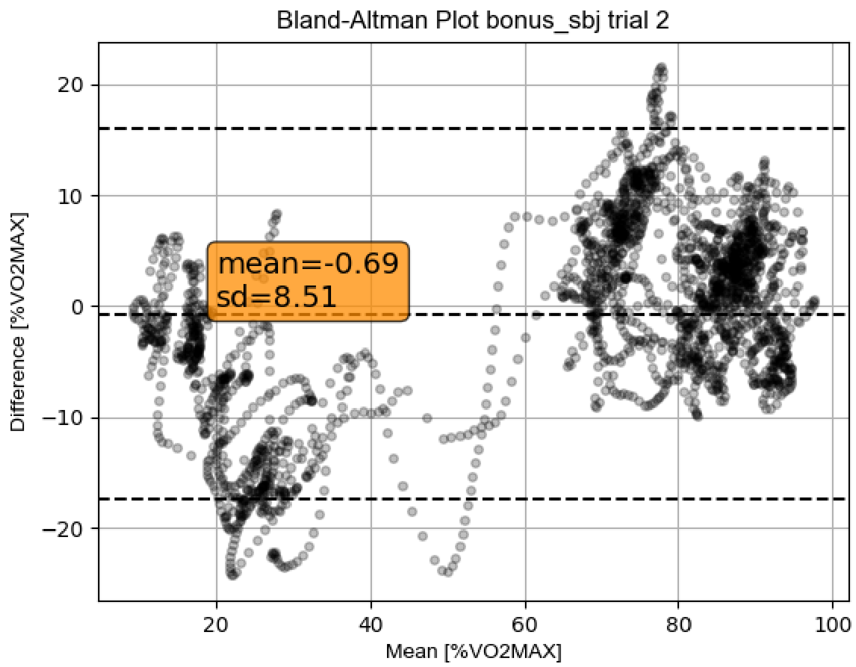

Figure 93: Bland-Altman plot is used to visually appreciate the agreement between actual and predicted $\dot{V}O_2$ responses.

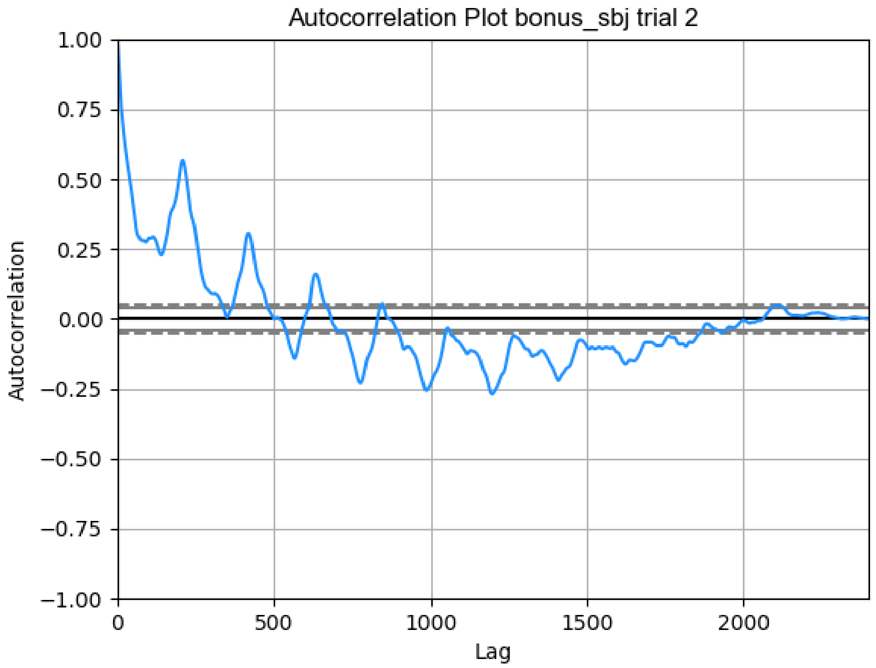

Figure 94: The autocorrelation plot (correlogram) is used to assess the correlation between the signal and the delayed (lagged) replication. Sample autocorrelation (blue line) is reported versus time lags. The horizontal (grey) lines displayed in the plot correspond to 95% and 99% (dashed) confidence bands.

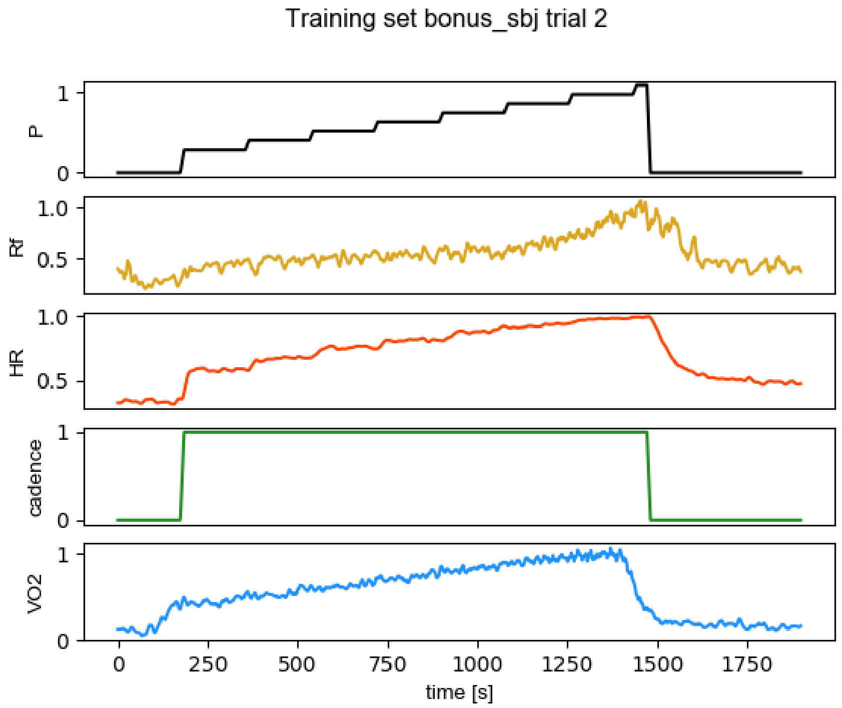

Figure 95: Training set include: power output (P, black), respiratory frequency (Rf, gold), heart rate (HR, red), cadence (ω, green) and $\dot{V}O_2$ (VO2, blue). Every quantity is normalised on the maximal value reported in the individual $.json$ file.

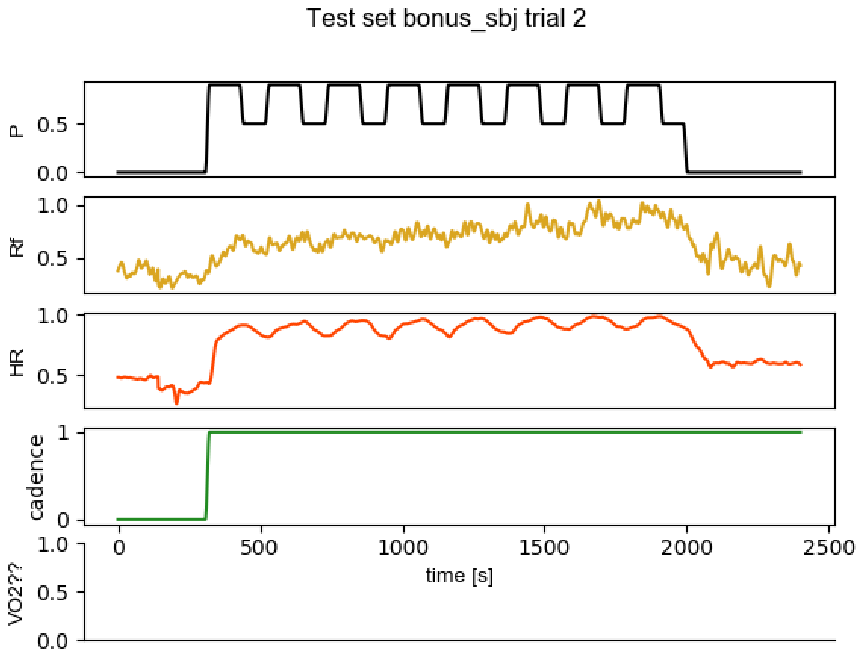

Figure 96: Test set include: power output (P, black), respiratory frequency (Rf, gold), heart rate (HR, red) and cadence (ω, green). The $\dot{V}O_2$ value is assumed to be unknown during the test set, therefore is left undefined in this plot. Every quantity is normalised on the maximal value reported in the individual $.json$ file.

5 Analytical models

In this section we report the results obtained during the validation of the analytical models (model 1 and 2).

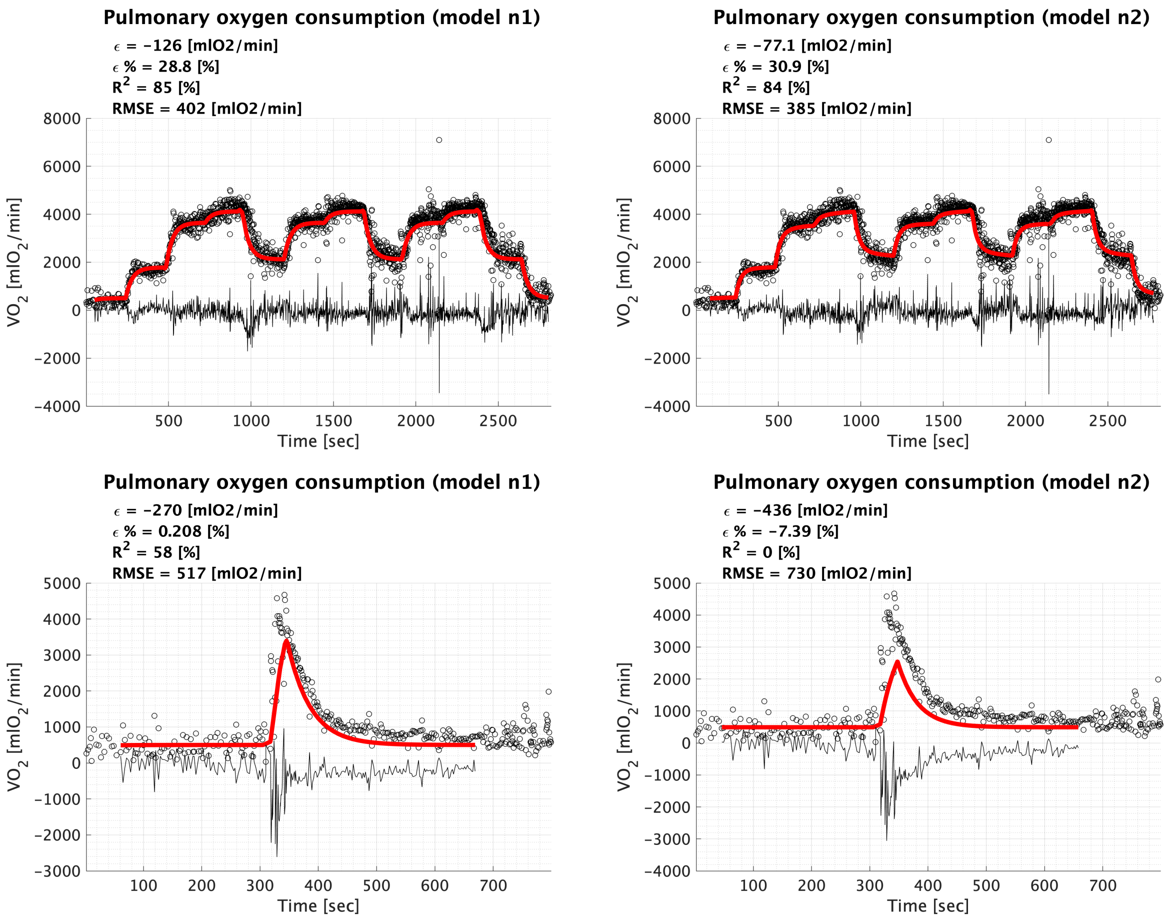

Figure 97: **Participant 1**: oxygen uptake ($\dot{V}O_2$) experimental values (black circles) are compared to the estimated (red line) $\dot{V}O_2$ values. Residuals are computed as the difference between actual and estimated values (solid line). Summary statistics such as: mean absolute error (ϵ), mean absolute percentage error ($\epsilon\%$), variance explained R^2 and root mean square error (RMSE), are also reported.

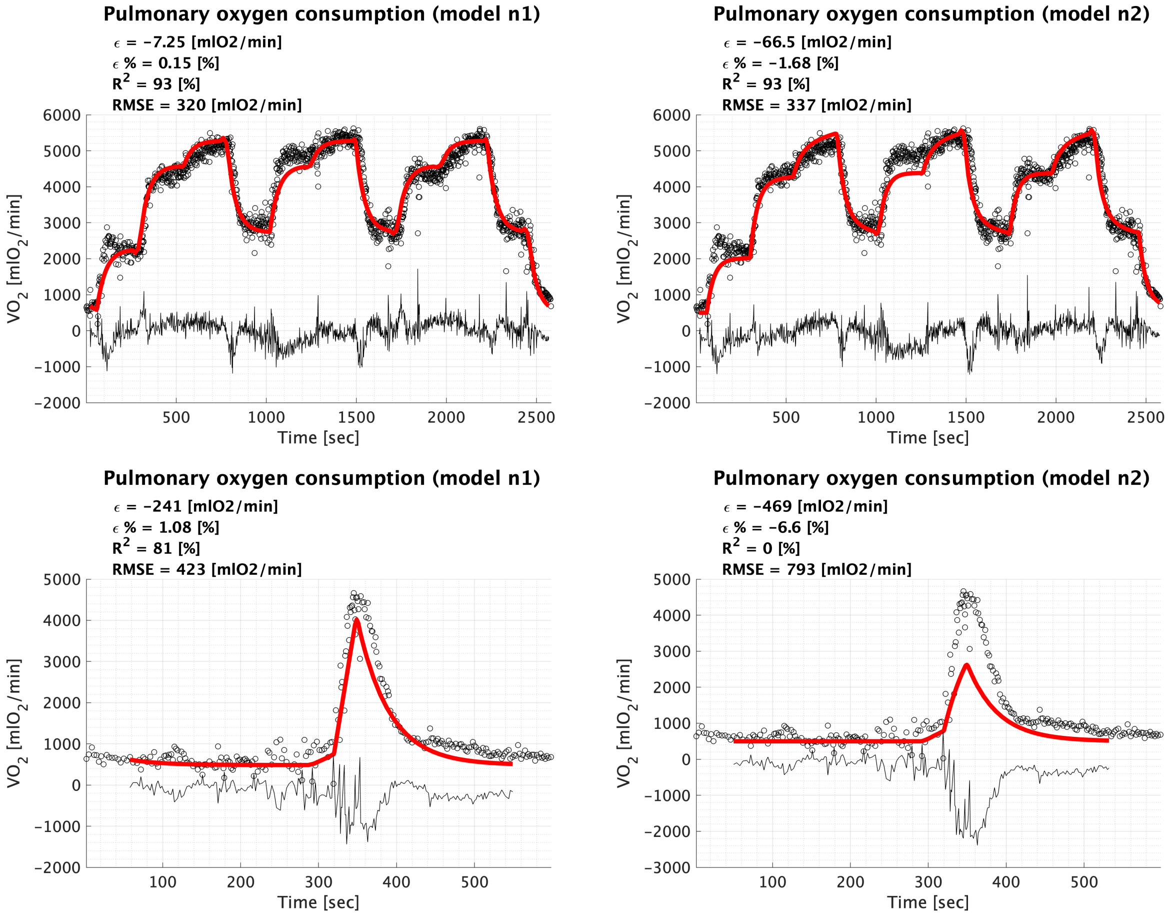

Figure 98: **Participant 2**: oxygen uptake ($\dot{V}O_2$) experimental values (black circles) are compared to the estimated (red line) $\dot{V}O_2$ values. Residuals are computed as the difference between actual and estimated values (solid line). Summary statistics such as: mean absolute error (ϵ), mean absolute percentage error ($\epsilon\%$), variance explained R^2 and root mean square error (RMSE), are also reported.

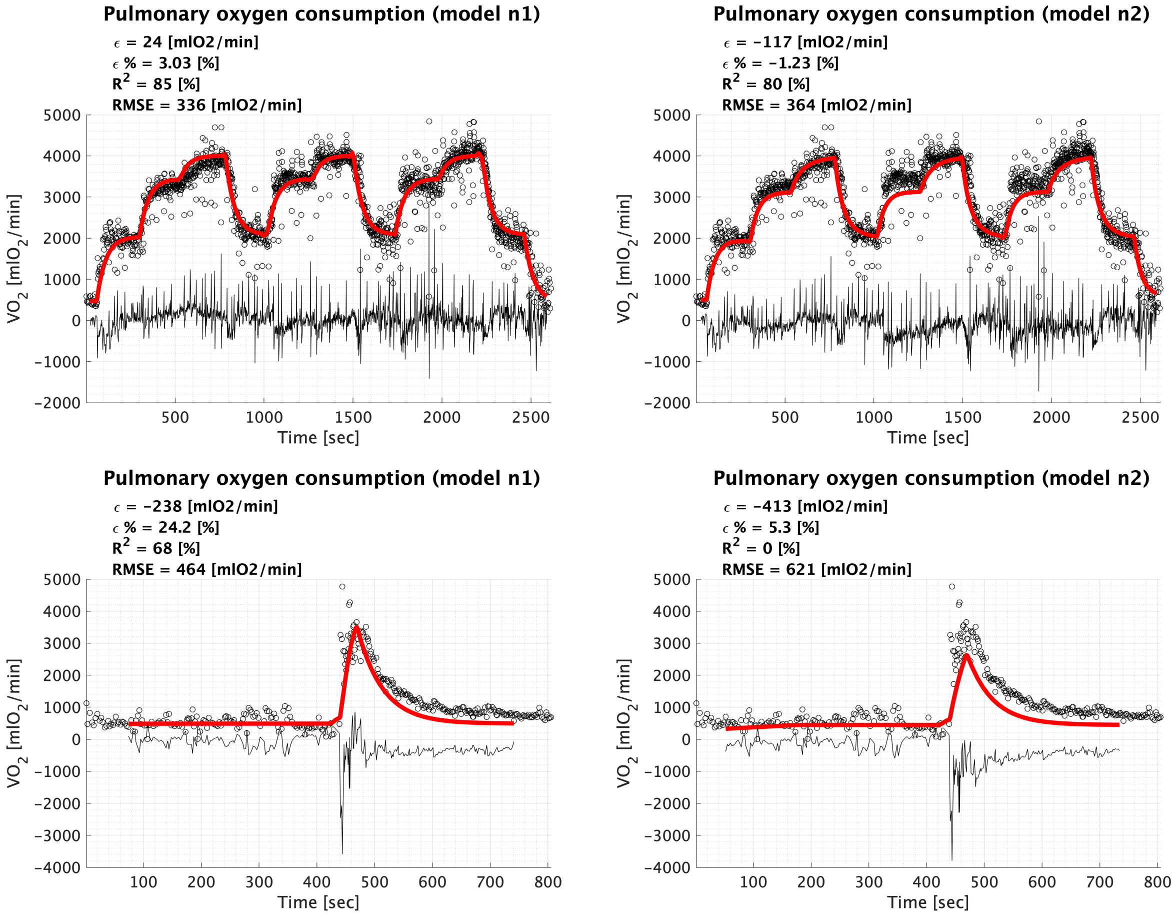

Figure 99: **Participant 3**: oxygen uptake ($\dot{V}O_2$) experimental values (black circles) are compared to the estimated (red line) $\dot{V}O_2$ values. Residuals are computed as the difference between actual and estimated values (solid line). Summary statistics such as: mean absolute error (ϵ), mean absolute percentage error ($\epsilon\%$), variance explained R^2 and root mean square error (RMSE), are also reported.

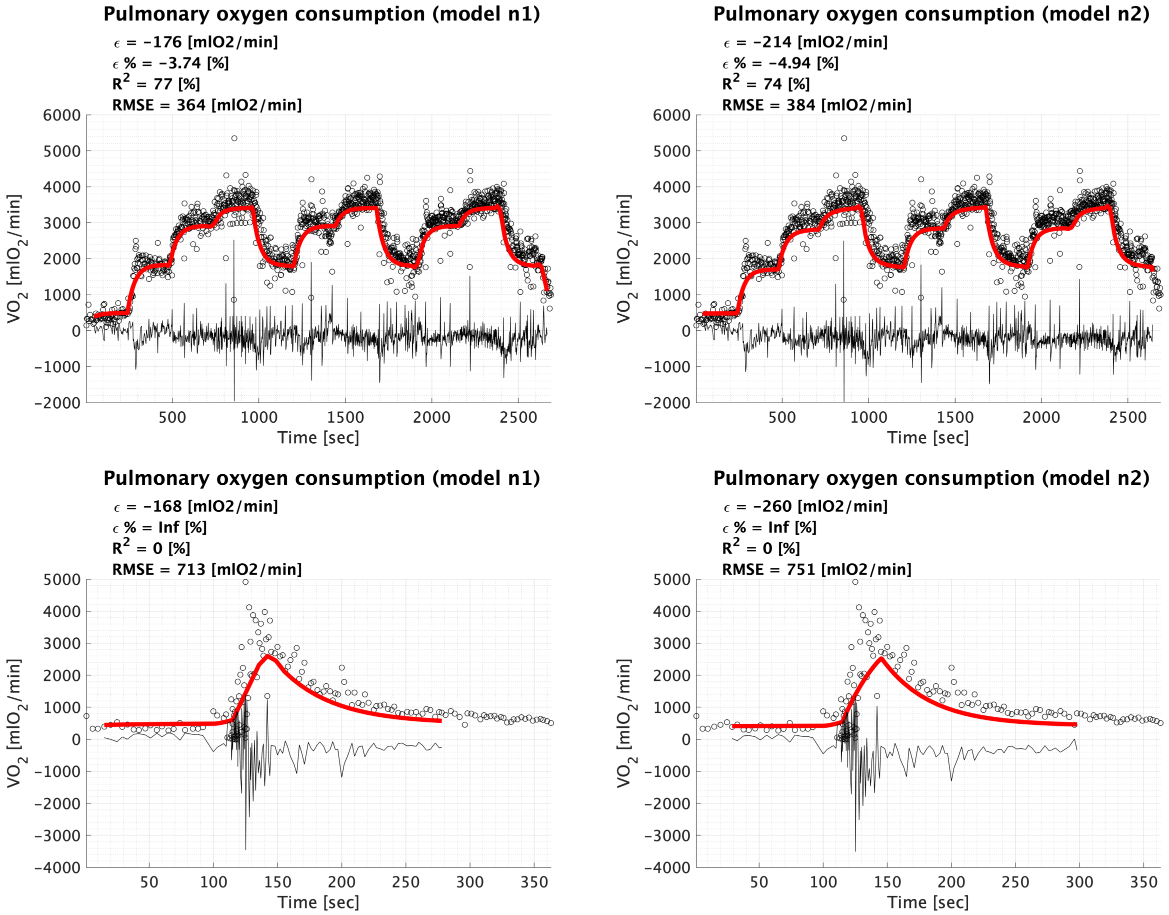

Figure 100: **Participant 4**: oxygen uptake ($\dot{V}O_2$) experimental values (black circles) are compared to the estimated (red line) $\dot{V}O_2$ values. Residuals are computed as the difference between actual and estimated values (solid line). Summary statistics such as: mean absolute error (ϵ), mean absolute percentage error ($\epsilon\%$), variance explained R^2 and root mean square error (RMSE), are also reported.

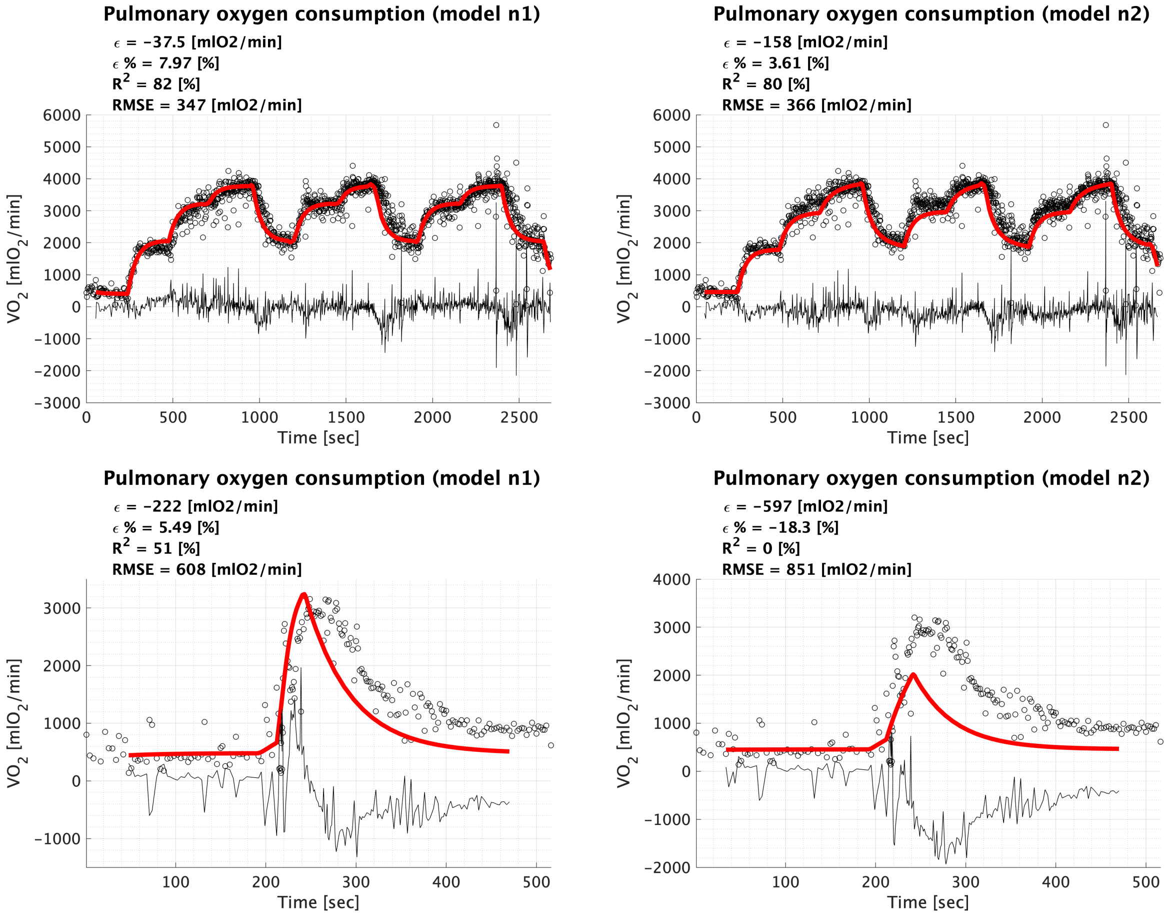

Figure 101: **Participant 5**: oxygen uptake ($\dot{V}O_2$) experimental values (black circles) are compared to the estimated (red line) $\dot{V}O_2$ values. Residuals are computed as the difference between actual and estimated values (solid line). Summary statistics such as: mean absolute error (ϵ), mean absolute percentage error ($\epsilon\%$), variance explained R^2 and root mean square error (RMSE), are also reported.

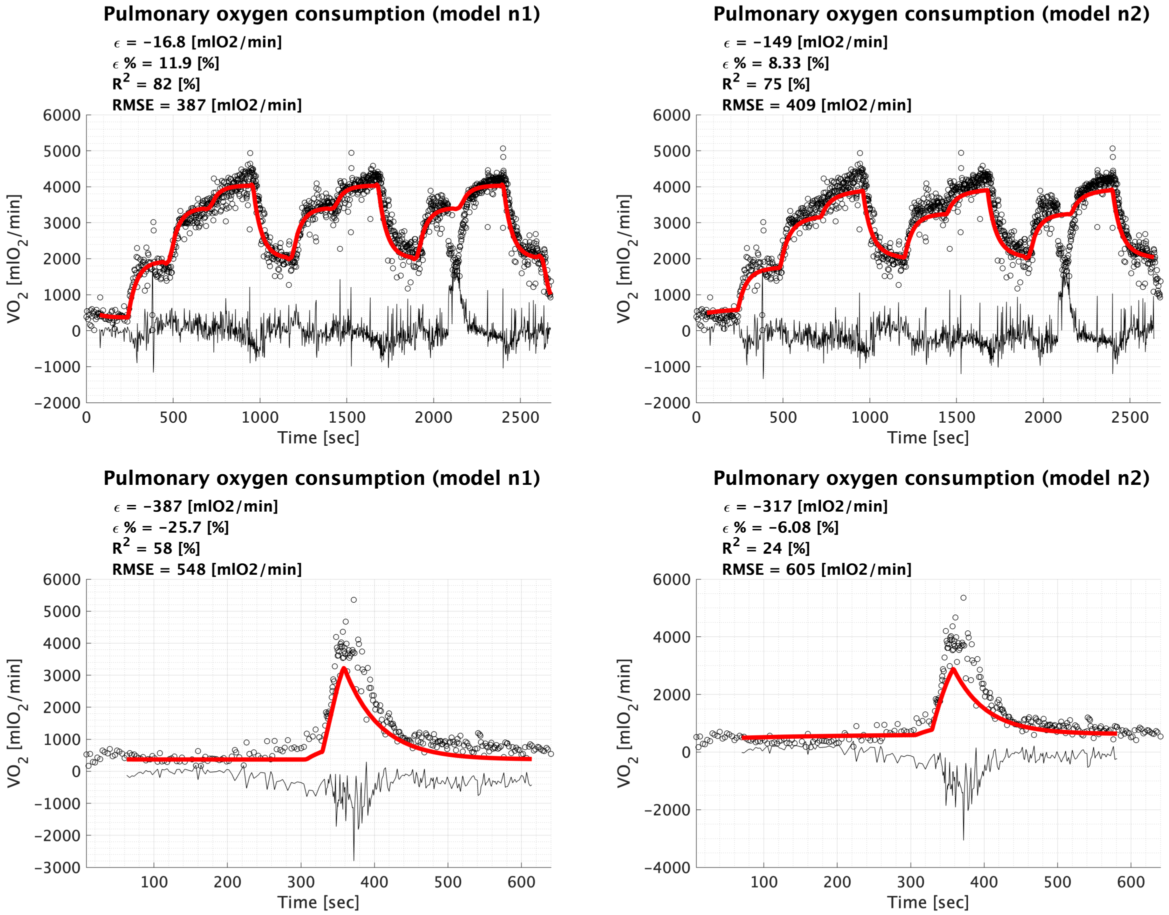

Figure 102: **Participant 6**: oxygen uptake ($\dot{V}O_2$) experimental values (black circles) are compared to the estimated (red line) $\dot{V}O_2$ values. Residuals are computed as the difference between actual and estimated values (solid line). Summary statistics such as: mean absolute error (ϵ), mean absolute percentage error ($\epsilon\%$), variance explained R^2 and root mean square error (RMSE), are also reported.

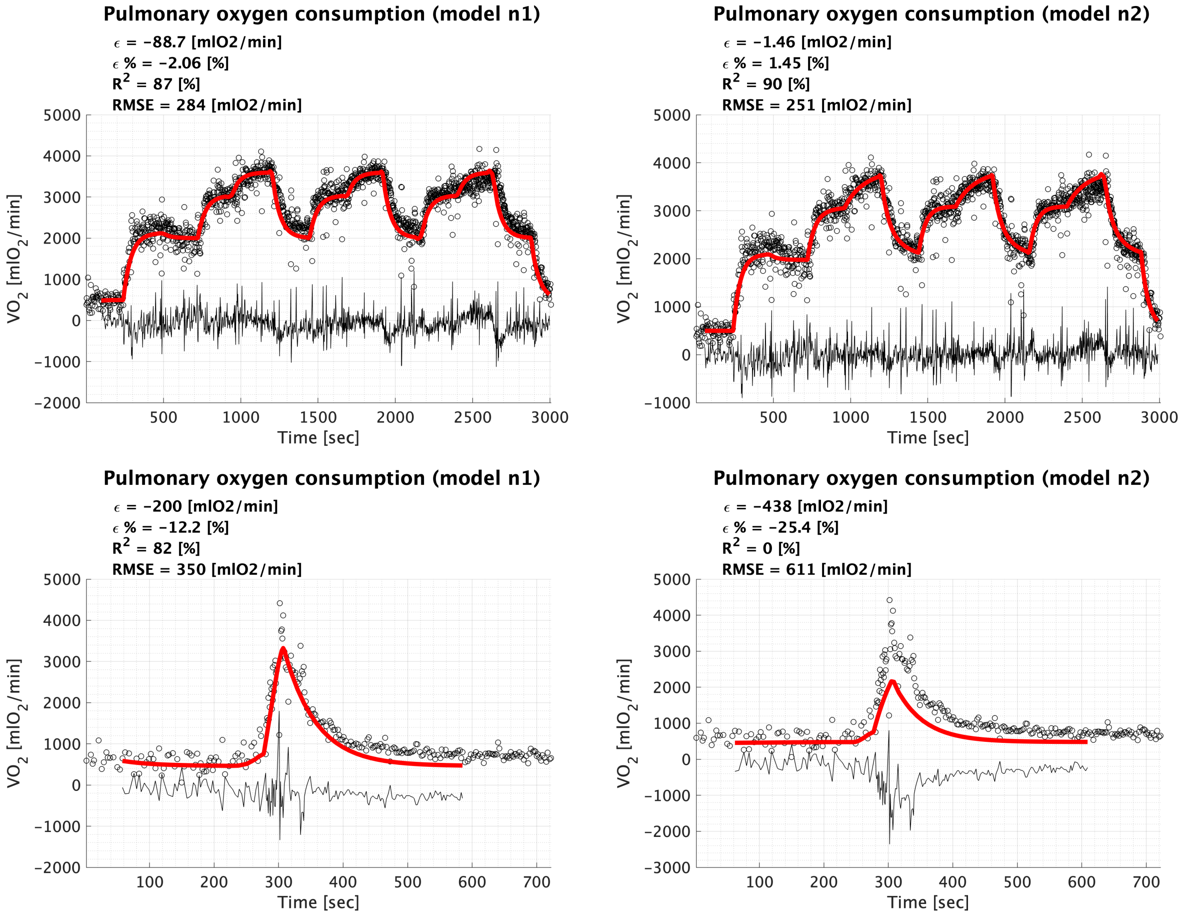

Figure 103: **Participant 7**: oxygen uptake ($\dot{V}O_2$) experimental values (black circles) are compared to the estimated (red line) $\dot{V}O_2$ values. Residuals are computed as the difference between actual and estimated values (solid line). Summary statistics such as: mean absolute error (ϵ), mean absolute percentage error ($\epsilon\%$), variance explained R^2 and root mean square error (RMSE), are also reported.
